# Membrane deformation supports ion conduction by the star of David [2]catenane

**DOI:** 10.1039/d6ra06238e

**Published:** 2026-11-03

**Authors:** Eden J. Gage, Philip C. Biggin

**Affiliations:** a Structural Bioinformatics and Computational Biochemistry, Department of Biochemistry South Parks Road Oxford OX1 3QU UK Philip.biggin@bioch.ox.ac.uk

## Abstract

Artificial ion channels (AICs) provide synthetic models of membrane transport and have potential therapeutic applications, yet the molecular structures of their conductive membrane states can be difficult to establish experimentally. The (Fe^2+^)_6_ Star of David [2]Catenane (SoD) forms highly conductive chloride channels despite a substantial mismatch with bilayer thickness. Here, atomistic molecular dynamics simulations were used to investigate possible membrane–embedded structures and conduction mechanisms of SoD. Pre-embedded, horizontally oriented SoD molecules stabilised hydrated, lipid-lined toroidal pores accompanied by pronounced local membrane deformation, producing continuous hourglass-shaped pathways for chloride permeation. Under applied transmembrane potentials between ±100 mV, SoD catenanes embedded in POPC : cholesterol (2 : 1) bilayers exhibited a conductance of 1.34 ± 0.14 nS, with conductance changes associated with SoD embedding depth and tilt and by lipid occlusion of the pore. Alternative vertically oriented monomers were unstable under the simulated conditions and reoriented towards horizontal configurations, whereas stacked SoD dimers remained conductive. Simulations using pre-formed electropores identified membrane defects as one possible route by which SoD could access embedded configurations, although these simulations do not establish the spontaneous insertion pathway under experimental conditions. Together, the results support a mechanism in which SoD can conduct chloride without itself spanning the bilayer by stabilising a lipid-lined membrane defect. More broadly, this mechanism illustrates how membrane deformation could contribute to conduction by synthetic channels whose molecular dimensions are shorter than the membrane thickness.

## Abstract 2

Artificial ion channels (AICs) provide synthetic models of membrane transport and have potential therapeutic applications, yet the molecular structures of their conductive membrane states can be difficult to establish experimentally.

## Graphical abstract

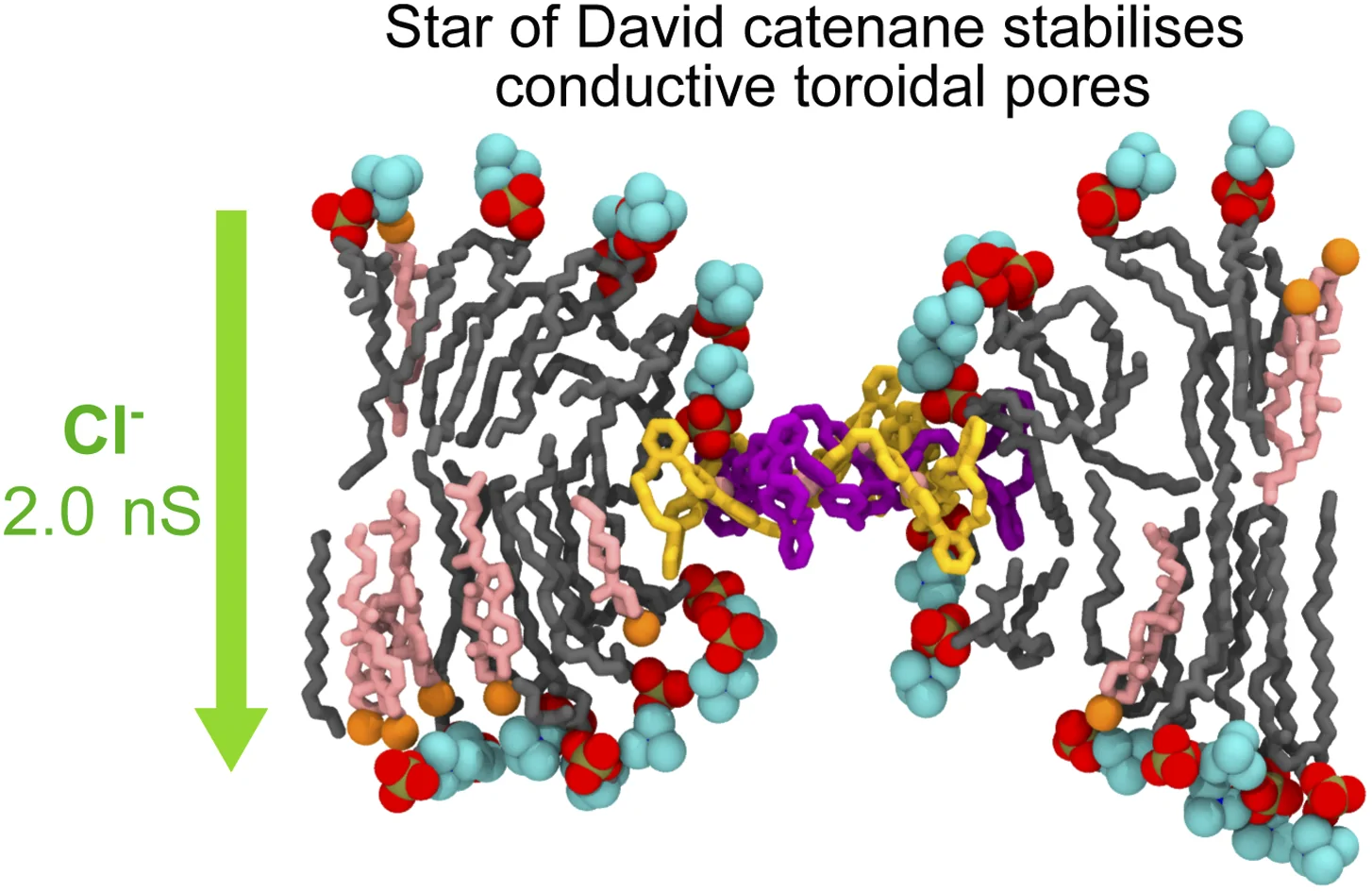

## Introduction

Ion transport through transmembrane protein channels is essential for maintaining physiological ion gradients across biological membranes, and their dysfunction can result in a wide range of channelopathies such as cystic fibrosis and epilepsy.^1^ Artificial ion channels (AICs) are *de novo* compounds that insert into lipid bilayers as functional biomimetics of natural protein channels and are increasingly recognised as promising therapeutic agents for the treatment of channelopathies, cancer, and for antimicrobial applications.^2–5^ To date, extensive research within the field has focused on the structural and functional capacities to which such artificial channels can be designed and as such has provided simplified ion channel models for the investigation of the fundamental principles and biophysics underlying ion transport across membranes.^6^

From these studies, conventional design rules have emerged that emphasise lipophilicity and ion recognition as key determinants of membrane insertion, selectivity, and ion transport.^7^ A further common heuristic is that AIC ensembles should span the full thickness of the lipid bilayer to support conduction.^7,8^ Accordingly, many systems are engineered either as large supramolecular assemblies or as monomeric scaffolds bearing substituents to form membrane–spanning architectures, and these motifs have been extensively reviewed elsewhere.^7,9^ As a result, such systems are often structurally complex and rely on specific intra- and inter-molecular interactions to maintain stability within membrane environments.^8^ However, the mechanisms by which they insert into membranes and organise into functional pores remain poorly understood. Membrane–spanning supramolecular design is often emphasised even when multimeric structural models are inferred indirectly, frequently from solid-state structures or gas-phase computational optimisations.

Recognition that the membrane is extremely plastic allows the accessible design space for channel architectures to be broader than is often assumed and need not be limited to larger, chemically complex transmembrane constructs. Indeed, simple non-membrane-spanning channel-forming AICs have been reported.^10–12^ Using experimental approaches, Fyles proposed that monomeric oligo-ester bola-amphiphiles embedded in a single leaflet could perturb the opposing leaflet sufficiently to permit water-channel formation and subsequent ion conduction.^10^ Similarly, Burkhardt's molecular dynamics (MD) simulations suggested that the previously reported conductive synthetic chloride membrane transporter (SCMTR) dimers embedded in a single-leaflet could induce water-channel formation to support ion conduction.^12^ Together, these notable studies suggest that the ability to span the full width of an unperturbed bilayer may not be strictly required in all cases.

The supramolecular organometallic (Fe^2+^)_6_ star of David [2]catenane (hereafter abbreviated to “SoD”) is a highly conductive monomeric synthetic chloride channel, despite possessing dimensions substantially shorter than the hydrophobic thickness of a typical lipid bilayer.^13^ Although now widely discussed as an AIC, the SoD was originally synthesised by the Leigh group in work aimed at constructing a previously elusive triply interlocked molecular link.^14^ Given its lipophilic peripheral surface and reported chloride-binding ability in solution, SoD was later investigated as a potential membrane chloride transporter, and strong channel activity was observed, although the mechanism of ion conduction remains unclear.^13^ Together with the examples provided by those such as Fyles^10^ and Burkhardt,^12^ SoD may suggest that the accessible design space for synthetic ion channels is broader than conventionally assumed. Accordingly, SoD provides a useful model system for investigating ion conduction in non-spanning architectures.

Here, we use atomistic MD simulations to complement existing experimental data and investigate proposed membrane–embedded models of SoD channels, with the aim of elucidating their likely conduction mechanisms. We find that SoD initially positioned vertically reorients towards horizontal configurations under the conditions examined, while pre-embedded horizontal SoD stabilises hydrated, toroidal, lipid-lined pores that form continuous pathways for chloride permeation. Using applied transmembrane potentials, these assemblies exhibit a range of conductive states associated with SoD position and membrane rearrangement. Finally, simulations with pre-formed electropores demonstrate that SoD can access embedded configurations through an existing membrane defect under the conditions examined. Together, these findings support a SoD-specific model in which membrane deformation enables chloride conduction without the molecular scaffold itself spanning the bilayer.

## Methods

### System construction and parameterisation

The Star of David [2]catenane (SoD; two neutral macrocyclic ligands and six Fe(ii) ions, net charge +12 e) was taken from the deposited crystal structure (CCDC 992376).^14^ Ligand parameters were assigned by analogy using CGenFF (v2.5.1)^15,16^*via* the CHARMM-GUI Ligand Reader and Modeler^17^ and Fe(ii) Lennard-Jones parameters were taken from the CHARMM divalent ion set;^18^ parameter penalties and structural validation are detailed in SI Section S1.1, S1.2, Tables S1–S8 and Fig. S1–4. Because Fe(ii)–N coordination is not represented by CHARMM bonded terms, the six Fe–N_6_ centres were maintained using 36 harmonic Fe–N distance restraints with crystallographic equilibrium distances and a force constant of 226 103.36 kJ mol^−1^ nm^−2^, equivalent to the CHARMM36 heme Fe–N bond term^19^ (SI Table S5).

### Membrane systems

For monomeric systems, SoD was embedded using the CHARMM-GUI^20^ (v3.7) Membrane Builder^21^ into the midplane of one of 5 bilayers: mixed 1-palmitoyl-2-oleoyl-*sn*-glycero-3-phosphocholine/cholesterol (POPC : CHL1, 67 : 33 mol%) or pure bilayers comprised of either 1,2-didecanoyl-*sn*-glycero-3-phosphocholine (DDPC, 10 : 0/10 : 0), 1,2-diundecanoyl-*sn*-glycero-3-phosphocholine (DCPC, 11 : 0/11 : 0), 1,2-didodecanoyl-*sn*-glycero-3-phosphocholine (DLPC, 12 : 0/12 : 0), or 1,2-dimyristoyl-*sn*-glycero-3-phosphocholine (DMPC, 14 : 0/14 : 0). All systems were solvated with 1 M KCl, with Cl^−^ ions added to neutralise the SoD charge. Vertical systems had the catenane plane parallel to the membrane normal whilst horizontal systems had it perpendicular (*i.e.* parallel to the bilayer plane). In both cases SoD was embedded at the midplane of bilayers. For the dimeric system, an initial model was constructed from two SoD catenanes with centres of mass (COM) vertically separated by 23.5 Å and rotationally offset by 30° about the membrane normal, and inserted into a POPC : cholesterol (2 : 1) bilayer using CHARMM-GUI as per the monomeric systems. All systems used six independently generated replicas, unless otherwise stated (SI Tables S9 and S10). For conductance (control) simulations, empty membranes were simulated using identical simulation protocols in the absence of SoD.

### Simulation parameters

Simulations were performed with GROMACS 2021.5 (GPU-accelerated, CUDA 11.7)^22,23^ using the CHARMM36 (2022, July)^24^ force field for lipids, ions and water, the TIP3P water model, and the CGenFF-derived parameters above for SoD.^15^ Ion–ion and ion–lipid interactions were described in one of two ways: CHARMM^25^ pair-specific Lennard-Jones corrections (SI, Table S4), or the electronic continuum correction (ECC) with a charge-scaling factor of 0.75, following the prosECCo75 protocol.^26^ Fe(ii) non-bonded interactions used Lorentz–Berthelot combining rules. ECC protocols involved the scaling of charges by 0.75 either for all charged species in the system (including POPC lipid headgroups, Cl^−^, K^+^, Fe(ii)) or only to Fe(ii) (SI, Table S18). Fe(ii)-only scaling required the deletion of 3 Cl^−^ ions to neutralise the system.

Temperature was held at 293 K (POPC : cholesterol or DPhPC systems) or 310 K (DDPC, DCPC, DLPC and DMPC systems) using the velocity-rescale thermostat (*τ*_T_ = 1.0 ps), with SoD, membrane and solvent coupled as separate groups. Pressure was held at 1 bar with the Parrinello–Rahman barostat^27^ using semi-isotropic coupling (*τ*_P_ = 5 ps, compressibility 4.5 × 10^−5^ bar^−1^), with reference coordinates for position restraints scaled by COM. The Verlet cutoff scheme used a 1.2 nm cutoff for van der Waals and short-range electrostatics, with van der Waals forces switched off between 1.0 and 1.2 nm, with neighbour lists updated every 20 steps (Verlet buffer tolerance 0.005 kJ mol^−1^ ps^−1^). Long-range electrostatics were treated with particle mesh Ewald^28^ (Fourier grid spacing 0.12 nm, fourth-order interpolation). Bonds to hydrogen were constrained with LINCS^29^ (order 4, one iteration), and the time step was 2 fs (1 fs in initial equilibration steps; Table S11). Linear COM motion was removed every 100 steps separately for the SoD–membrane solvent groups. Energy minimisation used steepest descent until the maximum force fell below 1000 kJ mol^−1^ nm^−1^ or for at most 5000 steps.

### Equilibration and restraints

Systems were equilibrated with a modified version of the CHARMM-GUI six-step NVT/NPT protocol (Table S11), in which the final unrestrained NPT step was extended to 250 ns. The first 50 ns of this step were discarded. Unless otherwise stated, the final 200 ns were used for equilibrium membrane–property analyses. Where SoD COM restraints were used, the COM of the SoD ring heavy atoms (C and N) was restrained relative to the membrane COM (all lipid atoms) by zero-rate COM pulling (0 nm ps^−1^) along the membrane normal (*z*) only, during both equilibration and production. The reference separation was set to its initial value, holding SoD at its starting embedding depth near the bilayer midplane. For simulations performed in the absence or presence of an applied electric field, the force constant was 1000 kJ mol^−1^ nm^−2^ and 2000 kJ mol^−1^ nm^−2^, respectively. Instantaneous restraint forces associated with the applied SoD COM-restraints are reported in Table S13. For the orientation assay, 3 × 1000 ns COM-restrained production runs were initiated from common coordinates of 250 ns equilibrated positionally restrained SoD models starting in either the vertical or horizontal respective orientation.

### Conductance simulations

Conductance simulations were run for 250 ns in NVT under a static uniform electric field (*E*) applied along the membrane normal (*z*) using the GROMACS electric-field-z option. In a periodic system containing a single bilayer, the resulting transmembrane potential difference is Δ*V* = *E*·*L*_*z*_, where *L*_*z*_ is the box length along *z*.^30,31^ Fields were therefore set as *E* = Δ*V*/*L*_*z*_, using *L*_*z*_ from the final frame of each replica's equilibration, to give Δ*V* = −100, −50, +50 or +100 mV. Because the box volume was fixed in NVT, Δ*V* remained constant throughout. Applied fields for each system are listed in Table S12; positive Δ*V* corresponds to a positive applied field (along + *z*), such that the lower (−*z*) side of the membrane is at the higher potential and anions are driven towards −*z*. Each simulation was initiated from the corresponding replica's equilibrated coordinates, with velocities regenerated at the simulation temperature. Conductance was calculated from the net charge carried across the membrane per unit time, counting complete K^+^ and Cl^−^ permeation events (SI, Section S4.2).

### Electropore-mediated insertion

A model electropore was prepared with a two-step protocol similar to that of Fernández *et al.*^32^ First, a 0.5 V nm^−1^ field was applied to a pre-equilibrated 1,2-diphytanoyl-*sn*-glycero-3-phosphocholine (DPhPC) bilayer in NPT until an electropore of suitable size formed. Coordinates were then extracted and SoD was placed in the aqueous phase with an inter-COM distance between SoD and the lipid bilayer of 43.2 Å. System compositions are shown in SI, Table S9J. Simulations were then performed with electropore-stabilising electric fields of ±0.035 V nm^−1^ or with no stabilising field. Ten × 500 ns production repeats were then initiated for each condition from common starting coordinates each with random initial velocities. Definitions of SoD insertion events and corresponding parameters are provided in SI Section S11 and Table S22, respectively.

### Analysis

Trajectory analyses were conducted using custom in-house scripts primarily based on MDAnalysis^33^ and NumPy.^34^ Molecular visualisations were generated using VMD 1.9.4.^35^ Statistical analyses were performed using NumPy and SciPy.^36^

## Results and discussion

### Monomeric SoD stabilises conductive toroidal lipid pores

Building on the previous proposal that SoD embeds horizontally within membranes,^13^ we simulated six independent 250 ns trajectories of monomeric and horizontal SoD ensembles in a 2 : 1 POPC : cholesterol bilayer with COM restraints to maintain stable embedding. Membrane thickness (P–P) stabilised within the first 50 ns with a mean thickness of 46.89 ± 0.09 Å over the final 200 ns across replicas. Simulations revealed hydrated toroidal SoD–lipid pores permeated by chloride ions, with a minimum constriction radius of 2.79 ± 0.08 Å (Fig. 1A and B), calculated using the HOLE algorithm^37^ and consistent with previous estimates between 2.5–3.6 Å.^13^ Further characterisation of these assemblies revealed local membrane deformation and lipid rearrangement around the hydrated SoD–lipid pore, consistent with toroidal pore architectures (Fig. S12 and S13). To assess whether these channels supported ion transport, transmembrane potentials of −100 mV, −50 mV, +50 mV, and +100 mV were then applied along the membrane normal for an additional 250 ns. Current–voltage (*I*–*V*) traces were then constructed, which revealed a mean simulated conductance of 2.05 ± 0.03 nS against a non-SoD containing membrane patch with 0.00 ± 0.00 nS (Fig. 2, panels A–C). Calibration of calculated conductances against experimental and simulated bulk electrolytic conductivity gives a conductance of 1.54 ± 0.02 nS (SI Section S5 and Tables S15–S17).

**Fig. 1:**
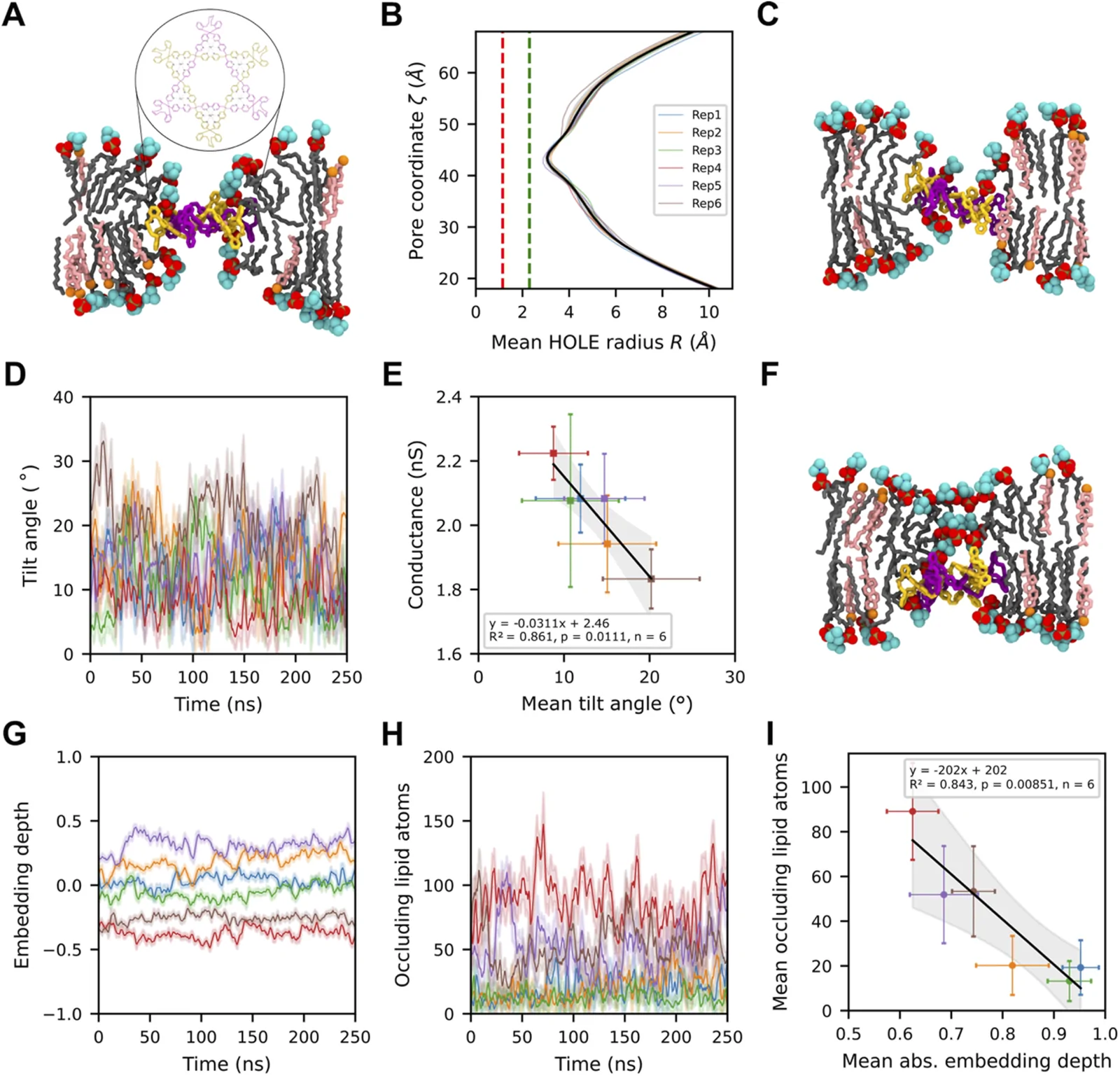
SoD stabilises conductive lipid toroidal pores with conductance associated with membrane-mediated dynamics. (A) Cross-sectional slice through the SoD channel, illustrating lipid rearrangement and toroidal pore formation (rings: yellow/purple licorice; Fe^2+^: pink spheres). POPC headgroups are represented as spheres, with choline carbon atoms shown in cyan, nitrogen atoms in blue, phosphate oxygen atoms in red, and phosphorus atoms in dark yellow. Cholesterol carbon atoms are shown in pink, while cholesterol oxygen atoms are shown in orange. POPC lipid tails and mid-polar heavy atoms are coloured grey. For clarity, some lipid tails have been omitted. (B) Mean HOLE pore radius profiles calculated across the final 200 ns of equilibrated independent 6 × 250 ns simulations of SoD in the presence of COM-restraints. Dashed lines mark the minimum radius for one (red, 1.15 Å) and two (green, 2.30 Å) water molecules. (C) Example resected snapshot of heavy SoD tilting observed in COM-restrained simulations. (D) SoD tilt angles measured across COM-restrained replicas. (E) Linear regression analysis of mean tilt angle against conductance reveals a negative association. The black line indicates ordinary least squares across on replicate means; the shaded region indicates the 95% confidence band; error bars indicate SD of tilt over frames and SEM of conductance across five 50 ns blocks (+100 mV) (F) example resected snapshot of shallow SoD embedding from COM-unrestrained simulations. (G) Normalised SoD embedding depth traces across COM-unrestrained trajectories demonstrate dynamic vertical SoD diffusion. (H) Traces of the number of occluding lipid atoms 250 ns COM-unrestrained simulations. (I) Linear regression analysis of mean absolute embedding depths against mean occluding lipid atoms demonstrates a negative correlation. All quantitative panels share the same replica colour scheme. For simplicity, data presented here belongs only to simulations performed at transmembrane potentials of +100 mV. Additional replica-resolved tilt and embedding depth trajectories are provided in Fig. S6 and S7, respectively. The definition and sensitivity of the lipid occlusion metric are detailed in Fig. S8.

**Fig. 2:**
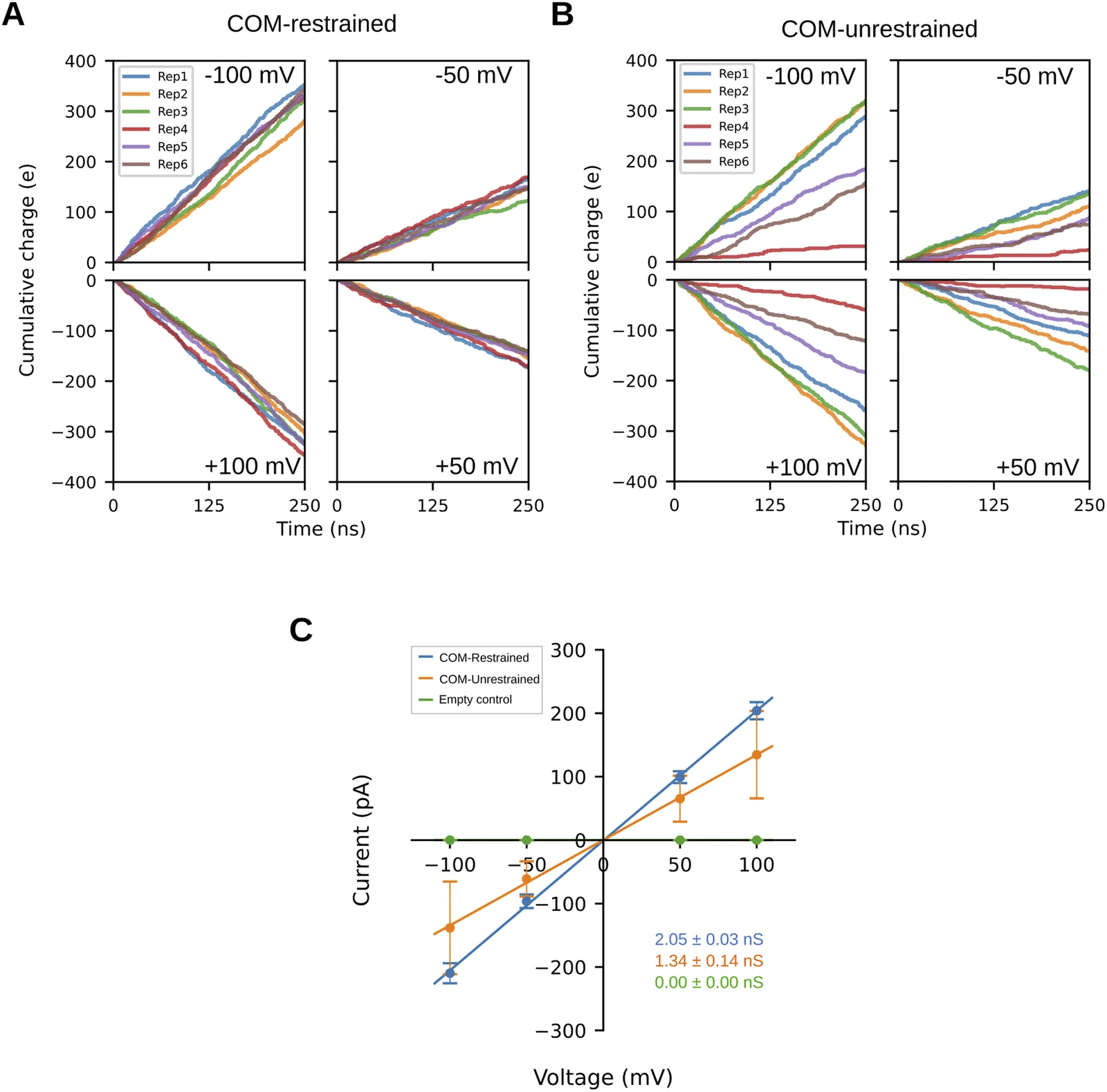
Conductance of COM-restrained and COM-unrestrained SoD models. Conductances were calculated across 250 ns under the application of transmembrane potentials of −100 mV, −50 mV, +50 mV and +100 mV (SI, Table S14A). Cumulative charge-traces for COM-restrained (A) and COM-unrestrained (B) simulations. Current–voltage trace comparing COM-restrained and COM-unrestrained simulations against empty equivalent POPC : cholesterol (2 : 1) membranes in the absence of SoD (C). Conductance was calculated by counting ion crossings across the bilayer (See SI S4.2). Additional analyses of channel stability and membrane perturbation in larger membrane patches are provided in Fig. S14, S15 and Table S20. Relationships between pore stability metrics and conductance across independent replicas are presented in Fig. S23.

Further analysis of chloride coordination during permeation through the SoD channel is shown in SI Section S9 (Fig. S16, S17 and Table S21). In this model, ion conduction emerges from the coupled SoD–membrane system, rather than from the molecular scaffold of SoD alone. Trajectory analysis revealed increased SoD tilting in less conductive simulations (Fig. 1C). Quantification of the angle between the normal vector of the approximate molecular plane of SoD and the membrane normal showed a negative correlation between tilt angle and conductance, with the most conductive states sampled when SoD was most parallel to the bilayer leaflets (Fig. 1D and E). This is consistent with dynamic motions arising from the fluid nature of the phospholipid leaflets bridged by SoD.

To further investigate the embedding stability and dynamics of SoD channels, conductance simulations were repeated in the absence of COM restraints (Fig. 2B and Table S14A). Here, an ensemble of conductive and sub-conductive states was observed, with a mean conductance of 1.34 ± 0.14 nS, (Fig. 2B; SI, Table S14A). Tracking the normalised embedding depth of SoD within the membrane patch across the trajectory highlighted dynamic vertical diffusion of SoD across the bilayer (Fig. 1F and G). Changes in embedding depth were correlated with channel occlusion, quantified as the number of lipid atoms occupying a cylinder of radius 5 Å centred on SoD along the membrane normal (Fig. 1H and I). Increased channel occlusion was associated with reduced conductance, indicating that the most conductive channel states occurred when SoD was positioned closest to the bilayer midplane.

Together, these data demonstrate that SoD dynamically adopts varying positions within the bilayer in these simulations and that the channel conductance was associated with SoD embedding depth. The highest conductance states were observed when SoD occupied the bilayer midplane, similar to the open-state stabilisation behaviour reported for midplane-associated states of SCMTR channels reported by Burkhardt.^12^ These findings support the existence of an ensemble of conductive states for SoD, consistent with the dynamic ensembles of conductive states as proposed for other non-spanning AIC systems by Fyles and Burkhardt.^10,12^

To probe how these membrane deformations are stabilised, we hypothesised that electrostatic interactions between the SoD Fe^2+^ centres and lipid phosphates may stabilise the toroidal assembly. We tested this by scaling charges with the prosECCo75 correction, applied either to all charged species or to the Fe^2+^ ions alone (SI Section S6). Both protocols reduced conductance relative to NBFIX, in both COM-restrained and COM-unrestrained simulations (Fig. 3A; SI Tables S14C and D). This was not due to slower ions, since bulk KCl conductivity was greater under prosECCo75 (SI Table S19). Instead, the pore narrowed: lipid occlusion of the lumen more than doubled and the minimum pore radius fell from 3.1 to about 2.3 Å (Fig. 3B; SI Fig. S10). The narrowing arose from lipid headgroups encroaching on the lumen, while the pore remained hydrated and toroidal (SI, Fig. S10). Scaling the Fe^2+^ charge alone was sufficient to cause this, and it substantially reduced and shortened Fe^2+^–phosphate contacts (SI Fig. S9). Together, these results indicate that Fe^2+^ electrostatics help hold the toroidal pore open, consistent with stabilisation, although charge scaling cannot isolate this interaction as the sole mechanism.

**Fig. 3:**
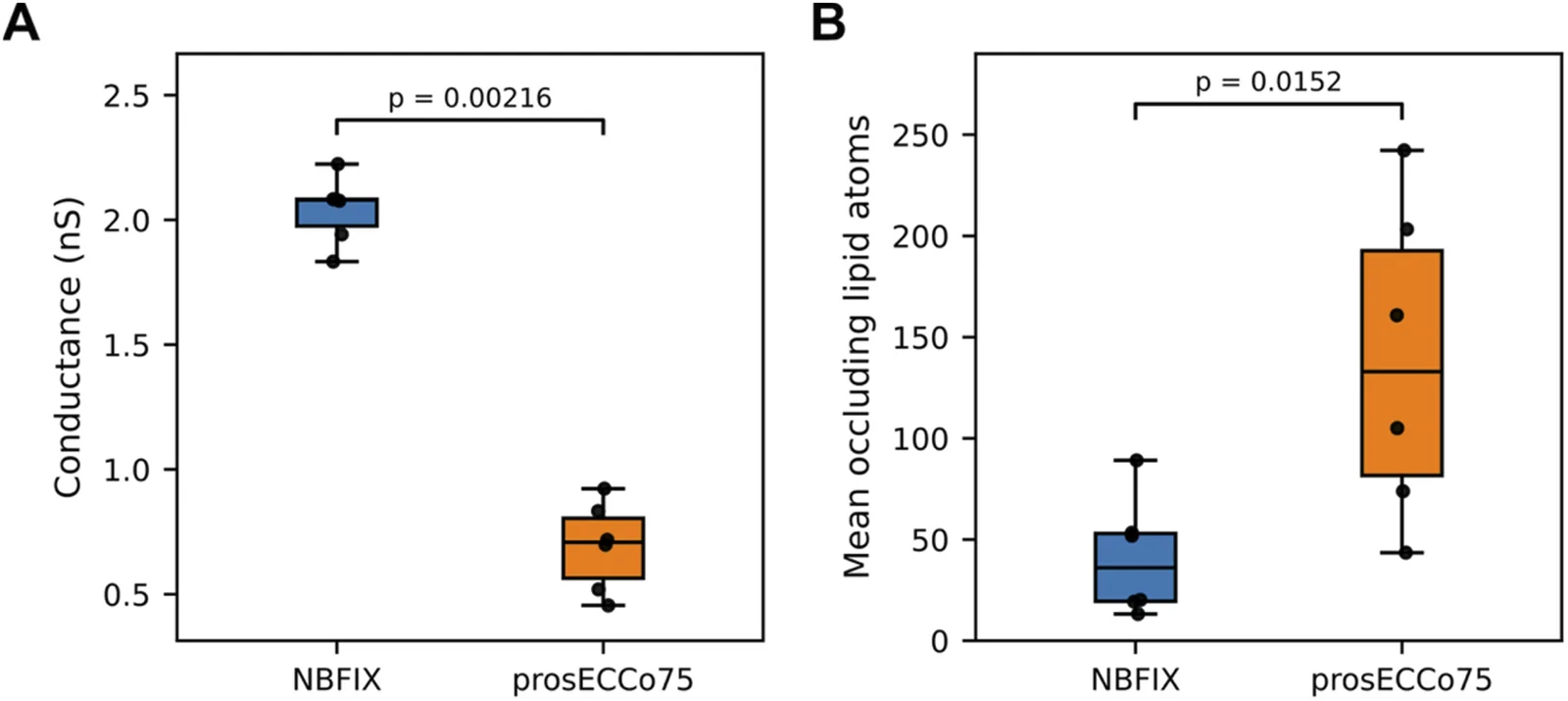
Summary box plots comparing conductance and mean lipid occlusions across charge-scaling methods NBFIX and prosECCo75 (all charges) at +100 mV transmembrane potentials. (A and B) Comparisons between systems treated with either NBFIX or prosECCo75 (all charges) charge handling methods. (A) COM-restrained prosECCo75 simulations exhibited significantly reduced conductances relative to NBFIX (SI, Fig. S9 and Table S14). (B) COM-unrestrained prosECCo75 (all charges) simulations did exhibit significantly increased mean lipid occlusions relative to NBFIX simulations (SI, Fig. S5C and S10D) (B). Boxes, interquartile range; centre line, median; whiskers, range; black symbols, individual replica means.

Toroidal pore formation has previously been observed in natural and synthetic membrane–spanning systems, including biological antimicrobial peptides^38–42^ which promote cytotoxicity and cell death through membrane-disruption, and in synthetic DNA-based AICs,^43,44^ among others. These ensembles typically span the membrane and incorporate hydrophobic membrane–embedding anchors to stabilise toroidal pore architectures. Given the extensive thickness mismatch between SoD and 2 : 1 POPC : cholesterol bilayers, we sought to understand the relationship between membrane thickness and the stability and conductance of SoD–lipid channels. SoD was simulated within a series of pure saturated phosphatidylcholine (PC) bilayers with varying membrane thicknesses (SI Fig. S18), namely: 1,2-didecanoyl-*sn*-glycero-3-phosphocholine (DDPC, 10 : 0/10 : 0) (28.68 ± 0.02 Å), 1,2-diundecanoyl-*sn*-glycero-3-phosphocholine (DCPC, 11 : 0/11 : 0) (31.05 ± 0.02 Å), 1,2-didodecanoyl-*sn*-glycero-3-phosphocholine (DLPC, 12 : 0/12 : 0) (33.61 ± 0.05 Å), and 1,2-dimyristoyl-*sn*-glycero-3-phosphocholine (DMPC, 14 : 0/14 : 0) (39.42 ± 0.09 Å) (Fig. 4A). Simulations were performed at 310 K to allow sufficient headspace above the main phase-transition temperature (*T*_m_) of DMPC (297.05 K).^45^ SoD conductance decreased with increasing membrane thickness in both COM-restrained and COM-unrestrained simulations, in the order DMPC (COM-restrained 2.95 ± 0.03 nS; COM-unrestrained 3.06 ± 0.06 nS, *I*–*V* conductance ± SE) < DLPC (3.46 ± 0.08; 3.42 ± 0.04 nS) < DCPC (3.79 ± 0.05; 3.78 ± 0.06 nS) < DDPC (3.86 ± 0.04; 3.96 ± 0.05 nS) (Fig. 4B and C). Importantly, COM-restrained and COM-unrestrained conductances agreed more closely in the saturated PC bilayers than in 2 : 1 POPC : cholesterol bilayers. In the PC bilayers, the unrestrained-to-restrained *I*–*V* conductance ratio was 0.99–1.04. In POPC : cholesterol it was 0.66. This suggests that SoD–lipid pores are more stable in the saturated PC membranes at 310 K than in POPC : cholesterol at 293 K, although lipid composition and temperature differ between these systems. Nevertheless, SoD remained conductive in POPC : cholesterol even without COM restraints, albeit with greater variability between replicas, showing that these pre-embedded architectures can remain stable and conductive under the simulated conditions.

**Fig. 4:**
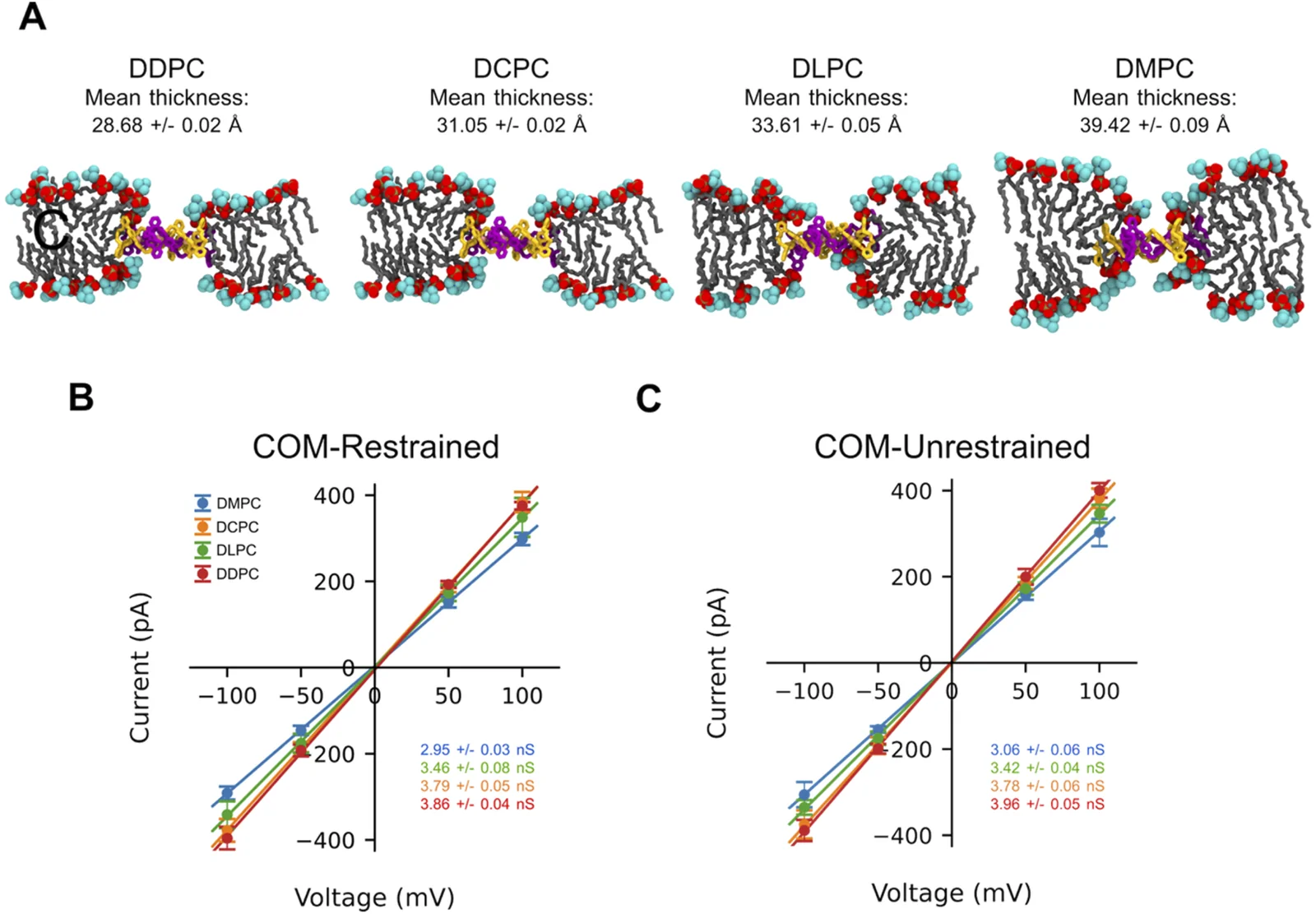
SoD–lipid channel behaviours across saturated phosphatidylcholine membranes of varying thicknesses. (A) Resected molecular images of COM-restrained SoD within an DDPC, DCPC, DLPC and DMPC. *I*–*V* traces were calculated for COM-restrained (B) and COM-unrestrained (C) simulations across each membrane type. Additional comparisons of SoD tilt and embedding depth across membrane compositions and force-field treatments are provided in Fig. S24.

### Investigating alternative membrane–spanning SoD configurations

Given the dimensions of SoD (31 Å diameter; 15.5 Å thickness),^13^ conductive channel assemblies could plausibly arise from vertically oriented monomers and/or from stacked horizontally embedded dimers capable of spanning the bilayer. To evaluate these alternative membrane–spanning models, we performed comparative MD simulations of each configuration within 2 : 1 POPC : cholesterol bilayers.

Three simulations of initially vertically oriented SoD monomers were performed for 1 µs with COM restraints to maintain membrane embedding. Within 500 ns, these assemblies reoriented into horizontally embedded configurations, indicating that initially constructed vertically orientated states are unstable under these simulation conditions and can reorient to horizontally embedded configurations (Fig. 5A).

**Fig. 5:**
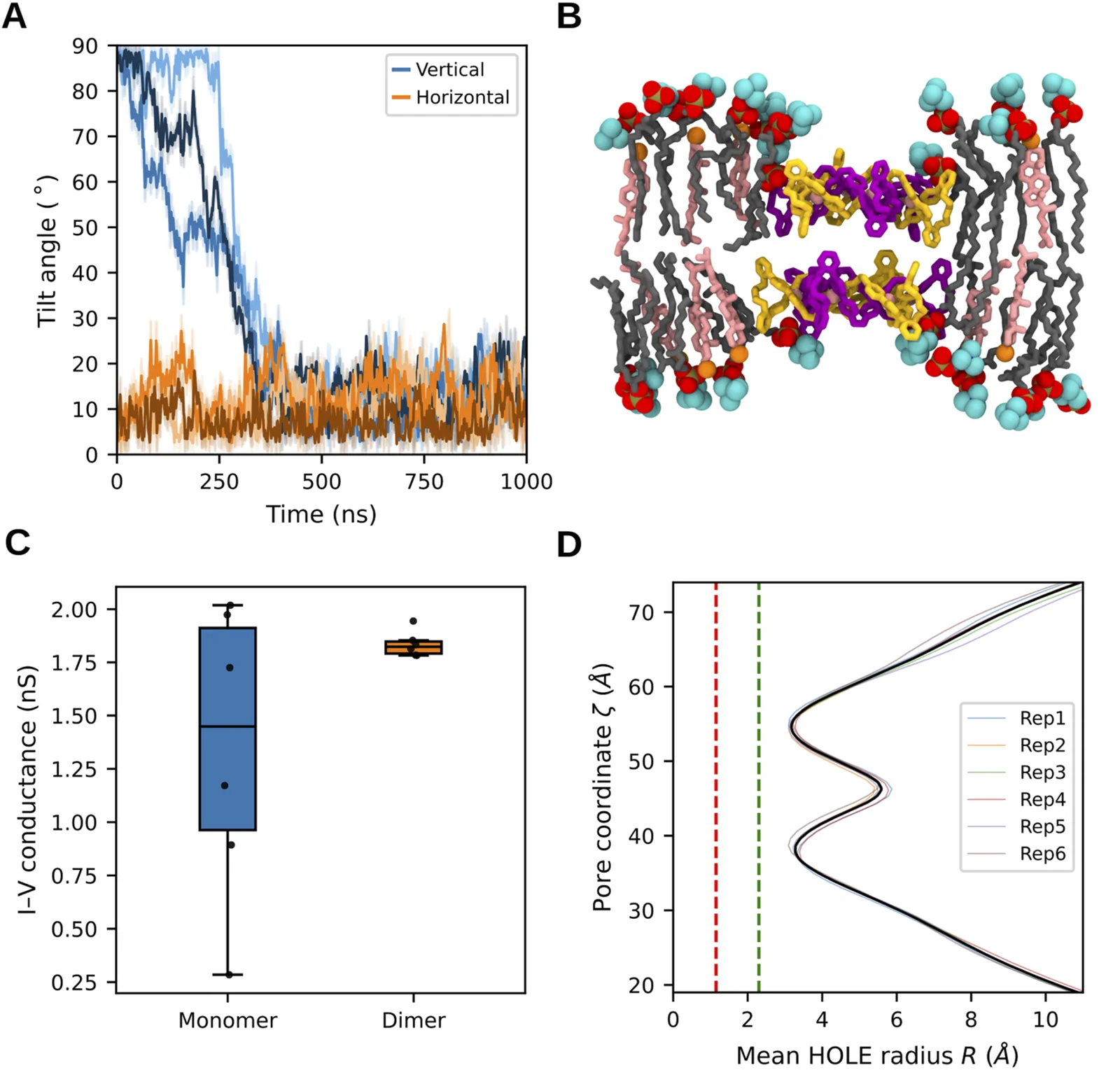
Investigating alternative, membrane–spanning models of SoD channels. (A) Tilt angle traces of simulations with COM-restrained SoD in either initially vertical (blue) or horizontal (orange) demonstrate that SoD ensembles configurations show reorientation of initially vertical SoD towards horizontal configurations within POPC : cholesterol (2 : 1) bilayers. (B) Cross-sectional resected slice across a representative dimeric SoD channel. (C) Comparison of mean conductances between COM-unrestrained monomeric and dimeric SoD systems. Boxes, interquartile range; centre line, median; whiskers, range; black symbols, individual replica means. (D) Mean HOLE profiles of the dimeric replicas highlight dual constriction sites formed by both SoD molecules. Dashed lines mark the minimum radius for one (red, 1.15 Å) and two (green, 2.30 Å) water molecules.

Dimeric SoD assemblies were simulated using protocols otherwise identical to those for the monomer, but without COM-restraints. Because the dimer spans the bilayer, with one SoD in each leaflet, restraints were not needed to keep it at the midplane, and restraining each SoD individually would instead have fixed their separation at the starting value. Simulated dimers (Fig. 5B) formed hydrated chloride-permeable channels with a mean conductance of 1.84 ± 0.02 nS (SI Table S14N). This COM-unrestrained dimeric system was stable (SI, Fig. S11) and had a greater mean conductance compared to the equivalent COM-unrestrained monomeric system simulated under the same protocol (1.34 ± 0.14 nS), though this difference was not statistically significant (permutation test, *p* = 0.15) (Fig. 5C). Unlike the monomer, the membrane–spanning dimer remained stably embedded at the bilayer midplane (Fig. 1G, SI Fig. S11C), so we also compared it with the COM-restrained midplane-associated monomeric system (2.05 ± 0.03 nS), which revealed that the conductance of the dimeric system was significantly lower (permutation test, *p* = 0.004) (Fig. 5C). The observed difference in conductance against COM-restrained monomeric assemblies is consistent with the presence of two constriction sites in the dimeric system (Fig. 5D).

### SoD can insert through a pre-formed membrane defect in simulations

Finally, we investigated how the proposed channel architecture may arise. Given the symmetric distribution of the +12 charge across SoD, partitioning into an intact lipid bilayer would be expected to be highly energetically unfavourable. We therefore hypothesised that SoD may instead reach the bilayer centre *via* pre-existing defects, such as the transient nano-scale lipid toroidal pores observed during electroporation.^46,47^

Simulations with SoD initially positioned beneath an electropore revealed a stepwise insertion pathway (Fig. 6). SoD first adsorbed at the membrane surface adjacent to the pore opening in an upright position, then reoriented to span the opening, and finally moved towards the bilayer core, adopting a predominantly horizontal orientation which resembled the conductive pre-embedded models (SI, Fig. S22). Insertion occurred in 6/10 and 7/10 replicas under transmembrane fields of +0.035 and −0.035 V nm^−1^, respectively, but in none of the 10 replicates without an applied field, where SoD remained adsorbed outside the phosphate plane (Fisher's exact test, *p* = 0.001; SI, S11.2). These results suggest that electropores provide a controllable model of transient membrane defects that can support SoD insertion into conductive configurations.

**Fig. 6:**
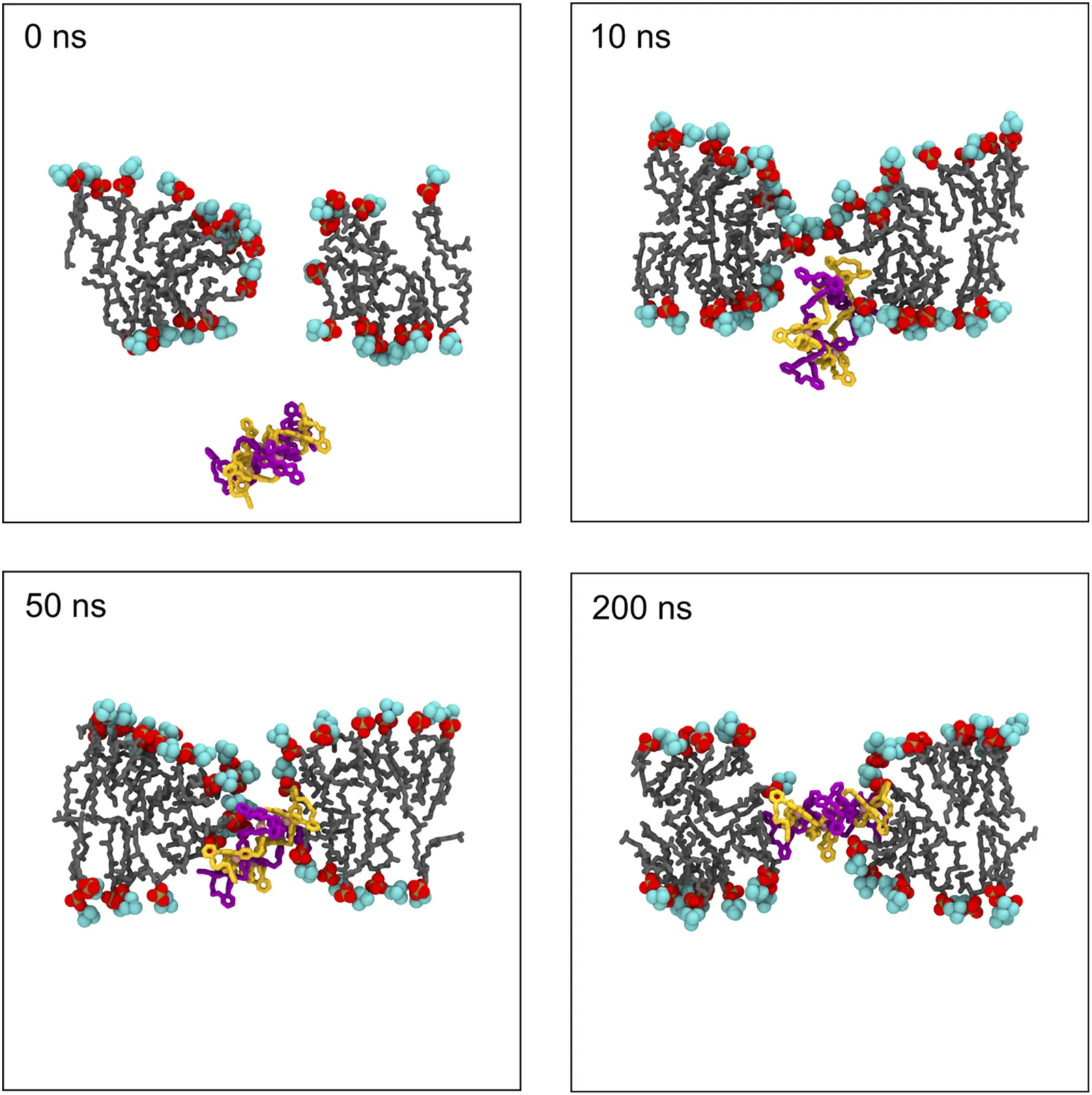
SoD insertion into DPhPC bilayers *via* electropores to reach conductive configurations. Representative resected snapshots illustrate key insertion events observed in the simulations. Replica-resolved insertion trajectories under positive, negative and zero applied electric fields are provided in Fig. S19–S21, respectively.

Importantly, these simulations represent a forced pathway: the electropore was generated by a strong electric field and maintained by an applied field, and no insertion occurred in the absence of that field. SoD channels were experimentally observed in lipid vesicles in the absence of externally applied voltages.^13^ We therefore do not propose electroporation as the only insertion mechanism, but rather as one possible route.

## Conclusion

Our simulations support a model in which pre-embedded monomeric SoD stabilises a hydrated, lipid-lined toroidal pore that supports chloride conduction despite SoD not itself spanning the bilayer. In this model, the membrane forms an integral part of the conductive pathway, with SoD–lipid interactions and membrane dynamics associated with changes in channel conductance. Simulations with pre-formed membrane defects further suggest one possible route to the embedded state, though other potential routes or indeed the final population of states cannot be ruled out at this stage. Together with previous experimental observations, these findings support a molecularly detailed model for SoD conduction in which membrane deformation enables a conductive pathway without SoD itself spanning the bilayer.

## Conflicts of interest

There are no conflicts to declare.

## Supplementary Material

RA-OLF-D6RA06238E-s001

## Data Availability

Reference simulation data has been deposited on MDposit, https://mdposit.mddbr.eu, under project accession codes MD-A00C4F to MD-A00C4Y. Supplementary information (SI): force-field validation, simulation details, conductance data, analysis methods, control simulations, and further characterisation of SoD behaviour and membrane deformation. See DOI: https://doi.org/10.1039/d6ra06238e.
