# Supplementary material for: Membrane deformation supports ion conduction by the star of David [2]catenane
Source: RSC Adv. 2026 Nov 3. Online ahead of print. doi: 10.1039/d6ra06238e (PMC13730457; doi:10.1039/d6ra06238e)
Supplement: Supplementary file 1 [file RA-OLF-D6RA06238E-s001.pdf]

Supplementary Information (SI) for RSC Advances.
This journal is © The Royal Society of Chemistry

Supporting Information

Membrane Deformation Supports Ion Conduction by the Star of David [2]Catenane

Eden J. Gage and Philip C. Biggin*

Structural Bioinformatics and Computational Biochemistry, Department of Biochemistry, South Parks Road, Oxford. OX1 3QU.

*Correspondence: Philip.biggin@bioch.ox.ac.uk

Table of Contents

LIST OF TABLES. **4**

LIST OF FIGURES. **5**

S1. PARAMETERIZATION AND VALIDATION OF THE SOD FORCE FIELD **6**

S1.1 Force field system overview, derivation and atom typing 6
S1.2 Preservation of the experimental coordination geometry 13

S2. SIMULATION MODELS AND PROTOCOLS **22**

S2.1 Model compositions 22
Table S9. Composition and dimensions of each simulation system. 22
S2.2 Simulation protocols 30

S3 DATA **42**

S3.1 Conductance data 42
S3.2 Occluding Lipid Atoms 74
S3.3 SoD Tilt 75
S3.4 SoD Embedding Depth 76

S4 EXPLANATION AND SENSITIVITY TESTS OF ANALYSIS TOOLS **77**

S4.1 Radial exclusion radius for membrane boundaries 77
S4.2 Ion Permeation and Single-Channel Conductance Calculations 78
S4.3 SoD Tilt Angle Analysis 80
S4.4 SoD Embedding Depth Analysis 81
S4.5 Channel Lipid Occlusion Analysis 82
S.4.5.1 Analysis overview *82*
S4.5.1 Sensitivity to cylinder radius *82*
S4.6 Bulk Electrolyte Conductivity 85
S4.7 HOLE 85
S4.8 Leaflet curvature 85
S4.9 Membrane Thickness 86
S4.10 Lipid Headgroup Orientation 86

S5 CALIBRATION OF SIMULATED CONDUCTANCES AGAINST BULK ELECTROLYTE CONDUCTIVITY . **87**

S5.1 Simulated electrolytic conductivity 87
S5.2 Experimental reference 87
S5.3 Calibration 87

S6 PROSECCO75 .... **90**

S6.1 Simulation details 90
S6.2 Bulk 1 M KCl conductivity 91
S6.3 Fe(II)-phosphate interactions 92

S7 STABILITY OF DIMERIC SOD ASSEMBLIES **97**

S8 FURTHER CHARACTERISATION OF MEMBRANE DEFORMATIONS AND SOD-LIPID CHANNELS AND SYSTEM SIZE VALIDATION **99**

S8.1 Membrane-Wide Deformation 100
S8.2 Channel Properties 102

S8.3 Finite Size Effects 103
S8.3.1 Periodic-image separation *103*
S8.3.2 SoD-Lipid Pore Membrane Deformation *103*
S8.3.3 Conductance and Stability of SoD in 150 x 150 Å Patches *105*

S9 FURTHER CHARACTERISATION OF SOD-LIPID CHANNEL CHLORIDE PERMEATION MECHANISMS 107

S9.1 Definition of first-shell contacts and Cl- permeation routes 107
S9.2 Chloride permeation across SoD-lipid channels 109

S10 FURTHER DETAILS OF PHOSPHATIDYLCHOLINE MEMBRANE THICKNESS ASSAY 111

S11. ELECTROPORE-MEDIATED SOD INSERTION 112

S11.1. Definitions and contact traces 112
S11.2 Bulk bilayer thickness and definition of insertion 116

S12. ADDITIONAL STATISTICAL ANALYSIS 118

REFERENCES 120

List of Tables.

Table S1. CGenFF atom types used in the LIG macrocycle. 7
Table S2. Lennard-Jones parameters. 8
Table S3. Bonded CGenFF parameters assigned by analogy with non-zero penalty scores. 10
Table S4. Pair-specific Lennard-Jones (NBFIX) overrides applied in addition to Lorentz-Berthelot combining rules. 11
Table S5. Fe(II)-N distance restraints. 12
Table S6. Solvated SoD simulation model compositions. 13
Table S7. Simulation protocol for solvated SoD simulations. 15
Table S8. Mean Fe(II) coordination geometric descriptors. 17
Table S9. Composition and dimensions of each simulation system. 22
Table S10. Initial and final simulation box dimensions. 24
Table S11. Minimisation, equilibration and production protocols. 30
Table S12. Applied electric field strengths for each system. 31
Table S13. Instantaneous restraint forces for COM-restrained simulations. 40
Table S14. Conductance data for all simulations. 42
Table S15. Bulk electrolyte conductivities for NBFIX and prosECCo75 models. 88
Table S16. Charge-scaling simulated-experimental conductivity correction factors. 88
Table S17. Raw and calibrated simulated conductances. 89
Table S18. prosECCo75 charge-scaled atoms. 90
Table S19. Calculated bulk electrolyte conductivity values. 91
Table S20. Membrane perturbation in POPC:cholesterol (2:1) bilayers of two patch sizes. .. 104
Table S21. RDF first peak and minima (contact distances) for interactions used to derive contact cutoff distances. 108
Table S22. Membrane thickness parameters for ± 0.035 V nm^{-1} simulations. 115

List of Figures.

Figure S1. Force field parameterization and coordinate fidelity for the SoD macrocycle......... 9
Figure 2. Validation of the SoD force-field parameterisation and Fe–N harmonic bond restraint treatment. 14
Figure S3. 2,2'-bipyridine N1-C2-C2'-N1' torsional scan profiles for QM (r2-Scan-3c) and MM (CGenFF). 18
Figure 4. Effect of N1–C2–C2′–N1′ dihedral restraints on SoD structure and Cl⁻ binding. 20
Figure 5. SoD channel occluding lipid atoms. 74
Figure 6. SoD tilt angle traces. 75
Figure S7. SoD embedding depths. 76
Figure 8. Lipid occlusion metric and sensitivity to cylinder radius. 84
Figure S9. Fe(II)-lipid interactions, bulk conductivity and pore conductance under each charge-scaling model. 93
Figure S10. Pore constriction under charge scaling. 95
Figure S11. Structural stability of dimeric SoD assemblies. 97
Figure S12. Membrane deformations observed within SoD-lipid pore containing systems. 100
Figure 13. Configuration of solvated SoD-lipid toroidal pores. 102
Figure 14. Conductances of SoD in 150 x 150 Å membranes. 105
Figure 15. Comparison of channel stability metric means between SoD systems with 85 x 85 Å or 150 x 150 Å membrane patches. 106
Figure S16. Cl^- - RDFs with water, K+, POPC choline, cholesterol and SoD hydrogens, used to define contact distances. 108
Figure S17. Insights into chloride permeation through SoD centre for COM-restrained SoD under +100 mV transmembrane voltages. 109
Figure 18. Phosphatidylcholine membrane thickness assay composition. 111
Figure S19. Insertion metric traces for SoD – electropore simulations in the presence of a +0.035 V nm^{-1} stabilising electric field. 113
Figure S20. Insertion metric traces for SoD – electropore simulations in the presence of a -0.035 V nm^{-1} stabilising electric field. 114
Figure S21. Insertion metric traces for SoD – electropore simulations in the absence of a stabilising electric field. 115
Figure 22. SoD – electropore insertion simulations. 116
Figure 23. Relationships between pore stability metrics and conductance across replicates. 118
Figure 24. Summary box plots comparing SoD tilt and embedding depth across force-field and membrane-composition conditions. 119

S1. Parameterization and validation of the SoD force field

S1.1 Force field system overview, derivation and atom typing

The deposited fitted crystallographic structure of the Star of David [2]catenane (SoD) was obtained from the Cambridge Crystallographic Data Centre (CCDC) (ID: 992376)[1]. SoD consists of two identical macrocyclic ligands (LIG, 306 atoms each, net neutral) and six Fe(II) ions (FE2P, +2e each), equating to a total charge of +12. The crystallographic model was pre-processed to remove any non-SoD atoms. The Fe(II) atom (FE2P) and its Lennard-Jones parameters are from the standard CHARMM ions divalent parameter set[2]. All organic parameters (atom types, charges, bonded terms) were assigned by the CHARMM General Force Field (CGenFF) program v 2.5.1 accessed by CHARMM-GUI's Ligand Reader and Modeler by analogy to existing CHARMM36/CGenFF atom types (**Table S1**)[3-5]. **Table S2** shows the CHARMM36/CGenFF Lennard-Jones parameters (σ, ε; Lorentz-Berthelot combining rules unless overridden) for every atom type appearing in LIG and FE2P. Penalty scores from the CGenFF parameterization for the LIG topology are: maximum penalties for bonded parameters and charges are 56.6 and 3.185, respectively. Per CGenFF guidance, penalties below 10 indicate the analogy used is fair; 10-50 warrants basic validation; above 50 indicates poor analogy requiring extensive validation. All individual atomic charge penalties are ≤ 3.185, within the range CGenFF considers a fair analogy. Bonded penalties are more variable, but the two highest-penalty dihedrals (56.6 and 30) both have CG2R61-CG2R67 as their central bond, an intra-ring aromatic bond whose torsion is fixed by ring closure and which therefore contributes negligibly to conformational sampling; the 56.6 term is assigned from a fluorinated aromatic analogue (FGR1-CG2R66-CG2R67-CG2R67), which accounts for the elevated score despite the comparable bonding situation. The only high-penalty terms about freely rotatable bonds (25 and 22) are centered on CG321-CG321 bonds within the aliphatic linker and ethyl bridges, at least three bonds from any coordinating nitrogen. No parameter defining the bipyridine geometry or the Fe(II) coordination environment carries a penalty above 0.5 (**Fig. S1A, Table S3**). Non-zero bonded CGenFF penalty scores are shown in **Table S3**.

Hydrogen coordinates were not transferred from the deposited model owing to atom-name mismatch and were rebuilt from internal-coordinate tables; both rings were independently briefly minimized (50 steps steepest descent, restrained then unrestrained) during CHARMM-GUI processing. This relieved chemically unreasonable bond lengths in the 1,2-diphenyl-hex-3-ene linkers of the deposited model, with alkene C=C distances of 1.180, 1.727 and 1.743 Å relaxing to 1.315-1.346 Å, and sp^3-sp^3 C-C distances of 1.632-1.666 Å relaxing to 1.538-1.557 Å, while leaving the remainder of the assembly essentially unchanged (LIG heavy-atom RMSD 0.137 Å; Fe(II) centres 0.007 Å; **Fig. S1B**).

Within the crystallographic model, Fe(II) is coordinated by three bidentate 2,2'-bipyridine units, resulting in a six-coordinate FeN_6 environment with an approximately octahedral geometry. Each bipyridine ligand chelates the Fe(II) ion through two pyridyl nitrogen donors. As Fe(II)-N coordination is not represented by classical bonded terms in the CHARMM force field, the octahedral Fe(II)-N coordination spheres were maintained close to their crystallographic geometries using GROMACS harmonic distance restraints (type 6). Equilibrium distances were measured for each Fe-N pair directly from the CCDC crystal structure using MDAnalysis[6]. A force constant of k = 226,103.36 kJ.mol^{-1} nm^{-2} was applied, equivalent to the 270.2 kcal.mol^{-1} Å^{-2} Fe-N bond force constant employed in the CHARMM36 heme parameter set for Fe-NPH[7]. This gives 6 Fe(II) centres x 6 restrained Fe-N pairs = 36 restrained pairs in total, listed in **Table S5**.

Explicit pairwise Lennard-Jones corrections applied to FE2P are listed in Table S4; these cover aliphatic carbon and carbonyl/ester oxygen types inherited from the CHARMM ion parameter set. No pair-specific correction is available for Fe(II) with aromatic nitrogen, chloride, or phosphate oxygen, and these interactions are therefore described by standard Lorentz-Berthelot combining rules.

Table S1. CGenFF atom types used in the LIG macrocycle.

Atom type	Count (per LIG)	Charge range (e)	Chemical environment
CG2R61	96	-0.118 to +0.180	Aromatic ring carbon (unsubstituted/substituted)
CG2R67	30	0.000 to +0.301	Aromatic ring-fusion carbon
NG2R60	18	-0.601 to -0.573	Pyridine/pyrrole-type aromatic N (Fe-coordinating)
CG321	24	-0.192 to -0.171	Aliphatic linker CH_2
CG2D1	6	-0.156	Alkene CH
HGA2	48	0.090	Aliphatic H (CH_2)
HGA4	6	0.150	Alkene H
HGR61	60	0.115	Aromatic ring H
HGR62	18	0.116 to 0.117	Aromatic ring H (adjacent to N)

Table S2. Lennard-Jones parameters.

Type	At. no.	σ (nm)	ε (kJ mol^{-1})
FE2P	26	0.192452	0.667348
CG2R61	6	0.355005	0.292880
CG2R67	6	0.355005	0.292880
CG321	6	0.358141	0.234304
CG2D1	6	0.372396	0.284512
NG2R60	7	0.336760	0.251040
HGA2	1	0.238761	0.146440
HGA4	1	0.222725	0.129704
HGR61	1	0.242004	0.125520
HGR62	1	0.195998	0.192464

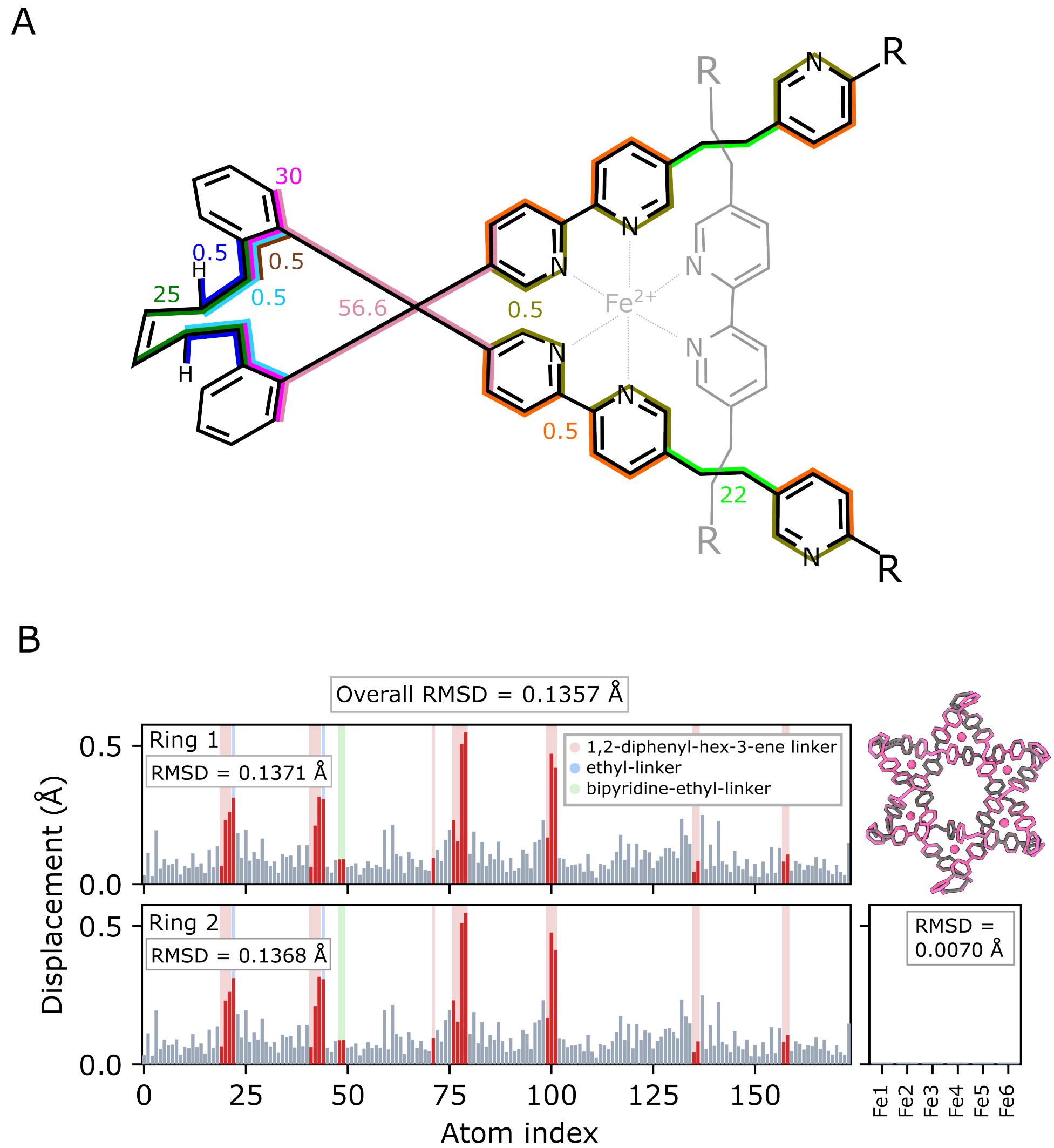

Figure S1. Force field parameterization and coordinate fidelity for the SoD macrocycle.

(A) Non-zero CGenFF bonded-parameter penalty scores mapped onto the ligand structure with colouring as follows: Angle - CG2R67-CG2R61-CG321 (brown); Dihedrals - CG2R67-CG2R61-CG2R61-CG2R67 (orange), CG321-CG2R61-CG2R67-CG2R61 (pink), CG321-CG2R61-CG2R67-CG2R67 (red), CG2R67-CG2R61-CG321-CG321 (cyan), CG2R67-CG2R61-CG321-HGA2 (dark blue), CG2R67-CG2R61-NG2R60-CG2R67 (dark yellow), CG2D1-CG321-CG321-CG2R61 (dark green), CG2R61-CG321-CG321-CG2R61 (light green). **(B)** Per-atom heavy-atom displacement between the deposited crystallographic coordinates (CCDC 992376) and the CHARMM-GUI Ligand Reader and Modeler output, for each macrocycle of the catenane (left) and the six Fe(II) centres (lower right). The crystal (grey) and CHARMM-GUI output (pink) are shown on the upper right and demonstrate minimal coordinate changes outside of linker regions.

Table S3. Bonded CGenFF parameters assigned by analogy with non-zero penalty scores.

Term		Assigned by analogy from	Penalty
Angle	CG2R67-CG2R61-CG321	CG2R61-CG2R61-CG321	0.5
Dihedral	G2R67-CG2R61-CG2R61-CG2R67	CG2R61-CG2R61-CG2R61-CG2R67	0.5
	CG321-CG2R61-CG2R67-CG2R61	CG2R61-CG2R61-CG2R61-CG321	30
	CG321-CG2R61-CG2R67-CG2R67	FGR1-CG2R66-CG2R67-CG2R67	56.6
	CG2R67-CG2R61-CG321-CG321	CG2R61-CG2R61-CG321-CG321	0.5
	CG2R67-CG2R61-CG321-HGA2	CG2R61-CG2R61-CG321-HGA2	0.5
	CG2R67-CG2R61-NG2R60-CG2R67	CG2R67-CG2R61-NG2R60-CG2R61	0.5
	CG2D1-CG321-CG321-CG2R61	CG2D1-CG321-CG321-CG2D1	22
	CG2R61-CG321-CG321-CG2R61	CG2R53-CG311-CG321-CG2R51	25

Table S4. Pair-specific Lennard-Jones (NBFIX) overrides applied in addition to Lorentz-Berthelot combining rules.

Atom type i	Atom type j	σ (nm)	ε (kJ mol^{-1})	Interaction	Source
CLA	POT	0.36358	0.4780	Cl^{-} - K^{+}	Savelyev and Mackerell, JPCB 2015
CRL1	CTL1	0.35725	0.1151	Sterol ring C-lipid aliphatic C	
CRL1	HAL1	0.29845	0.1226	Sterol ring C-lipid aliphatic H	
CRL1	HAL2	0.29845	0.1343	Sterol ring C-lipid aliphatic H	
CRL2	CTL1	0.35814	0.1485	Sterol ring C-lipid aliphatic C	
CRL2	HAL1	0.29934	0.1586	Sterol ring C -lipid aliphatic H	
CRL2	HAL2	0.29934	0.1736	Sterol ring C-lipid aliphatic H	Lim et al. 2012
HGA1	CTL1	0.29756	0.1289	Aliphatic H-lipid aliphatic C	
HGA1	HAL1	0.23876	0.1377	Aliphatic H-lipid aliphatic H	
HGA1	HAL2	0.23876	0.1502	Aliphatic H-lipid aliphatic H	
HGA2	CTL1	0.29756	0.1134	Aliphatic H-lipid aliphatic C	
HGA2	HAL1	0.23876	0.1209	Aliphatic H-lipid aliphatic H	
HGA2	HAL2	0.23876	0.1326	Aliphatic H- lipid aliphatic H	

Values are from the CHARMM36 (July 2022) nbfix.itp; σ and ε are rounded from the full-precision values in the topology.

Table S5. Fe(II)-N distance restraints.

Fe centre	Coordinating N (ligand copy, atom)	r_0 (nm)	k (kJ.mol^{-1}.nm^{-2})
Fe1	1LIG N5	0.19734	226103.36
Fe1	1LIG N6	0.19786	226103.36
Fe1	2LIG N13	0.19777	226103.36
Fe1	2LIG N14	0.19355	226103.36
Fe1	2LIG N15	0.19414	226103.36
Fe1	2LIG N16	0.19527	226103.36
Fe2	1LIG N13	0.19788	226103.36
Fe2	1LIG N14	0.19355	226103.36
Fe2	1LIG N15	0.19414	226103.36
Fe2	1LIG N16	0.19531	226103.36
Fe2	2LIG N5	0.19734	226103.36
Fe2	2LIG N6	0.19778	226103.36
Fe3	1LIG N1	0.19463	226103.36
Fe3	1LIG N2	0.19464	226103.36
Fe3	1LIG N3	0.19802	226103.36
Fe3	1LIG N4	0.19583	226103.36
Fe3	2LIG N11	0.19693	226103.36
Fe3	2LIG N12	0.19722	226103.36
Fe4	1LIG N11	0.19693	226103.36
Fe4	1LIG N12	0.19725	226103.36
Fe4	2LIG N1	0.19468	226103.36
Fe4	2LIG N2	0.19470	226103.36
Fe4	2LIG N3	0.19797	226103.36
Fe4	2LIG N4	0.19583	226103.36
Fe5	1LIG N17	0.19882	226103.36
Fe5	1LIG N18	0.19690	226103.36
Fe5	2LIG N7	0.19954	226103.36
Fe5	2LIG N8	0.19644	226103.36
Fe5	2LIG N9	0.19774	226103.36
Fe5	2LIG N10	0.19925	226103.36
Fe6	1LIG N7	0.19954	226103.36
Fe6	1LIG N8	0.19648	226103.36
Fe6	1LIG N9	0.19765	226103.36
Fe6	1LIG N10	0.19923	226103.36
Fe6	2LIG N17	0.19882	226103.36
Fe6	2LIG N18	0.19679	226103.36

S1.2 Preservation of the experimental coordination geometry

To assess the extent to which the force fields and the Fe-N restraint-based simulation method maintain the crystallographic Fe(II) coordination environment following equilibration, SoD was simulated in a chloride-neutralised water box and was geometrically compared against the CHARMM-GUI optimised crystal structure and against geometrically optimised Fe(II)-tris-2,2'-bipyridine-5,5'-dimethyl complexes prepared using quantum mechanics (QM).

CHARMM-GUI Solution Builder was used to prepare 6 independent replicates of solvated SoD within a 100 x 100 x 100 Å simulation box neutralized with 12 Cl^- counterions (**Table S6)**. Replicates were energetically minimised, equilibrated and run in production according to the protocol described in **Table S7** with the same GROMACS parameters previously described within this study (see Methods). Equilibrated models (example shown in **Fig. S2A**) were simulated in production for 250 ns giving a total simulation time of 1.5 µs. Post-processing involved making molecules whole and centering and compacting the box on SoD using GROMACS gmx trjconv.

Table S6. Solvated SoD simulation model compositions.

Replica	Components				Total atoms	Box length (cube) (Å)		
	LIG	FE2P	CLA	TIP3		Starting model	Production input	Mean production
1	2	6	12	31572	95346	100	97.9521	97.933 ± 0.052
2	2	6	12	31577	95361	100	97.9206	97.940 ± 0.051
3	2	6	12	31577	95361	100	97.9450	97.940 ± 0.052
4	2	6	12	31576	95358	100	97.9218	97.938 ± 0.052
5	2	6	12	31571	95343	100	97.9194	97.934 ± 0.051
6	2	6	12	31579	95367	100	97.8864	97.941 ± 0.052

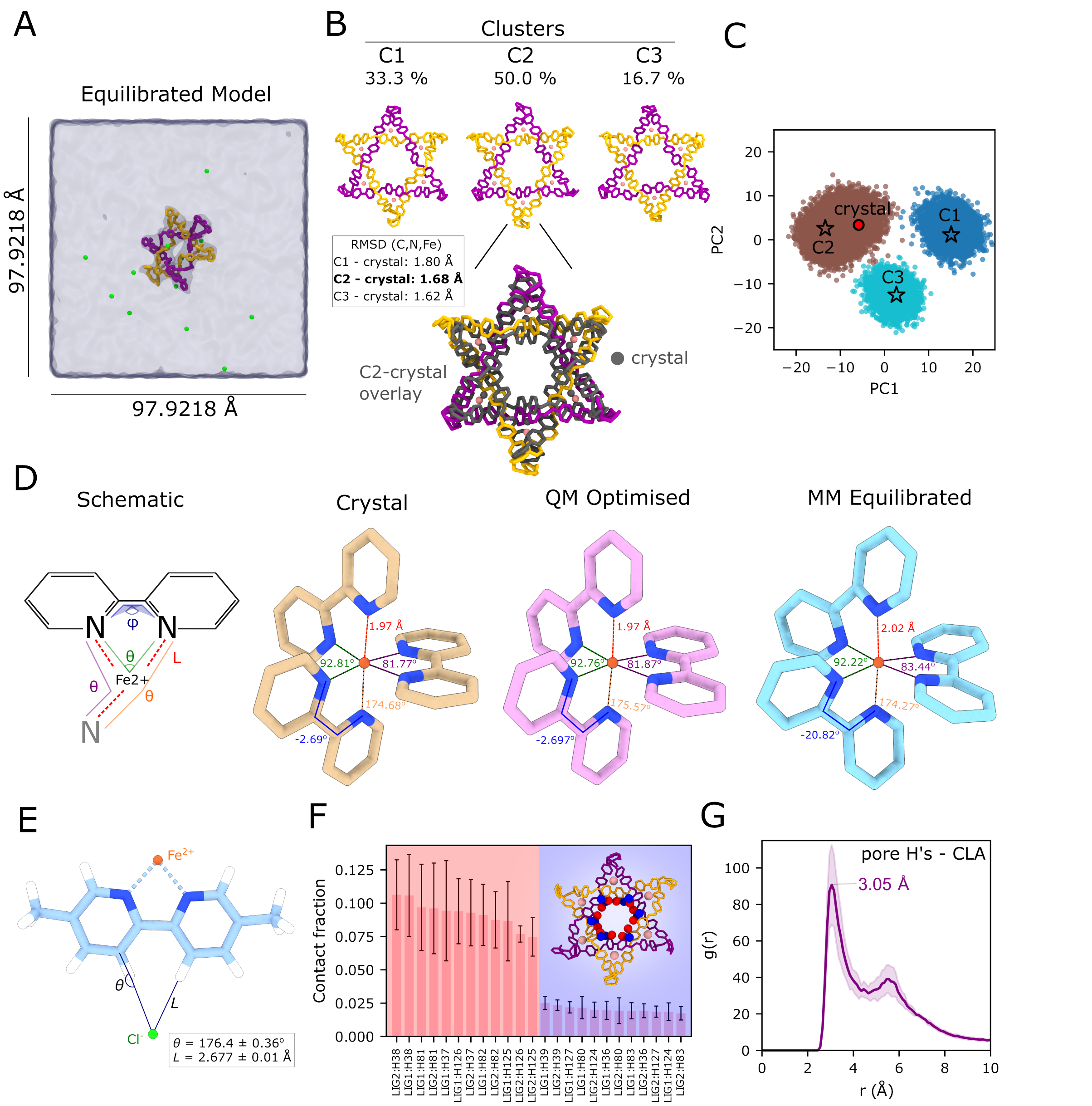

Figure S2. Validation of the SoD force-field parameterisation and Fe-N harmonic bond restraint treatment.

(A) Equilibrated solvated SoD model, neutralised with 12 Cl^- ions. **(B)** Representative structures of the three clusters identified with TTClust, with cluster populations and the RMSD (C, N, Fe) of each to the crystal structure; the C2-crystal overlay is shown below. **(C)** Principal component analysis of the pooled 6 × 250 ns simulations, with frames coloured by cluster and the crystal structure projected onto the same components. **(D)** Octahedral coordination geometry of Fe(II)-tris(5,5′-dimethyl-2,2′-bipyridine) in the crystal, the r²SCAN-3c-optimised structure and the MM-equilibrated system, showing the Fe-N distance, N-Fe-N angles and bipyridine dihedral (φ). Hydrogens and 5,5′-methyl groups are omitted for clarity. **(E)** A single bipyridine ligand from the optimised complex with a Cl^- ion added. **(F)** The 24 highest Cl^--SoD contact fractions (≤ 3.5 Å) across the pooled simulations, for central (red) and peripheral (blue)

pore hydrogens (see inset). **(G)** Radial distribution function between Cl^- and the SoD pore hydrogens. Error bars and shading show mean ± SD across replicas.

Table S7. Simulation protocol for solvated SoD simulations.

Stage	Step	Ensemble	Duration	Δt (fs)	Thermostat (τ_T, ps)	Barostat (τ_P, ps)	Restraints (kJ mol^{-1} nm^{-2})	Notes
Minimisation	-	-	≤ 5000 steps	-	-	-	4000	Steepest descent
Equilibration	1	NVT	125 ps	1	Berendsen (1.0)	-	4000	Velocities generated at 293 K
	2	NVT	125 ps	1	Berendsen (1.0)	-	2000	Continuation, velocities retained
	3	NPT	125 ps	1	Berendsen (1.0)	Berendsen (5.0)	1000	
	4	NPT	500 ps	2	Berendsen (1.0)	Berendsen (5.0)	500	
	5	NPT	500 ps	2	Berendsen (1.0)	Berendsen (5.0)	200	
	6	NPT	250 ns	2	v-rescale (1.0)	Parrinello-Rahman (5.0)	0	
Production	-	NPT	250 ns	2	v-rescale (1.0)	Parrinello-Rahman (5.0)	0	

Simulations were pooled and SoD structures clustered into three clusters using TTClust[8] to probe the distribution of conformational states sampled (**Fig. S2B**). Principal component analysis (PCA) in MDAnalysis showed that the three representative frames occupy distinct regions of the conformational space, with the crystal structure projecting onto the most populated cluster (C2; 50.0%) (**Fig. S2C**). Heavy atoms of all clusters were aligned against the CHARMM-GUI energetically minimised crystal structure (mean RMSD = 1.70 ± 0.09 Å), suggesting close retention of the experimental structure. Overlaying the crystal structure with the representative structure of the most populated cluster (C2; RMSD = 1.68 Å; **Fig. S2B**) shows that the equilibrated SoD relaxes into a slightly more open conformation than the crystal.

To obtain energy-minimised Fe(II)-tris(bipyridine) coordination spheres for comparison with the solvated SoD simulations and the crystal structure, geometry optimisations were performed in ORCA v5.0.4[9] using the composite r²SCAN-3c method[10] with implicit water solvation (CPCM) and tight convergence criteria (TightOpt). Because SoD is too large for a full QM treatment, a cluster approach was used: each Fe(II) centre was extracted from the CCDC SoD structure together with its three coordinating bipyridine ligands, and the linkers were truncated at the 5,5'-positions to leave methyl groups. Hydrogens were then added with

ChimeraX[11], giving six separate Fe(II)-tris(5,5′ -dimethyl-2,2′ -bipyridine) complexes. Each complex was optimised in two steps: first with the Fe(II) ion and the bipyridine heavy atoms constrained while hydrogens and methyl groups relaxed, then with all constraints removed. All optimisations used a charge of +2 and a singlet spin state (multiplicity 1), consistent with low-spin Fe(II).

MDAnalysis[6] was then used to measure octahedral Fe(II) coordination parameters across the solvated SoD simulations, QM-geometrically optimised Fe(II)-tris-5,5'dimethylbipyridine, the crystal structure and the CHARMM-GUI-minimised crystal structure and visualised in (**Table S8**; **Fig. S2D)**. Although our MD model represents the complete SoD catenane, rather than the isolated Fe-bipyridine complexes modelled in our QM calculations and used in most of the previously published studies[1, 12, 13], the calculated Fe-N bond lengths and N-Fe-N coordination angles remain in close agreement with experimental measurements for these simpler complexes. This agreement indicates that the local Fe^{2+}-bipyridine octahedral coordination environment is well preserved within the mechanically interlocked SoD framework with our CGenFF + CHARMM36 parameter set with the Fe-N harmonic distance restraints.

Notably, the N1-C2-C2'-N1' dihedral deviates from ~-2.7° in the crystal and QM structures to ~-20.8° in the MD ensemble, respectively (**Table S8**). This dihedral, corresponding to (**NG2R60-CG2R67-CG2R67-NG2R60**) was assigned by CGenFF from the 2,2'-bipyridine (22BPY) parameters (zero penalty).

Table S8. Mean Fe(II) coordination geometric descriptors.

Models		Parameters				
		Bond lengths (Å)	Bond angles (°)			Dihedrals (°)
			N-Fe-N			
		N-Fe	Intra-bipyridine	Inter-bipyridine		N-C-C-N
				cis	trans	
This study	QM	1.97 ± 0.00	81.87 ± 0.01	92.76 ± 0.00	175.57 ± 0.03	-2.70 ± 0.07
	MD	2.02 ± 0.00	83.44 ± 0.02	92.22 ± 0.01	174.27 ± 0.03	-20.82 ± 0.41
Previously published data	SoD crystal[a] (Leigh, et al)[1]	1.97 ± 0.02	81.77 ± 0.65	92.81 ± 2.92	174.68 ± 1.31	-2.69 ± 4.02
	$[Fe(bpy)_3]$ Crystal (Dick, S. 1998)[13]	1.97	81.8	-	-	6.40
	$[Fe(bpy)_3]$ QM (Daku, et al. 2010)[12]	2.00	81.0	-	-	3.00
	$[Fe(bpy)_3]$ AIMD (Daku, et al. 2010)[12]	1.98	81.5	-	-	0.60
CHARMM-GUI optimised crystal model		2.05 ± 0.04	83.4 ± 1.4	92.22 ±4.21	174.96 ± 3.04	-5.22 ± 4.79

[a] Measured using MDAnalysis from deposited SoD crystallographic model.

For free 2,2'-bipyridine (22BPY), QM calculations in vacuum and solution place the global N1-C2-C2'-N1' minimum at 180° (trans), with a twisted-cisoid local minimum at approximately ± 40° and maxima at 0° and ~90° [14, 15]. Metal coordination, however, locks bipyridine into a near-planar cis conformation[14, 16, 17]: reported dihedrals for Fe(II)-tris(bipyridine) range from 0.6° to 6.4° [1, 12, 13], and the SoD crystal structure [1] has a mean value of -2.69 ± 4.02°. Because the CGenFF parameters derive from free 22BPY, this torsional term is intrinsically biased away from planarity. To quantify this bias, we compared QM and CGenFF torsional profiles for free 22BPY (**Fig. S3**). The 22BPY structure (PubChem CID 1474) was optimised in vacuum with r^{2}SCAN-3c (TightOpt), followed by a relaxed scan of the N1-C2-C2'-N1' dihedral from -180° to 180° in 1° steps (r^{2}SCAN-3c, TightSCF, TightOpt), with energies converted to kJ mol^{-1} using conversion factor of 2625.499639 kJ mol^{-1} $Hartree^{-1}$.

MM energies were calculated in GROMACS using the CGenFF parameters for 22BPY. Each QM-optimised rotamer was placed in a 50 x 50 x 50 Å vacuum box, and the N1-C2-C2'-N1' dihedral was restrained to its QM scan angle (harmonic restraint, $\Delta\varphi$=0°, k = 100,000 kJ mol^{-1} rad^{-2}). Structures were minimized by steepest descent (tolerance 0.1 kJ mol-1 nm-1, maximum 50,000 steps) using the Verlet cutoff scheme (nstlist = 10, rlist = 1.2 nm), force-switched van der Waals interactions between 1.0 and 1.2 nm, a 1.2 nm Coulomb cut-off and no bond constraints. The MM energy at each angle was taken as the final potential energy minus the restraint contribution, and the QM and MM profiles were each expressed relative to their own minimum. The CGenFF profile has a global minimum at 180° and local minima at

approximately ±50° (Fig. S3) so the torsional term is intrinsically biased towards a twisted conformation. In SoD this bias is partly opposed by the Fe-N distance restraints. Despite the torsional deviation, the first coordination sphere remains well reproduced: Fe-N distances are 0.05 Å (2.5%) longer than in the crystal structure, and the bite, cis and trans N-Fe-N angles differ from the crystal values by 1.7°, 0.6° and 0.4°, respectively.

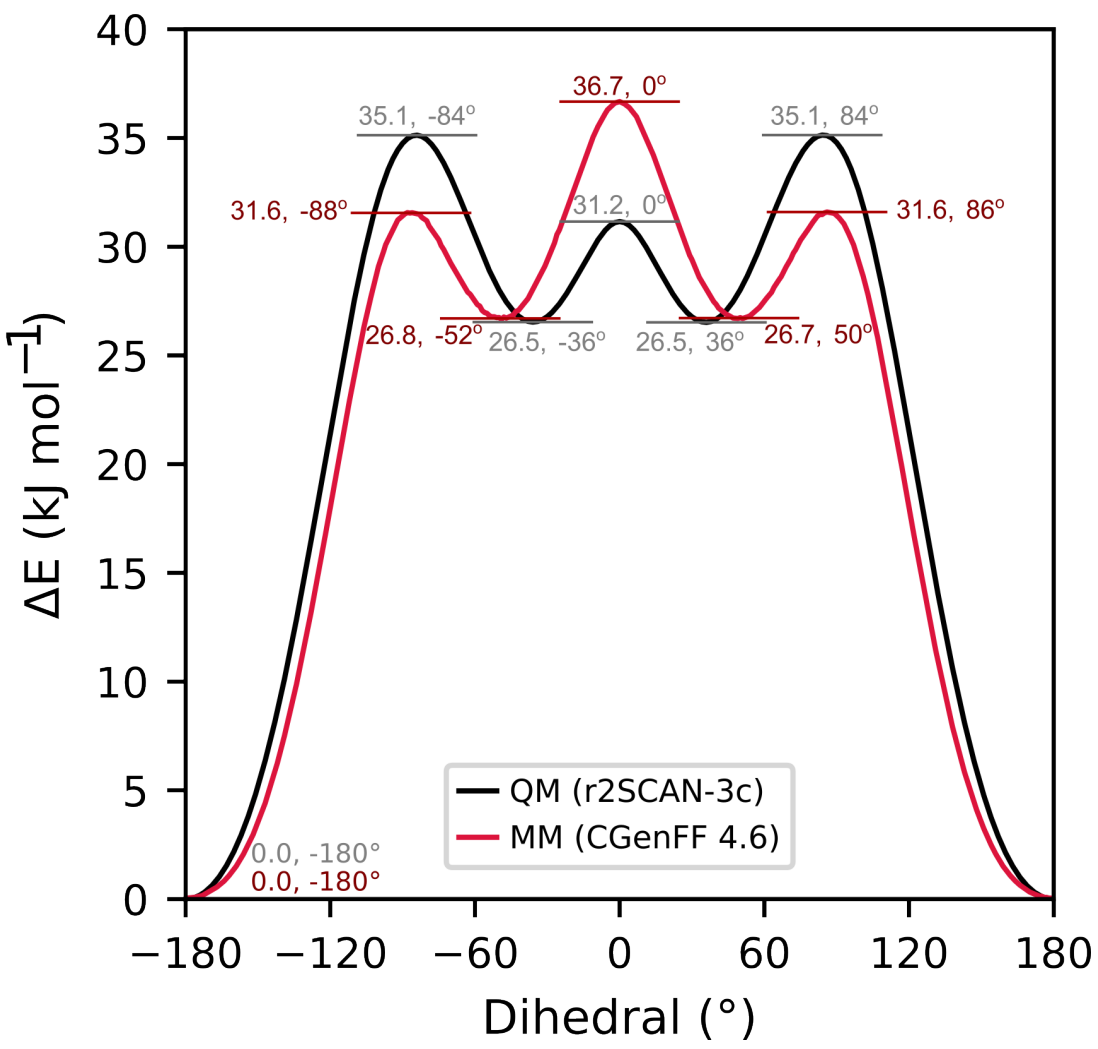

Figure S3. 2,2'-bipyridine N1-C2-C2'-N1' torsional scan profiles for QM (r2-Scan-3c) and MM (CGenFF).

To test whether this torsional deviation affects the structure and dynamics of SoD, the solution simulations were repeated with each N1-C2-C2′-N1′ dihedral restrained to its crystal value ($\Delta\varphi = 0°$, k = 100,000 kJ mol^{-1} rad^{-2}). Representative clustered structures from the restrained and unrestrained simulations deviate similarly from the crystal structure (RMSD = 1.68 ± 0.41 and 1.70 ± 0.09 Å, respectively; **Fig. S4A**). However, the two systems sample conformational space differently: PCA of each system shows that the unrestrained simulations occupy three distinct states across the six replicas, whereas the restrained simulations sample a continuum of asymmetric pore conformations without distinct clusters (**Fig. S4B**). Alignment between representative structures of the restrained and unrestrained systems reveals increased compaction in the restrained system (**Fig. S4C**). Pore radius was analysed across trajectories (**Fig. S4D**), revealing a substantially smaller minimum pore radius for SoD with dihedral restraints compared to the unrestrained system (2.18 ± 0.13 Å and 3.10 ± 0.17 Å). Interactions of Cl^- with pore-lining SoD hydrogens increased substantially in the restrained system (**Fig. S4E**). The distribution of the total number of contacts between SoD and Cl^- per bound chloride (< 3.5 Å) reveal that the restrained system samples an ensemble of states with higher SoD - Cl^- contact numbers (**Fig. S4F**). RDF analysis reveals higher coordination numbers and shorter peak coordination bond distances in the restrained system compared to the unrestrained system (2.95 Å and 3.05 Å, respectively) (**Fig. S4G**), with longer Cl^- binding

residence times in the restrained system (**Fig. S4H**). Together, this data suggests that restraining the N1-C2-C2'-N1' dihedral to the experimental values results in smaller and more asymmetric internal SoD pore structures, allowing for tighter and more numerous interactions between Cl^- ions and the central SoD cavity. To determine whether these differences influence ion transport, electric-field simulations were performed at −100, −50, +50, and +100 mV for the N1-C2-C2′-N1′-restrained SoD model, both with and without SoD centre-of-mass (COM) restraints. The dihedral-restrained system exhibited substantially reduced conductance relative to the unrestrained model (0.68 ± 0.05 nS and 2.05 ± 0.03 nS, respectively for COM-restrained simulations) (**Table S14E**). This reduction coincides with the narrower pore and stronger, longer-lived interactions between chloride ions and the pore observed in the equilibrium simulations.

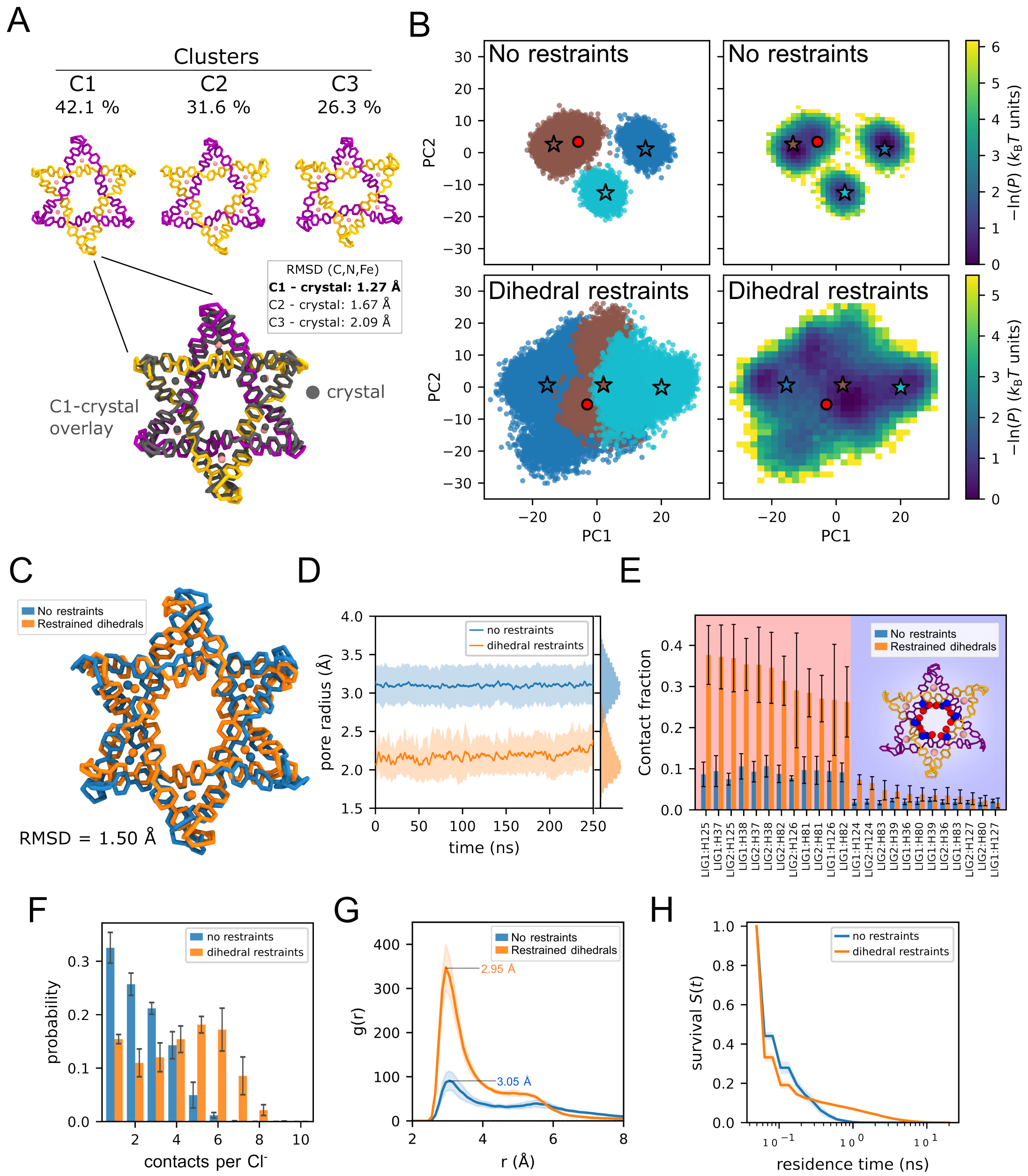

Figure S4. Effect of N1-C2-C2′-N1′ dihedral restraints on SoD structure and Cl⁻ binding.

(A) Representative structures of the three clusters identified with TTClust for the dihedral-restrained system, with cluster populations and the RMSD (C, N, Fe) of each to the crystal structure; the C1-crystal overlay is shown below. **(B)** Principal component analysis of the pooled 6 × 250 ns unrestrained (top) and dihedral-restrained (bottom) simulations, with frames coloured by cluster (left) and shown as free-energy surfaces (right); stars mark cluster representatives and the red circle marks the projected crystal structure. **(C)** Overlay of representative unrestrained (blue) and dihedral-restrained (orange) structures. **(D)** Minimum pore radius over time. **(E)** The highest Cl⁻-SoD contact fractions (≤ 3.5 Å) for the unrestrained (blue) and dihedral-restrained (orange) systems, for central (red background) and peripheral

(blue background) pore hydrogens (see inset). **(F)** Distribution of SoD contacts per bound Cl^- (≤ 3.5 Å). **(G)** Radial distribution function between Cl^- and the SoD pore hydrogens. **(H)** Survival function, S(t), of Cl^- residence times. Error bars and shading show mean ± SD across replicas.

After correcting for the difference in bulk electrolyte conductivity between simulation and experiment, the restrained and unrestrained models give conductances of 0.52 ± 0.04 and 1.54 ± 0.02 nS, respectively (**Table S17. A, D**). Compared with the ~1.5 nS estimated from the slope of the published SoD I-V trace[18], these correspond to 35% and 103% of the experimental value, respectively. Because the dihedral restraints reduce the predicted conductance well below this value, we propose that explicitly restraining the N1-C2-C2′-N1′ torsion gives an overly restrictive representation of the SoD pore. We therefore retain the unrestrained CGenFF/CHARMM36 model with Fe-N distance restraints for all subsequent simulations. This choice does not imply that the unrestrained model fully describes the torsional potential of Fe(II)-coordinated bipyridine; rather, it balances preservation of the experimentally observed first coordination sphere against avoidance of restraints that substantially perturb the pore.

S2. Simulation models and protocols

S2.1 Model compositions

Table S9. Composition and dimensions of each simulation system.

Lipid	Replica	Phospholipid		Cholesterol		SoD rings	Fe(II)	Cl^-	K^+	TIP3P	Total atoms	Dimensions (Å)
		Upper	Lower	Upper	Lower							
**A. Horizontally embedded SoD monomer in POPC:cholesterol (2:1) bilayer, 85 × 85 Å patch**												
POPC	1	82	82	41	41	2	6	182	170	9676	58042	85 × 85 × 85
POPC	2	82	82	41	41	2	6	180	168	9678	58044	85 × 85 × 85
POPC	3	82	82	41	41	2	6	182	170	9681	58057	85 × 85 × 85
POPC	4	82	82	41	41	2	6	182	170	9683	58063	85 × 85 × 85
POPC	5	82	82	41	41	2	6	180	168	9676	58038	85 × 85 × 85
POPC	6	82	82	41	41	2	6	181	169	9686	58070	85 × 85 × 85
**B. Empty POPC:cholesterol (2:1) bilayer (negative control), 85 × 85 Å patch**												
POPC	1	82	82	41	41	-	-	168	168	9704	57492	85 × 85 × 85
POPC	2	82	82	41	41	-	-	170	170	9661	57367	85 × 85 × 85
POPC	3	82	82	41	41	-	-	167	167	9691	57451	85 × 85 × 85
POPC	4	82	82	41	41	-	-	168	168	9676	57408	85 × 85 × 85
POPC	5	82	82	41	41	-	-	169	169	9665	57377	85 × 85 × 85
POPC	6	82	82	41	41	-	-	169	169	9707	57503	85 × 85 × 85
**C. 1 M KCl solvated box (no membrane)**												
-	1	-	-	-	-	-	-	369	369	17481	53181	85 × 85 × 85
-	2	-	-	-	-	-	-	369	369	17484	53190	85 × 85 × 85
-	3	-	-	-	-	-	-	369	369	17472	53154	85 × 85 × 85
-	4	-	-	-	-	-	-	369	369	17456	53106	85 × 85 × 85
-	5	-	-	-	-	-	-	369	369	17504	53250	85 × 85 × 85
-	6	-	-	-	-	-	-	369	369	17444	53070	85 × 85 × 85
**D. Horizontally embedded SoD monomer in DDPC, DCPC, DLPC and DMPC bilayers, 85 × 85 Å patch**												
DDPC	1	113	113	-	-	2	6	226	214	10462	53688	85 × 85 × 85
DDPC	2	113	113	-	-	2	6	226	214	10462	53688	85 × 85 × 85
DDPC	3	113	113	-	-	2	6	227	215	10462	53690	85 × 85 × 85
DDPC	4	113	113	-	-	2	6	227	215	10462	53690	85 × 85 × 85
DDPC	5	113	113	-	-	2	6	227	215	10462	53690	85 × 85 × 85
DDPC	6	113	113	-	-	2	6	227	215	10462	53690	85 × 85 × 85
DCPC	1	113	113	-	-	2	6	218	206	10462	55028	85 × 85 × 85
DCPC	2	113	113	-	-	2	6	218	206	10462	55028	85 × 85 × 85
DCPC	3	113	113	-	-	2	6	217	205	10461	55023	85 × 85 × 85
DCPC	4	113	113	-	-	2	6	218	206	10462	55028	85 × 85 × 85
DCPC	5	113	113	-	-	2	6	218	206	10462	55028	85 × 85 × 85
DCPC	6	113	113	-	-	2	6	218	206	10461	55025	85 × 85 × 85
DLPC	1	113	113	-	-	2	6	208	196	10445	56313	85 × 85 × 85
DLPC	2	113	113	-	-	2	6	209	197	10448	56324	85 × 85 × 85
DLPC	3	113	113	-	-	2	6	209	197	10444	56312	85 × 85 × 85
DLPC	4	113	113	-	-	2	6	208	196	10439	56295	85 × 85 × 85
DLPC	5	113	113	-	-	2	6	209	197	10450	56330	85 × 85 × 85
DLPC	6	113	113	-	-	2	6	209	197	10441	56303	85 × 85 × 85
DMPC	1	113	113	-	-	2	6	191	179	10165	58151	85 × 85 × 85
DMPC	2	113	113	-	-	2	6	189	177	10139	58069	85 × 85 × 85
DMPC	3	113	113	-	-	2	6	190	178	10185	58209	85 × 85 × 85
DMPC	4	113	113	-	-	2	6	191	179	10176	58184	85 × 85 × 85
DMPC	5	113	113	-	-	2	6	191	179	10156	58124	85 × 85 × 85
DMPC	6	113	113	-	-	2	6	189	177	10161	58135	85 × 85 × 85
**E. Empty DDPC, DCPC, DLPC and DMPC bilayers, 85 × 85 Å patch**												

Lipid	Replica	Phospholipid		Cholesterol		SoD	Fe(II)	Cl^-	K^+	TIP3P	Total	Dimensions (Å)
		Upper	Lower	Upper	Lower	rings					atoms	
DDPC	1	113	113	-	-	-	-	214	214	10462	43058	85 × 85 × 85
DDPC	2	113	113	-	-	-	-	215	215	10462	53060	85 × 85 × 85
DDPC	3	113	113	-	-	-	-	214	214	10462	53058	85 × 85 × 85
DDPC	4	113	113	-	-	-	-	214	214	10462	53058	85 × 85 × 85
DDPC	5	113	113	-	-	-	-	214	214	10462	53058	85 × 85 × 85
DDPC	6	113	113	-	-	-	-	215	215	10462	53060	85 × 85 × 85
DCPC	1	113	113	-	-	-	-	206	206	10461	54395	85 × 85 × 85
DCPC	2	113	113	-	-	-	-	207	207	10461	54397	85 × 85 × 85
DCPC	3	113	113	-	-	-	-	206	206	10461	54395	85 × 85 × 85
DCPC	4	113	113	-	-	-	-	206	206	10461	54395	85 × 85 × 85
DCPC	5	113	113	-	-	-	-	204	204	10462	54394	85 × 85 × 85
DCPC	6	113	113	-	-	-	-	205	205	10462	54396	85 × 85 × 85
DLPC	1	113	113	-	-	-	-	196	196	10448	55692	85 × 85 × 85
DLPC	2	113	113	-	-	-	-	197	197	10445	55685	85 × 85 × 85
DLPC	3	113	113	-	-	-	-	196	196	10455	55713	85 × 85 × 85
DLPC	4	113	113	-	-	-	-	196	196	10451	55701	85 × 85 × 85
DLPC	5	113	113	-	-	-	-	197	197	10446	55688	85 × 85 × 85
DLPC	6	113	113	-	-	-	-	197	197	10448	55694	85 × 85 × 85
DMPC	1	113	113	-	-	-	-	178	178	10169	57531	85 × 85 × 85
DMPC	2	113	113	-	-	-	-	176	176	10176	57548	85 × 85 × 85
DMPC	3	113	113	-	-	-	-	178	178	10179	57561	85 × 85 × 85
DMPC	4	113	113	-	-	-	-	177	177	10158	57496	85 × 85 × 85
DMPC	5	113	113	-	-	-	-	178	178	10161	57507	85 × 85 × 85
DMPC	6	113	113	-	-	-	-	178	178	10146	57462	85 × 85 × 85
**F. Embedded SoD dimer in POPC:cholesterol (2:1) bilayer, 85 × 85 Å patch**												
POPC	1	74	74	37	37	4	6	197	173	9816	56362	85 × 85 × 85
POPC	2	74	74	37	37	4	6	199	175	9838	56432	85 × 85 × 85
POPC	3	74	74	37	37	4	6	199	175	9874	56540	85 × 85 × 85
POPC	4	74	74	37	37	4	6	199	175	9847	56459	85 × 85 × 85
POPC	5	74	74	37	37	4	6	198	174	9845	56451	85 × 85 × 85
POPC	6	74	74	37	37	4	6	202	178	9861	56507	85 × 85 × 85
**G. Vertically embedded SoD monomer in POPC:cholesterol (2:1) bilayer**												
POPC	1-6	80	80	40	40	2	6	185	173	9931	58129	86 × 86 × 85
**H. Horizontally embedded SoD monomer in POPC:cholesterol (2:1) bilayer, 150 × 150 Å patch**												
POPC	1	256	256	128	128	2	6	1306	1294	69706	299888	150 × 150 × 140
POPC	2	256	256	128	128	2	6	1306	1294	69727	299951	150 × 150 × 140
POPC	3	256	256	128	128	2	6	1304	1292	69699	299863	150 × 150 × 140
POPC	4	256	256	128	128	2	6	1304	1292	69686	299824	150 × 150 × 140
POPC	5	256	256	128	128	2	6	1303	1291	69706	299882	150 × 150 × 140
POPC	6	256	256	128	128	2	6	1303	1291	69689	299831	150 × 150 × 140
**I. Empty POPC:cholesterol (2:1) bilayer, 150 × 150 Å patch**												
POPC	1	256	256	128	128	-	-	1290	1290	69679	299169	150 × 150 × 140
POPC	2	256	256	128	128	-	-	1291	1291	69709	299261	150 × 150 × 140
POPC	3	256	256	128	128	-	-	1291	1291	69730	299324	150 × 150 × 140
POPC	4	256	256	128	128	-	-	1297	1297	69667	299147	150 × 150 × 140
POPC	5	256	256	128	128	-	-	1292	1292	69664	199128	150 × 150 × 140
POPC	6	256	256	128	128	-	-	1293	1293	69679	299175	150 × 150 × 140
**J. Solvated SoD situated below an electropore**												
DPhPC	1	182 (total)		-	-	2	6	489	477	25545	106247	98 × 98 × 119

Table S10. Initial and final simulation box dimensions.

System COM-Restraint	Replica	NVT/NPT	Initial dimensions (nm)	Production dimensions (nm)
Monomeric, Horizontal, POPC:cholesterol (2:1), NBFIX, 85 x 85 Å, 293 K				
Restrained	1	NVT	8.509 x 8.509 x 8.500	7.964 x 7.964 x 8.652
	2	NVT	8.509 x 8.509 x 8.500	7.883 x 7.883 x 8.877
	3	NVT	8.509 x 8.509 x 8.500	7.947 x 7.947 x 8.691
	4	NVT	8.509 x 8.509 x 8.500	7.949 x 7.949 x 8.681
	5	NVT	8.509 x 8.509 x 8.500	7.955 x 7.955 x 8.666
	6	NVT	8.509 x 8.509 x 8.500	7.853 x 7.853 x 8.886
Unrestrained	1	NVT	8.509 x 8.509 x 8.500	7.994 x 7.994 x 8.612
	2	NVT	8.509 x 8.509 x 8.500	7.969 x 7.969 x 8.623
	3	NVT	8.509 x 8.509 x 8.500	7.899 x 7.899 x 8.773
	4	NVT	8.509 x 8.509 x 8.500	7.765 x 7.765 x 9.118
	5	NVT	8.509 x 8.509 x 8.500	7.848 x 7.848 x 8.926
	6	NVT	8.509 x 8.509 x 8.500	7.850 x 7.850 x 8.895
Empty POPC:cholesterol (2:1) membrane patch, NBFIX, 85 x 85 Å, 293 K				
-	1	NVT	8.509 x 8.509 x 8.500	7.403 x 7.403 x 9.931
	2	NVT	8.509 x 8.509 x 8.500	7.294 x 7.294 x 10.145
	3	NVT	8.509 x 8.509 x 8.500	7.348 x 7.348 x 10.087
	4	NVT	8.509 x 8.509 x 8.500	7.364 x 7.364 x 9.998
	5	NVT	8.509 x 8.509 x 8.500	7.428 x 7.428 x 9.844
	6	NVT	8.509 x 8.509 x 8.500	7.363 x 7.363 x 10.012
Salt solution box, NBFIX, 85 x 85 Å, 293 K				
-	1	NVT	8.500 x 8.500 x 8.500	8.092 x 8.092 x 8.092
	2	NVT	8.500 x 8.500 x 8.500	8.091 x 8.091 x 8.091
	3	NVT	8.500 x 8.500 x 8.500	8.095 x 8.095 x 8.095
	4	NVT	8.500 x 8.500 x 8.500	8.080 x 8.080 x 8.080
	5	NVT	8.500 x 8.500 x 8.500	8.084 x 8.084 x 8.084
	6	NVT	8.500 x 8.500 x 8.500	8.092 x 8.092 x 8.092
Monomeric, Horizontal, POPC:cholesterol (2:1), prosECCo75 (all charges scaled), 85 x 85 Å, 293 K				
Restrained	1	NVT	8.509 x 8.509 x 8.500	7.744 x 7.744 x 9.297
	2	NVT	8.509 x 8.509 x 8.500	7.898 x 7.898 x 8.989
	3	NVT	8.509 x 8.509 x 8.500	7.762 x 7.762 x 9.255
	4	NVT	8.509 x 8.509 x 8.500	7.812 x 7.812 x 9.160
	5	NVT	8.509 x 8.509 x 8.500	7.819 x 7.819 x 9.133
	6	NVT	8.509 x 8.509 x 8.500	7.781 x 7.781 x 9.236
Unrestrained	1	NVT	8.509 x 8.509 x 8.500	7.765 x 7.765 x 9.255

System COM-Restraint	Replica	NVT/NPT	Initial dimensions (nm)	Production dimensions (nm)
	2	NVT	8.509 x 8.509 x 8.500	7.805 x 7.805 x 9.187
	3	NVT	8.509 x 8.509 x 8.500	7.808 x 7.808 x 9.112
	4	NVT	8.509 x 8.509 x 8.500	7.704 x 7.704 x 9.423
	5	NVT	8.509 x 8.509 x 8.500	7.604 x 7.604 x 9.644
	6	NVT	8.509 x 8.509 x 8.500	7.683 x 7.683 x 9.479
Empty POPC:cholesterol (2:1) membrane patch, prosECCo75 (all charges scaled), 85 x 85 Å, 293 K				
-	1	NVT	8.509 x 8.509 x 8.500	7.255 x 7.255 x 10.483
	2	NVT	8.509 x 8.509 x 8.500	7.347 x 7.347 x 10.218
	3	NVT	8.509 x 8.509 x 8.500	7.302 x 7.302 x 10.319
	4	NVT	8.509 x 8.509 x 8.500	7.309 x 7.309 x 10.290
	5	NVT	8.509 x 8.509 x 8.500	7.286 x 7.286 x 10.329
	6	NVT	8.509 x 8.509 x 8.500	7.281 x 7.281 x 10.379
Salt solution box, prosECCo75 (all charges scaled), 85 x 85 Å, 293 K				
-	1	NVT	8.500 x 8.500 x 8.500	8.158 x 8.158 x 8.158
	2	NVT	8.500 x 8.500 x 8.500	8.164 x 8.164 x 8.164
	3	NVT	8.500 x 8.500 x 8.500	8.149 x 8.149 x 8.149
	4	NVT	8.500 x 8.500 x 8.500	8.158 x 8.158 x 8.158
	5	NVT	8.500 x 8.500 x 8.500	8.158 x 8.158 x 8.158
	6	NVT	8.500 x 8.500 x 8.500	8.150 x 8.150 x 8.150
Monomeric, Horizontal, POPC:cholesterol (2:1), prosECCo75 (only Fe(II) scaled), 85 x 85 Å, 293 K				
Restrained	3	NVT	8.509 x 8.509 x 8.500	7.808 x 7.808 x 9.112
	4	NVT	8.509 x 8.509 x 8.500	7.704 x 7.704 x 9.423
	5	NVT	8.509 x 8.509 x 8.500	7.604 x 7.604 x 9.644
	6	NVT	8.509 x 8.509 x 8.500	7.683 x 7.683 x 9.479
	5	NVT	8.509 x 8.509 x 8.500	7.847 x 7.847 x 8.916
	6	NVT	8.509 x 8.509 x 8.500	7.845 x 7.845 x 8.947
Unrestrained	1	NVT	8.509 x 8.509 x 8.500	7.673 x 7.673 x 9.317
	2	NVT	8.509 x 8.509 x 8.500	7.839 x 7.839 x 8.922
	3	NVT	8.509 x 8.509 x 8.500	7.759 x 7.759 x 9.134
	4	NVT	8.509 x 8.509 x 8.500	7.811 x 7.811 x 8.987
	5	NVT	8.509 x 8.509 x 8.500	7.818 x 7.818 x 8.967
	6	NVT	8.509 x 8.509 x 8.500	7.727 x 7.727 x 9.195
Monomeric, Horizontal, DDPC, NBFIX, 85 x 85 Å, 293 K				
Restrained	1	NVT	8.511 x 8.511 x 8.500	8.710 x 8.710 x 6.890
	2	NVT	8.511 x 8.511 x 8.500	8.627 x 8.627 x 7.020
	3	NVT	8.511 x 8.511 x 8.500	8.672 x 8.672 x 6.920

System COM-Restraint	Replica	NVT/NPT	Initial dimensions (nm)	Production dimensions (nm)
	4	NVT	8.511 x 8.511 x 8.500	8.632 x 8.632 x 7.028
	5	NVT	8.511 x 8.511 x 8.500	8.756 x 8.756 x 6.833
	6	NVT	8.511 x 8.511 x 8.500	8.694 x 8.694 x 6.924
Unrestrained	1	NVT	8.511 x 8.511 x 8.500	8.718 x 8.718 x 6.888
	2	NVT	8.511 x 8.511 x 8.500	8.603 x 8.603 x 7.047
	3	NVT	8.511 x 8.511 x 8.500	8.660 x 8.660 x 6.956
	4	NVT	8.511 x 8.511 x 8.500	8.793 x 8.793 x 6.792
	5	NVT	8.511 x 8.511 x 8.500	8.786 x 8.786 x 6,755
	6	NVT	8.511 x 8.511 x 8.500	8.728 x 8.728 x 6.859
Empty DDPC membrane patch, NBFIX, 85 x 85 Å, 293 K				
-	1	NVT	8.511 x 8.511 x 8.500	8.407 x 8.407 x 7.281
	2	NVT	8.511 x 8.511 x 8.500	8.425 x 8.425 x 7.264
	3	NVT	8.511 x 8.511 x 8.500	8.314 x 8.314 x 7.462
	4	NVT	8.511 x 8.511 x 8.500	8.367 x 8.367 x 7.365
	5	NVT	8.511 x 8.511 x 8.500	8.526 x 8.526 x 7.096
	6	NVT	8.511 x 8.511 x 8.500	8.470 x 8.470 x 7.220
Monomeric, Horizontal, DCPC, NBFIX, 85 x 85 Å, 293 K				
Restrained	1	NVT	8.511 x 8.511 x 8.500	8.631 x 8.631 x 7.172
	2	NVT	8.511 x 8.511 x 8.500	8.620 x 8.620 x 7.221
	3	NVT	8.511 x 8.511 x 8.500	8.653 x 8.653 x 7.130
	4	NVT	8.511 x 8.511 x 8.500	8.516 x 8.516 x 7.367
	5	NVT	8.511 x 8.511 x 8.500	8.735 x 8.735 x 6.994
	6	NVT	8.511 x 8.511 x 8.500	8.587 x 8.587 x 7.244
Unrestrained	1	NVT	8.511 x 8.511 x 8.500	8.648 x 8.648 x 7.190
	2	NVT	8.511 x 8.511 x 8.500	8.671 x 8.671 x 7.123
	3	NVT	8.511 x 8.511 x 8.500	8.525 x 8.525 x 7.355
	4	NVT	8.511 x 8.511 x 8.500	8.768 x 8.768 x 6.958
	5	NVT	8.511 x 8.511 x 8.500	8.677 x 8.677 x 7.102
	6	NVT	8.511 x 8.511 x 8.500	8.593 x 8.593 x 7.234
Empty DCPC membrane patch, NBFIX, 85 x 85 Å, 293 K				
-	1	NVT	8.511 x 8.511 x 8.500	8.383 x 8.383 x 7.517
	2	NVT	8.511 x 8.511 x 8.500	8.239 x 8.239 x 7.788
	3	NVT	8.511 x 8.511 x 8.500	8.451 x 8.451 x 7.388
	4	NVT	8.511 x 8.511 x 8.500	8.381 x 8.381 x 7.498
	5	NVT	8.511 x 8.511 x 8.500	8.377 x 8.377 x 7.544
	6	NVT	8.511 x 8.511 x 8.500	8.426 x 8.426 x 7.458

System COM-Restraint	Replica	NVT/NPT	Initial dimensions (nm)	Production dimensions (nm)
Monomeric, Horizontal, DLPC, NBFIX, 85 x 85 Å, 293 K				
Restrained	1	NVT	8.511 x 8.511 x 8.500	8.445 x 8.445 x 7.675
	2	NVT	8.511 x 8.511 x 8.500	8.617 x 8.617 x 7.353
	3	NVT	8.511 x 8.511 x 8.500	8.493 x 8.493 x 7.558
	4	NVT	8.511 x 8.511 x 8.500	8.681 x 8.681 x 7.274
	5	NVT	8.511 x 8.511 x 8.500	8.503 x 8.503 x 7.564
	6	NVT	8.511 x 8.511 x 8.500	8.591x 8.591 x 7.388
Unrestrained	1	NVT	8.511 x 8.511 x 8.500	8.522 x 8.522 x 7.509
	2	NVT	8.511 x 8.511 x 8.500	8.471 x 8.471 x 7.615
	3	NVT	8.511 x 8.511 x 8.500	8.575 x 8.575 x 7.441
	4	NVT	8.511 x 8.511 x 8.500	8.511 x 8.511 x 7.545
	5	NVT	8.511 x 8.511 x 8.500	8.550 x 8.550 x 7.466
	6	NVT	8.511 x 8.511 x 8.500	8.579 x 8.579 x 7.434
Empty DLPC membrane patch, NBFIX, 85 x 85 Å, 293 K				
-	1	NVT	8.511 x 8.511 x 8.500	8.288 x 8.288 x 7.848
	2	NVT	8.511 x 8.511 x 8.500	8.281 x 8.281 x 7.878
	3	NVT	8.511 x 8.511 x 8.500	8.341 x 8.341 x 7.754
	4	NVT	8.511 x 8.511 x 8.500	8.337 x 8.377 x 7.774
	5	NVT	8.511 x 8.511 x 8.500	8.291 x 8.291 x 7.866
	6	NVT	8.511 x 8.511 x 8.500	8.302 x 8.302 x 7.866
Monomeric, Horizontal, DMPC, NBFIX, 85 x 85 Å, 293 K				
Restrained	1	NVT	8.511 x 8.511 x 8.500	8.326 x 8.326 x 8.101
	2	NVT	8.511 x 8.511 x 8.500	8.322 x 8.322 x 8.080
	3	NVT	8.511 x 8.511 x 8.500	8.424 x 8.424 x 7.932
	4	NVT	8.511 x 8.511 x 8.500	8.353 x 8.353 x 8.066
	5	NVT	8.511 x 8.511 x 8.500	8.342 x 8.342 x 8.036
	6	NVT	8.511 x 8.511 x 8.500	8.352 x 8.352 x 8.011
Unrestrained	1	NVT	8.511 x 8.511 x 8.500	8.479 x 8.479 x 7.805
	2	NVT	8.511 x 8.511 x 8.500	8.352 x 8.352 x 8.011
	3	NVT	8.511 x 8.511 x 8.500	8.464 x 8.464 x 7.873
	4	NVT	8.511 x 8.511 x 8.500	8.155 x 8.155 x 8.399
	5	NVT	8.511 x 8.511 x 8.500	8.338 x 8.338 x 8.070
	6	NVT	8.511 x 8.511 x 8.500	8.413 x 8.413 x 7.926
Empty DMPC membrane patch, NBFIX, 85 x 85 Å, 293 K				
-	1	NVT	8.511 x 8.511 x 8.500	8.217 x 8.217 x 8.237
	2	NVT	8.511 x 8.511 x 8.500	8.150 x 8.150 x 8.375

System COM-Restraint	Replica	NVT/NPT	Initial dimensions (nm)	Production dimensions (nm)
	3	NVT	8.511 x 8.511 x 8.500	8.268 x 8.268 x 8.111
	4	NVT	8.511 x 8.511 x 8.500	8.101 x 8.101 x 8.454
	5	NVT	8.511 x 8.511 x 8.500	7.936 x 7.936 x 8.771
	6	NVT	8.511 x 8.511 x 8.500	8.213 x 8.213 x 8.202
Salt solution box, NBFIX, 85 x 85 Å, 310 K				
-	1	NVT	8.500 x 8.500 x 8.500	8.136 x 8.136 x 8.136
	2	NVT	8.500 x 8.500 x 8.500	8.130 x 8.130 x 8.130
	3	NVT	8.500 x 8.500 x 8.500	8.130 x 8.130 x 8.130
	4	NVT	8.500 x 8.500 x 8.500	8.128 x 8.128 x 8.128
	5	NVT	8.500 x 8.500 x 8.500	8.126 x 8.126 x 8.126
	6	NVT	8.500 x 8.500 x 8.500	8.125 x 8.125 x 8.125
Dimeric, Horizontal, POPC:cholesterol (2:1), NBFIX, 85 x 85 Å, 293 K				
Restrained	1	NVT	8.533 x 8.533 x 8.500	7.529 x 7.529 x 9.402
	2	NVT	8.533 x 8.533 x 8.500	7.549 x 7.549 x 9.392
	3	NVT	8.533 x 8.533 x 8.500	7.620 x 7.620 x 9.263
	4	NVT	8.533 x 8.533 x 8.500	7.621 x 7.621 x 9.243
	5	NVT	8.533 x 8.533 x 8.500	7.563 x 7.563 x 9.369
	6	NVT	8.533 x 8.533 x 8.500	7.568 x 7.568 x 9.361
Monomeric, Horizontal, POPC:cholesterol (2:1), NBFIX, 150 x 150 Å, 293 K				
Restrained	1	NVT	15.035 x 18.035 x 14.000	13.368 x 13.368 x 16.186
	2	NVT	15.035 x 18.035 x 14.000	13.376 x 13.376 x 16.166
	3	NVT	15.035 x 18.035 x 14.000	13.329 x 13.329 x 16.277
	4	NVT	15.035 x 18.035 x 14.000	13.352 x 13.352 x 16.216
	5	NVT	15.035 x 18.035 x 14.000	13.252 x 13.252 x 16.459
	6	NVT	15.035 x 18.035 x 14.000	13.275 x 13.275 x 16.408
Unrestrained	1	NVT	15.035 x 18.035 x 14.000	13.359 x 13.359 x 16.222
	2	NVT	15.035 x 18.035 x 14.000	13.312 x 13.312 x 16.321
	3	NVT	15.035 x 18.035 x 14.000	13.320 x 13.320 x 16.313
	4	NVT	15.035 x 18.035 x 14.000	13.349 x 13.349 x 16.239
	5	NVT	15.035 x 18.035 x 14.000	13.326 x 13.236 x 16.294
	6	NVT	15.035 x 18.035 x 14.000	13.309 x 13.309 x 16.346
Empty POPC:cholesterol (2:1) membrane patch, NBFIX, 150 x 150 Å, 293 K				
-	1	NVT	15.035 x 15.035 x 14.000	13.022 x 13.022 x 17.008
	2	NVT	15.035 x 15.035 x 14.000	13.070 x 13.070 x 16.911
	3	NVT	15.035 x 15.035 x 14.000	12.958 x 12.958 x 17.204
	4	NVT	15.035 x 15.035 x 14.000	12.940 x 12.940 x 17.243

System<br>COM-<br>Restraint	Replica	NVT/NPT	Initial dimensions (nm)	Production dimensions (nm)
	5	NVT	15.035 x 15.035 x 14.000	12.971 x 12.971 x 17.116
	6	NVT	15.035 x 15.035 x 14.000	12.968 x 12.968 x 17.155

S2.2 Simulation protocols

Equilibration followed a six-step protocol, with restraints progressively reduced to zero before production (**Table S11**). Lipid position restraints were applied to the z-coordinate of phospholipid phosphorus (P) and cholesterol hydroxyl oxygen (O3) atoms. Lipid dihedral restraints maintained glycerol C2 stereochemistry (C1-C3-C2-O21; $\varphi_0 = -120°$, $\Delta\varphi = 2.5°$) and the *cis* configuration of the POPC oleoyl double bond (C28-C29-C210-C211; $\varphi_0 = 0°$, $\Delta\varphi = 0°$), where $\Delta\varphi$ is the flat-bottom tolerance. Cholesterol was not dihedral-restrained. Fe-N distance restraints (Table S5) were applied in all steps, and SoD COM restraints, where used, are described in Methods. The first 50 ns of step 6 were discarded, and the remaining 200 ns were used for equilibrium analyses. Production simulations were performed under an applied electric field (see Methods). Systems without SoD followed the same protocol without SoD position restraints, and electropore-insertion simulations followed a separate protocol (see Methods). τT and τP are the thermostat and barostat coupling time constants.

Table S11. Minimisation, equilibration and production protocols.

Stage		Ensemble	Duration	Δ*t* (fs)	Thermostat (τT , ps)	Barostat (τP , ps)	Restraints: Position restraints (kJ mol⁻¹ nm⁻²)		Restraints: Dihedral restraints (kJ mol⁻¹ rad⁻²)
							**SoD**	**Lipid**	**Lipid**
**Minimisation (steepest descent)**		-	≤ 5000 steps	-	-	-	4000	1000	1000
**Equilibration**	**1**	NVT	125 ps	1	Berendsen (1.0)	-	4000	1000	1000
	**2**	NVT	125 ps	1	Berendsen (1.0)	-	2000	400	400
	**3**	NPT	125 ps	1	Berendsen (1.0)	Berendsen (5.0)	1000	400	200
	**4**	NPT	500 ps	2	Berendsen (1.0)	Berendsen (5.0)	500	200	200
	**5**	NPT	500 ps	2	Berendsen (1.0)	Parrinello-Rahman (5.0)	200	40	100
	**6**	NPT	250 ns	2	v-rescale (1.0)	Parrinello-Rahman (5.0)	0	0	0
**Production**		NVT	250 ns	2	v-rescale (1.0)	-	0	0	0

Table S12. Applied electric field strengths for each system.

System: COM-Restraint	System: Replica	L_z (nm)	E, ± 50 mV (V nm^{-1})	E, ± 100 mV (V nm^{-1})
Monomeric, Horizontal, POPC:cholesterol (2:1), NBFIX, 85 x 85 Å, 293 K				
Restrained	1	8.652	5.778×10^{-3}	1.156×10^{-2}
	2	8.877	5.633×10^{-3}	1.127×10^{-2}
	3	8.691	5.753×10^{-3}	1.151×10^{-2}
	4	8.681	5.760×10^{-3}	1.152×10^{-2}
	5	8.666	5.770×10^{-3}	1.154×10^{-2}
	6	8.886	5.627×10^{-3}	1.125×10^{-2}
Unrestrained	1	8.778	5.696×10^{-3}	1.139×10^{-2}
	2	8.707	5.743×10^{-3}	1.149×10^{-2}
	3	8.681	5.760×10^{-3}	1.152×10^{-2}
	4	9.118	5.484×10^{-3}	1.097×10^{-2}
	5	8.926	5.602×10^{-3}	1.120×10^{-2}
	6	8.895	5.621×10^{-3}	1.124×10^{-2}
Empty POPC:cholesterol (2:1) membrane patch, NBFIX, 85 x 85 Å, 293 K				
-	1	9.931	5.035×10^{-3}	1.007×10^{-2}
	2	10.145	4.929×10^{-3}	9.857×10^{-3}
	3	10.087	4.957×10^{-3}	9.914×10^{-3}

System: COM-Restraint	System: Replica	L_z (nm)	E, ± 50 mV ($V\ nm^{-1}$)	E, ± 100 mV ($V\ nm^{-1}$)
	4	9.998	5.001×10^{-3}	1.000×10^{-2}
	5	9.844	5.079×10^{-3}	1.016×10^{-2}
	6	10.012	4.994×10^{-3}	9.988×10^{-3}
Salt solution box, NBFIX, 85 x 85 Å, 293 K				
-	1	8.092	6.179×10^{-3}	1.236×10^{-2}
	2	8.091	6.180×10^{-3}	1.236×10^{-2}
	3	8.095	6.177×10^{-3}	1.235×10^{-2}
	4	8.080	6.188×10^{-3}	1.238×10^{-2}
	5	8.084	6.185×10^{-3}	1.237×10^{-2}
	6	8.092	6.179×10^{-3}	1.236×10^{-2}
Monomeric, Horizontal, POPC:cholesterol (2:1), prosECCo75 (all charges scaled), 85 x 85 Å, 293 K				
Restrained	1	9.297		1.076×10^{-2}
	2	8.989		1.113×10^{-2}
	3	9.255		1.080×10^{-2}
	4	9.160		1.092×10^{-2}
	5	9.133		1.095×10^{-2}
	6	9.236		1.083×10^{-2}
Unrestrained	1	9.255		1.080×10^{-2}
	2	9.187		1.088×10^{-2}
	3	9.112		1.097×10^{-2}
	4	9.423		1.061×10^{-2}
	5	9.644		1.037×10^{-2}
	6	9.479		1.055×10^{-2}
Salt solution box, prosECCo75 (all charges scaled), 85 x 85 Å, 293 K				
	1	8.159	6.129×10^{-3}	1.226×10^{-2}

System COM-Restraint	Replica	L_z (nm)	E, ± 50 mV (V nm^{-1})	E, ± 100 mV (V nm^{-1})
	2	8.164	6.125 x 10^{-3}	1.225 x 10^{-2}
	3	8.149	6.135 x 10^{-3}	1.227 x 10^{-2}
	4	8.158	6.129 x 10^{-3}	1.226 x 10^{-2}
	5	8.158	6.129 x 10^{-3}	1.226 x 10^{-2}
	6	8.150	6.135 x 10^{-3}	1.227 x 10^{-2}
Monomeric, Horizontal, POPC:cholesterol (2:1), prosECCo75 (only Fe(II) scaled), 85 x 85 Å, 293 K				
Restrained	1	8.818		1.134 x 10^{-2}
	2	9.073		1.102 x 10^{-2}
	3	8.897		1.124 x 10^{-2}
	4	8.894		1.124 x 10^{-2}
	5	8.916		1.122 x 10^{-2}
	6	8.947		1.118 x 10^{-2}
Unrestrained	1	9.317		1.073 x 10^{-2}
	2	8.922		1.121 x 10^{-2}
	3	9.134		1.095 x 10^{-2}
	4	8.987		1.113 x 10^{-2}
	5	8.967		1.115 x 10^{-2}
	6	9.195		1.088 x 10^{-2}
Monomeric, Horizontal, DDPC, NBFIX, 85 x 85 Å, 310 K				
Restrained	1	6.890	7.257 x 10^{-3}	1.451 x 10^{-2}
	2	7.020	7.122 x 10^{-3}	1.424 x 10^{-2}
	3	6.920	7.226 x 10^{-3}	1.445 x 10^{-2}
	4	7.028	7.115 x 10^{-3}	1.423 x 10^{-2}
	5	6.833	7.318 x 10^{-3}	1.464 x 10^{-2}
	6	6.924	7.222 x 10^{-3}	1.444 x 10^{-2}

System		L_z (nm)	E, ± 50 mV (V nm^{-1})	E, ± 100 mV (V nm^{-1})
COM-Restraint	Replica			
Unrestrained	1	6.888	7.259 x 10^{-3}	1.452 x 10^{-2}
	2	7.047	7.095 x 10^{-3}	1.419 x 10^{-2}
	3	6.956	7.188 x 10^{-3}	1.438 x 10^{-2}
	4	6.792	7.361 x 10^{-3}	1.472 x 10^{-2}
	5	6.755	7.402 x 10^{-3}	1.480 x 10^{-2}
	6	6.859	7.289 x 10^{-3}	1.458 x 10^{-2}
Empty DDPC membrane patch, NBFIX, 85 x 85 Å, 310 K				
-	1	7.281	6.867 x 10^{-3}	1.373 x 10^{-2}
	2	7.264	6.883 x 10^{-3}	1.377 x 10^{-2}
	3	7.462	6.700 x 10^{-3}	1.340 x 10^{-2}
	4	7.365	6.789 x 10^{-3}	1.358 x 10^{-2}
	5	7.096	7.047 x 10^{-3}	1.409 x 10^{-2}
	6	7.220	6.925 x 10^{-3}	1.385 x 10^{-2}
Monomeric, Horizontal, DCPC, NBFIX, 85 x 85 Å, 310 K				
Restrained	1	7.171	6.972 x 10^{-3}	1.394 x 10^{-2}
	2	7.221	6.924 x 10^{-3}	1.385 x 10^{-2}
	3	7.130	7.013 x 10^{-3}	1.403 x 10^{-2}
	4	7.367	6.787 x 10^{-3}	1.357 x 10^{-2}
	5	6.994	7.149 x 10^{-3}	1.430 x 10^{-2}

System COM-Restraint	Replica	L_z (nm)	E, ± 50 mV (V nm^{-1})	E, ± 100 mV (V nm^{-1})
	6	7.244	6.903×10^{-3}	1.381×10^{-2}
Unrestrained	1	7.190	6.954×10^{-3}	1.391×10^{-2}
	2	7.126	7.017×10^{-3}	1.403×10^{-2}
	3	7.355	6.798×10^{-3}	1.360×10^{-2}
	4	6.958	7.185×10^{-3}	1.437×10^{-2}
	5	7.102	7.040×10^{-3}	1.408×10^{-2}
	6	7.234	6.911×10^{-3}	1.382×10^{-2}
Empty DCPC membrane patch, NBFIX, 85 x 85 Å, 310 K				
-	1	7.517	6.652×10^{-3}	1.330×10^{-2}
	2	7.788	6.420×10^{-3}	1.284×10^{-2}
	3	7.388	6.768×10^{-3}	1.354×10^{-2}
	4	7.498	6.668×10^{-3}	1.334×10^{-2}
	5	7.544	6.627×10^{-3}	1.325×10^{-2}
	6	7.458	6.704×10^{-3}	1.341×10^{-2}
Monomeric, Horizontal, DLPC, NBFIX, 85 x 85 Å, 310 K				
Restrained	1	7.675	6.515×10^{-3}	1.303×10^{-2}
	2	7.353	6.800×10^{-3}	1.360×10^{-2}
	3	7.558	6.616×10^{-3}	1.323×10^{-2}
	4	7.274	6.874×10^{-3}	1.375×10^{-2}

System COM-Restraint	Replica	L_z (nm)	E, ± 50 mV (V nm^{-1})	E, ± 100 mV (V nm^{-1})
	5	7.564	6.610 x 10^{-3}	1.322 x 10^{-2}
	6	7.388	6.768 x 10^{-3}	1.354 x 10^{-2}
Unrestrained	1	7.509	6.658 x 10^{-3}	1.332 x 10^{-2}
	2	7.615	6.566 x 10^{-3}	1.313 x 10^{-2}
	3	7.441	6.720 x 10^{-3}	1.344 x 10^{-2}
	4	7.545	6.627 x 10^{-3}	1.325 x 10^{-2}
	5	7.466	6.697 x 10^{-3}	1.339 x 10^{-2}
	6	7.434	6.726 x 10^{-3}	1.345 x 10^{-2}
Empty DLPC membrane patch, NBFIX, 85 x 85 Å, 310 K				
-	1	7.848	6.371 x 10^{-3}	1.274 x 10^{-2}
	2	7.878	6.347 x 10^{-3}	1.269 x 10^{-2}
	3	7.754	6.448 x 10^{-3}	1.290 x 10^{-2}
	4	7.774	6.432 x 10^{-3}	1.286 x 10^{-2}
	5	7.866	6.357 x 10^{-3}	1.271 x 10^{-2}
	6	7.866	6.356 x 10^{-3}	1.271 x 10^{-2}
Monomeric, Horizontal, DMPC, NBFIX, 85 x 85 Å, 310 K				
Restrained	1	8.101	6.172 x 10^{-3}	1.234 x 10^{-2}
	2	8.080	6.188 x 10^{-3}	1.238 x 10^{-2}
	3	7.932	6.303 x 10^{-3}	1.261 x 10^{-2}

System COM-Restraint	System Replica	L_z (nm)	E, ± 50 mV ($V\ nm^{-1}$)	E, ± 100 mV ($V\ nm^{-1}$)
	4	8.066	6.199×10^{-3}	1.240×10^{-2}
	5	8.036	6.222×10^{-3}	1.244×10^{-2}
	6	8.011	6.241×10^{-3}	1.248×10^{-2}
Unrestrained	1	7.805	6.406×10^{-3}	1.281×10^{-2}
	2	8.011	6.241×10^{-3}	1.248×10^{-2}
	3	7.873	6.351×10^{-3}	1.270×10^{-2}
	4	8.399	5.953×10^{-3}	1.191×10^{-2}
	5	8.070	6.196×10^{-3}	1.239×10^{-2}
	6	7.926	6.308×10^{-3}	1.262×10^{-2}
Empty DMPC membrane patch, NBFIX, 85 x 85 Å, 310 K				
-	1	8.237	6.071×10^{-3}	1.214×10^{-2}
	2	8.375	5.970×10^{-3}	1.194×10^{-2}
	3	8.111	6.164×10^{-3}	1.233×10^{-2}
	4	8.454	5.914×10^{-3}	1.183×10^{-2}
	5	8.771	5.700×10^{-3}	1.140×10^{-2}
	6	8.202	6.096×10^{-3}	1.219×10^{-2}
Dimeric, Horizontal, POPC:cholesterol (2:1), NBFIX, 85 x 85 Å, 293 K				
Restrained	1	9.402	5.318×10^{-3}	1.064×10^{-2}
	2	9.392	5.324×10^{-3}	1.065×10^{-2}

System		L_z (nm)	E, ± 50 mV (V nm^{-1})	E, ± 100 mV (V nm^{-1})
COM-Restraint	Replica			
	3	9.263	5.398 x 10^{-3}	1.080 x 10^{-2}
	4	9.243	5.410 x 10^{-3}	1.082 x 10^{-2}
	5	9.369	5.337 x 10^{-3}	1.067 x 10^{-2}
	6	9.361	5.341 x 10^{-3}	1.068 x 10^{-2}
Monomeric, Horizontal, POPC:cholesterol (2:1), NBFIX, 150 x 150 Å, 293 K				
Restrained	1	16.186	3.089 x 10^{-3}	6.178 x 10^{-3}
	2	16.166	3.093 x 10^{-3}	6.186 x 10^{-3}
	3	12.277	3.072 x 10^{-3}	6.144 x 10^{-3}
	4	16.216	3.083 x 10^{-3}	6.167 x 10^{-3}
	5	16.459	3.038 x 10^{-3}	6.076 x 10^{-3}
	6	16.408	3.047 x 10^{-3}	6.095 x 10^{-3}
Unrestrained	1	16.222	3.082 x 10^{-3}	6.165 x 10^{-3}
	2	16.321	3.064 x 10^{-3}	6.127 x 10^{-3}
	3	16.313	3.065 x 10^{-3}	6.130 x 10^{-3}
	4	16.239	3.079 x 10^{-3}	6.158 x 10^{-3}
	5	16.294	3.069 x 10^{-3}	6.137 x 10^{-3}
	6	16.346	3.059 x 10^{-3}	6.118 x 10^{-3}
Empty POPC:cholesterol (2:1) membrane patch, NBFIX, 150 x 150 Å, 293 K				

System		L_z (nm)	E, ± 50 mV (V nm^{-1})	E, ± 100 mV (V nm^{-1})
COM-Restraint	Replica			
-	1	17.008	2.940 x 10^{-3}	5.880 x 10^{-3}
	2	16.911	2.957 x 10^{-3}	5.913 x 10^{-3}
	3	17.204	2.906 x 10^{-3}	5.813 x 10^{-3}
	4	17.243	2.900 x 10^{-3}	5.799 x 10^{-3}
	5	17.116	2.921 x 10^{-3}	5.843 x 10^{-3}
	6	17.155	2.915 x 10^{-3}	5.829 x 10^{-3}

Table S13. Instantaneous restraint forces for COM-restrained simulations.

Transmembrane potential (mV)	Mean restraint force Fz (pN)	|qE| (pN)
Monomeric, Horizontal, POPC:cholesterol (2:1), NBFIX, 85 x 85 Å, 293 K		
-100	-38.50 ± 29.10	21.99
-50	-42.42 ± 32.64	11.00
+50	-35.87 ± 27.98	11.00
+100	-26.53 ± 47.91	21.99
Monomeric, Horizontal, POPC:cholesterol (2:1), prosECCo75 (all charges scaled), 85 x 85 Å, 293 K		
+100	-39.95 ± 22.07	15.71
Monomeric, Horizontal, POPC:cholesterol (2:1), prosECCo75 (only Fe(II) scaled), 85 x 85 Å, 293 K		
+100	-26.02 ± 18.34	16.16
Monomeric, Horizontal, DDPC, NBFIX, 85 x 85 Å, 310 K		
-100	-1.78 ± 33.47	27.72
-50	1.73 ± 31.58	13.86
+50	2.58 ± 24.36	13.86
+100	-0.40 ± 21.09	27.72
Monomeric, Horizontal, DCPC, NBFIX, 85 x 85 Å, 310 K		
-100	-13.23 ± 17.61	26.75
-50	-8.87 ± 17.96	13.38
+50	-8.33 ± 20.21	13.38
+100	-14.01 ± 17.16	26.75
Monomeric, Horizontal, DLPC, NBFIX, 85 x 85 Å, 310 K		
-100	-24.09 ± 34.19	25.75
-50	-25.70 ± 36.43	12.88
+50	-23.56 ± 35.66	12.88

+100	-30.76 ± 29.04	25.75

Monomeric, Horizontal, DMPC, NBFIX, 85 x 85 Å, 310 K

-100	18.31 ± 12.86	23.92
-50	20.36 ± 16.90	11.96
+50	13.91 ± 11.72	11.96
+100	7.039 ± 10.53	23.92

Monomeric, Horizontal, POPC:cholesterol (2:1), NBFIX, 150 x 150 Å, 293 K

-100	-9.30 ± 19.24	11.81
-50	-0.74 ± 25.14	5.90
+50	-11.97 ± 22.64	5.90
+100	-15.73 ± 31.38	11.81

S3 Data

S3.1 Conductance data

Table S14. Conductance data for all simulations.

COM restraint	Replica	Potential (mV)	Net permeation events		Gross ion transfer	Mean block conductance (nS)	Block SEM (nS)	95% CI (nS)	I-V conductance (nS)
			Cl^-	K^+					
**A. Monomeric, horizontal, POPC:cholesterol (2:1), NBFIX, 85 × 85 Å, 293 K**									
Restrained	1	−100	−352	0	354	2.26	0.14	1.87 – 2.64	2.05 ± 0.03
		−50	−168	0	174	2.15	0.26	1.43 – 2.88	
		+50	172	0	188	2.20	0.13	1.85 – 2.56	
		+100	325	0	327	2.08	0.11	1.79 – 2.38	
	2	−100	−281	0	281	1.80	0.10	1.53 – 2.07	
		−50	−148	0	148	1.90	0.21	1.31 – 2.49	
		+50	154	0	162	1.97	0.11	1.66 – 2.28	
		+100	303	0	303	1.94	0.15	1.52 – 2.36	
	3	−100	−323	0	323	2.07	0.16	1.62 – 2.52	
		−50	−123	0	127	1.58	0.13	1.22 – 1.94	
		+50	143	0	161	1.83	0.09	1.60 – 2.07	
		+100	324	0	326	2.08	0.27	1.33 – 2.82	
	4	−100	−333	0	333	2.13	0.11	1.82 – 2.45	
		−50	−168	0	180	2.15	0.05	2.01 – 2.30	
		+50	173	0	185	2.22	0.20	1.67 – 2.76	
		+100	347	0	349	2.22	0.08	1.99 – 2.45	
	5	−100	−332	1	333	2.13	0.06	1.96 – 2.31	
		−50	−149	0	169	1.91	0.22	1.31 – 2.51	
		+50	148	0	166	1.90	0.13	1.54 – 2.25	

COM restraint	Replica	Potential (mV)	Net permeation events		Gross ion transfer	Mean block conductance (nS)	Block SEM (nS)	95% CI (nS)	I-V conductance (nS)
			Cl^-	K^+					
		+100	325	0	325	2.08	0.14	1.70 - 2.47	
	6	−100	−342	0	342	2.19	0.20	1.63 - 2.75	
		−50	−146	0	156	1.87	0.16	1.43 - 2.31	
		+50	139	0	147	1.78	0.12	1.45 - 2.12	
		+100	286	0	286	1.83	0.09	1.58 - 2.09	
Unrestrained	1	−100	−288	0	290	1.85	0.18	1.36 - 2.33	1.34 ± 0.14
		−50	−140	0	150	1.79	0.10	1.51 - 2.08	
		+50	112	0	122	1.44	0.14	1.05 - 1.82	
		+100	259	0	261	1.66	0.08	1.43 - 1.89	
	2	−100	−316	0	316	2.03	0.08	1.81 - 2.24	
		−50	−111	0	125	1.42	0.17	0.94 - 1.90	
		+50	141	0	153	1.81	0.17	1.33 - 2.29	
		+100	328	0	330	2.10	0.10	1.83 - 2.37	
	3	−100	−320	0	320	2.05	0.09	1.79 - 2.31	
		−50	−135	0	149	1.73	0.17	1.27 - 2.19	
		+50	180	0	194	2.31	0.13	1.94 - 2.68	
		+100	310	0	312	1.99	0.07	1.78 - 2.19	
	4	−100	−32	−1	35	0.20	0.05	0.06 - 0.34	

COM restraint	Replica	Potential (mV)	Net permeation events		Gross ion transfer	Mean block conductance (nS)	Block SEM (nS)	95% CI (nS)	I-V conductance (nS)
			Cl^-	K^+					
		−50	−24	0	28	0.31	0.12	−0.04 – 0.65	
		+50	18	0	20	0.23	0.06	0.06 – 0.41	
		+100	59	0	59	0.38	0.04	0.25 – 0.50	
	5	−100	−184	0	184	1.18	0.08	0.95 – 1.40	
		−50	−88	0	98	1.13	0.22	0.52 – 1.74	
		+50	92	0	118	1.18	0.20	0.63 – 1.73	
		+100	183	0	185	1.17	0.08	0.94 – 1.41	
	6	−100	−156	0	158	1.00	0.17	0.53 – 1.46	
		−50	−75	0	83	0.96	0.19	0.42 – 1.50	
		+50	68	0	84	0.87	0.14	0.49 – 1.26	
		+100	121	0	123	0.78	0.03	0.69 – 0.86	
**B. Empty POPC:cholesterol (2:1) membrane patch, NBFIX, 85 × 85 Å, 293 K**									
–	1	−100	0	0	0	0.00	0.00	0.00 – 0.00	0.00 ± 0.00
		−50	0	0	0	0.00	0.00	0.00 – 0.00	
		+50	0	0	0	0.00	0.00	0.00 – 0.00	
		+100	0	0	0	0.00	0.00	0.00 – 0.00	
	2	−100	0	0	0	0.00	0.00	0.00 – 0.00	
		−50	0	0	0	0.00	0.00	0.00 – 0.00	

COM restraint	Replica	Potential (mV)	Net permeation events		Gross ion transfer	Mean block conductance (nS)	Block SEM (nS)	95% CI (nS)	I-V conductance (nS)
			Cl^-	K^+					
		+50	0	0	0	0.00	0.00	0.00 – 0.00	
		+100	0	0	0	0.00	0.00	0.00 – 0.00	
	3	−100	0	0	0	0.00	0.00	0.00 – 0.00	
		−50	0	0	0	0.00	0.00	0.00 – 0.00	
		+50	0	0	0	0.00	0.00	0.00 – 0.00	
		+100	0	0	0	0.00	0.00	0.00 – 0.00	
	4	−100	0	0	0	0.00	0.00	0.00 – 0.00	
		−50	0	0	0	0.00	0.00	0.00 – 0.00	
		+50	0	0	0	0.00	0.00	0.00 – 0.00	
		+100	0	0	0	0.00	0.00	0.00 – 0.00	
	5	−100	0	0	0	0.00	0.00	0.00 – 0.00	
		−50	0	0	0	0.00	0.00	0.00 – 0.00	
		+50	0	0	0	0.00	0.00	0.00 – 0.00	
		+100	0	0	0	0.00	0.00	0.00 – 0.00	
	6	−100	0	0	0	0.00	0.00	0.00 – 0.00	
		−50	0	0	0	0.00	0.00	0.00 – 0.00	
		+50	0	0	0	0.00	0.00	0.00 – 0.00	
		+100	0	0	0	0.00	0.00	0.00 – 0.00	

COM restraint	Replica	Potential (mV)	Net permeation events		Gross ion transfer	Mean block conductance (nS)	Block SEM (nS)	95% CI (nS)	I-V conductance (nS)
			Cl⁻	K⁺					
**C. Monomeric, horizontal, POPC:cholesterol (2:1), prosECCo75 (all charges scaled), 85 × 85 Å, 293 K**									
Restrained	1	+100	81	0	81	0.52	0.07	0.31 – 0.72	0.69 ± 0.18†
	2	+100	112	0	114	0.72	0.09	0.47 – 0.97	
	3	+100	144	0	144	0.92	0.10	0.65 – 1.19	
	4	+100	109	0	113	0.70	0.17	0.22 – 1.18	
	5	+100	130	0	134	0.83	0.14	0.44 – 1.22	
	6	+100	71	0	73	0.46	0.08	0.24 – 0.67	
Unrestrained	1	+100	23	0	25	0.15	0.06	−0.01 – 0.31	0.26 ± 0.34†
	2	+100	125	0	127	0.80	0.04	0.68 – 0.92	
	3	+100	87	−1	94	0.56	0.08	0.35 – 0.78	
	4	+100	0	0	0	0.00	0.00	0.00 – 0.00	
	5	+100	3	0	3	0.02	0.01	0.00 – 0.04	
	6	+100	0	0	0	0.00	0.00	0.00 – 0.00	
**D. Monomeric, horizontal, POPC:cholesterol (2:1), prosECCo75 (Fe(II) charges only), 85 × 85 Å, 293 K**									
COM-Restrained	1	+100	77	0	79	0.49	0.04	0.37 – 0.61	0.54 ± 0.02†
	2	+100	49	0	51	0.31	0.04	0.21 – 0.41	
	3	+100	90	0	90	0.58	0.10	0.30 – 0.86	
	4	+100	77	0	77	0.49	0.07	0.29 – 0.69	
	5	+100	102	0	102	0.65	0.08	0.44 – 0.87	

COM restraint	Replica	Potential (mV)	Net permeation events		Gross ion transfer	Mean block conductance (nS)	Block SEM (nS)	95% CI (nS)	I-V conductance (nS)
			Cl^-	K^+					
	6	+100	83	0	85	0.53	0.05	0.39 – 0.67	
COM-Unrestrained	1	+100	2	0	2	0.01	0.01	−0.02 – 0.05	0.26 ± 0.03†
	2	+100	46	0	46	0.29	0.04	0.17 – 0.42	
	3	+100	49	0	49	0.31	0.04	0.20 – 0.43	
	4	+100	62	0	62	0.40	0.04	0.29 – 0.50	
	5	+100	79	0	81	0.51	0.10	0.23 – 0.78	
	6	+100	21	0	21	0.13	0.04	0.01 – 0.26	
**E. Monomeric, horizontal, POPC:cholesterol (2:1), NBFIX, dihedral restraints, 85 × 85 Å, 293 K**									
Restrained	1	−100	−59	0	59	0.38	0.05	0.25 – 0.51	0.68 ± 0.05
		−50	−40	0	44	0.51	0.12	0.17 – 0.86	
		+50	55	0	55	0.70	0.09	0.45 – 0.96	
		+100	65	0	65	0.42	0.08	0.21 – 0.63	
	2	−100	−120	0	120	0.77	0.09	0.52 – 1.02	
		−50	−72	0	72	0.92	0.08	0.69 – 1.15	
		+50	50	0	50	0.64	0.11	0.32 – 0.96	
		+100	131	0	131	0.84	0.06	0.67 – 1.00	
	3	−100	−33	0	33	0.21	0.05	0.06 – 0.36	
		−50	−38	0	38	0.49	0.06	0.33 – 0.64	

COM restraint	Replica	Potential (mV)	Net permeation events		Gross ion transfer	Mean block conductance (nS)	Block SEM (nS)	95% CI (nS)	I-V conductance (nS)
			Cl^-	K^+					
		+50	46	0	48	0.59	0.08	0.37 – 0.81	
		+100	92	0	92	0.59	0.09	0.33 – 0.85	
	4	−100	−115	0	115	0.74	0.11	0.42 – 1.05	
		−50	−42	0	46	0.54	0.05	0.39 – 0.68	
		+50	29	0	35	0.37	0.08	0.14 – 0.61	
		+100	116	0	116	0.74	0.09	0.49 – 1.00	
	5	−100	−109	0	109	0.70	0.05	0.55 – 0.85	
		−50	−81	0	87	1.04	0.18	0.53 – 1.55	
		+50	62	0	62	0.79	0.13	0.43 – 1.16	
		+100	92	0	92	0.59	0.11	0.28 – 0.90	
	6	−100	−184	0	184	1.18	0.08	0.96 – 1.40	
		−50	−42	0	52	0.54	0.15	0.12 – 0.96	
		+50	70	0	70	0.90	0.08	0.67 – 1.13	
		+100	173	0	173	1.11	0.13	0.75 – 1.47	
Unrestrained	1	−100	−35	0	35	0.22	0.07	0.03 – 0.42	0.45 ± 0.05
		−50	−17	0	19	0.22	0.09	−0.03 – 0.47	
		+50	11	0	11	0.14	0.07	−0.04 – 0.32	
		+100	30	0	30	0.19	0.05	0.05 – 0.34	

COM restraint	Replica	Potential (mV)	Net permeation events		Gross ion transfer	Mean block conductance (nS)	Block SEM (nS)	95% CI (nS)	I-V conductance (nS)
			Cl⁻	K⁺					
	2	−100	−114	0	114	0.73	0.18	0.23 – 1.23	
		−50	−65	0	67	0.83	0.16	0.39 – 1.27	
		+50	50	0	50	0.64	0.11	0.33 – 0.95	
		+100	115	0	115	0.74	0.04	0.62 – 0.85	
	3	−100	−58	0	58	0.37	0.06	0.21 – 0.54	
		−50	−60	0	60	0.77	0.17	0.31 – 1.23	
		+50	29	0	31	0.37	0.09	0.12 – 0.63	
		+100	36	0	38	0.23	0.04	0.12 – 0.34	
	4	−100	−81	0	81	0.52	0.13	0.16 – 0.88	
		−50	−31	0	35	0.40	0.02	0.33 – 0.46	
		+50	34	0	34	0.44	0.07	0.23 – 0.64	
		+100	55	0	55	0.35	0.10	0.08 – 0.62	
	5	−100	−122	0	122	0.78	0.03	0.71 – 0.85	
		−50	−68	0	70	0.87	0.12	0.54 – 1.20	
		+50	54	0	54	0.69	0.12	0.35 – 1.04	
		+100	132	0	132	0.85	0.17	0.38 – 1.31	
	6	−100	−31	0	31	0.20	0.04	0.08 – 0.31	
		−50	−17	0	17	0.22	0.07	0.03 – 0.41	

COM restraint	Replica	Potential (mV)	Net permeation events		Gross ion transfer	Mean block conductance (nS)	Block SEM (nS)	95% CI (nS)	I-V conductance (nS)
			Cl^-	K^+					
		+50	14	0	14	0.18	0.06	0.01 – 0.35	
		+100	17	0	17	0.11	0.02	0.04 – 0.18	
F. Monomeric, horizontal, DDPC, NBFIX, 85 × 85 Å, 310 K									
	1	−100	−669	0	675	4.29	0.12	3.96 – 4.61	
		−50	−310	0	338	3.97	0.37	2.94 – 5.01	
		+50	312	0	352	4.00	0.56	2.44 – 5.56	
		+100	590	0	594	3.78	0.18	3.29 – 4.27	
	2	−100	−592	0	596	3.79	0.21	3.22 – 4.37	
		−50	−304	1	341	3.91	0.21	3.32 – 4.50	
		+50	299	0	327	3.83	0.46	2.55 – 5.12	
Restrained		+100	566	0	570	3.63	0.17	3.15 – 4.10	3.86 ± 0.04
	3	−100	−561	0	565	3.60	0.17	3.12 – 4.07	
		−50	−258	0	304	3.31	0.17	2.83 – 3.79	
		+50	289	1	328	3.69	0.18	3.19 – 4.19	
		+100	585	0	593	3.75	0.18	3.24 – 4.26	
	4	−100	−606	0	614	3.88	0.12	3.56 – 4.21	
		−50	−303	1	338	3.90	0.06	3.74 – 4.05	
		+50	308	0	338	3.95	0.26	3.24 – 4.66	

COM restraint	Replica	Potential (mV)	Net permeation events		Gross ion transfer	Mean block conductance (nS)	Block SEM (nS)	95% CI (nS)	I-V conductance (nS)
			Cl⁻	K⁺					
		+100	585	0	591	3.75	0.10	3.46 – 4.04	
	5	−100	−660	0	666	4.23	0.08	4.01 – 4.45	
		−50	−319	0	357	4.09	0.18	3.59 – 4.58	
		+50	313	0	353	4.01	0.14	3.64 – 4.39	
		+100	581	0	583	3.72	0.22	3.11 – 4.34	
	6	−100	−619	1	624	3.97	0.14	3.57 – 4.37	
		−50	−306	0	342	3.92	0.24	3.26 – 4.59	
		+50	285	0	317	3.65	0.16	3.20 – 4.10	
		+100	608	0	608	3.90	0.07	3.72 – 4.08	
Unrestrained	1	−100	−544	0	546	3.49	0.16	3.05 – 3.92	3.96 ± 0.05
		−50	−289	0	347	3.70	0.18	3.20 – 4.21	
		+50	279	0	319	3.58	0.23	2.95 – 4.21	
		+100	644	−1	647	4.13	0.23	3.49 – 4.77	
	2	−100	−601	0	603	3.85	0.14	3.48 – 4.23	
		−50	−308	0	336	3.95	0.19	3.41 – 4.48	
		+50	277	1	328	3.54	0.27	2.78 – 4.30	
		+100	591	0	593	3.79	0.16	3.34 – 4.23	
	3	−100	−594	1	597	3.81	0.12	3.49 – 4.14	

COM restraint	Replica	Potential (mV)	Net permeation events		Gross ion transfer	Mean block conductance (nS)	Block SEM (nS)	95% CI (nS)	I-V conductance (nS)
			Cl^-	K^+					
		−50	−302	0	332	3.87	0.14	3.48 – 4.26	
		+50	315	0	335	4.04	0.19	3.51 – 4.56	
		+100	612	0	622	3.92	0.12	3.59 – 4.25	
	4	−100	−607	0	611	3.89	0.11	3.58 – 4.20	
		−50	−319	0	355	4.09	0.30	3.24 – 4.94	
		+50	321	0	357	4.11	0.31	3.26 – 4.97	
		+100	641	0	641	4.11	0.16	3.68 – 4.54	
	5	−100	−650	0	656	4.17	0.16	3.73 – 4.60	
		−50	−306	0	350	3.92	0.16	3.47 – 4.37	
		+50	336	1	357	4.29	0.21	3.71 – 4.88	
		+100	602	−1	605	3.86	0.11	3.55 – 4.18	
	6	−100	−644	0	648	4.13	0.29	3.31 – 4.94	
		−50	−340	0	360	4.36	0.15	3.94 – 4.78	
		+50	344	0	392	4.41	0.34	3.48 – 5.34	
		+100	657	0	661	4.21	0.21	3.63 – 4.79	
**G. Empty DDPC membrane patch, NBFIX, 85 × 85 Å, 310 K**									
-	1	−100	0	0	0	0.00	0.00	0.00 – 0.00	0.00 ± 0.00
		−50	0	0	0	0.00	0.00	0.00 – 0.00	

COM restraint	Replica	Potential (mV)	Net permeation events		Gross ion transfer	Mean block conductance (nS)	Block SEM (nS)	95% CI (nS)	I-V conductance (nS)
			Cl^-	K^+					
		+50	0	0	0	0.00	0.00	0.00 – 0.00	
		+100	0	0	0	0.00	0.00	0.00 – 0.00	
	2	−100	0	0	0	0.00	0.00	0.00 – 0.00	
		−50	0	0	0	0.00	0.00	0.00 – 0.00	
		+50	0	0	0	0.00	0.00	0.00 – 0.00	
		+100	0	0	0	0.00	0.00	0.00 – 0.00	
	3	−100	0	0	0	0.00	0.00	0.00 – 0.00	
		−50	0	0	0	0.00	0.00	0.00 – 0.00	
		+50	0	0	0	0.00	0.00	0.00 – 0.00	
		+100	0	0	0	0.00	0.00	0.00 – 0.00	
	4	−100	0	0	0	0.00	0.00	0.00 – 0.00	
		−50	0	0	0	0.00	0.00	0.00 – 0.00	
		+50	0	0	0	0.00	0.00	0.00 – 0.00	
		+100	0	0	0	0.00	0.00	0.00 – 0.00	
	5	−100	0	0	0	0.00	0.00	0.00 – 0.00	
		−50	0	0	0	0.00	0.00	0.00 – 0.00	
		+50	0	0	0	0.00	0.00	0.00 – 0.00	
		+100	0	0	0	0.00	0.00	0.00 – 0.00	

COM restraint	Replica	Potential (mV)	Net permeation events		Gross ion transfer	Mean block conductance (nS)	Block SEM (nS)	95% CI (nS)	I-V conductance (nS)
			Cl^-	K^+					
	6	−100	0	0	0	0.00	0.00	0.00 – 0.00	
		−50	0	0	0	0.00	0.00	0.00 – 0.00	
		+50	0	0	0	0.00	0.00	0.00 – 0.00	
		+100	0	−1	1	0.01	0.01	−0.01 – 0.02	
**H. Monomeric, horizontal, DCPC, NBFIX, 85 × 85 Å, 310 K**									
Restrained	1	−100	−586	0	588	3.76	0.13	3.39 – 4.12	3.79 ± 0.05
		−50	−283	0	321	3.63	0.20	3.06 – 4.19	
		+50	289	0	321	3.70	0.19	3.19 – 4.22	
		+100	583	0	589	3.74	0.14	3.34 – 4.13	
	2	−100	−569	0	583	3.65	0.05	3.51 – 3.79	
		−50	−306	0	346	3.92	0.15	3.52 – 4.33	
		+50	260	0	300	3.33	0.16	2.90 – 3.76	
		+100	537	0	537	3.44	0.11	3.13 – 3.76	
	3	−100	−660	1	667	4.24	0.22	3.64 – 4.84	
		−50	−302	0	342	3.87	0.21	3.28 – 4.46	
		+50	287	0	327	3.68	0.29	2.88 – 4.48	
		+100	633	0	635	4.06	0.15	3.63 – 4.48	
	4	−100	−537	0	539	3.44	0.14	3.07 – 3.82	

COM restraint	Replica	Potential (mV)	Net permeation events		Gross ion transfer	Mean block conductance (nS)	Block SEM (nS)	95% CI (nS)	I-V conductance (nS)
			Cl^-	K^+					
		−50	−276	0	314	3.54	0.12	3.21 – 3.86	
		+50	299	0	333	3.83	0.17	3.36 – 4.30	
		+100	594	0	596	3.81	0.19	3.29 – 4.32	
	5	−100	−602	0	608	3.86	0.20	3.31 – 4.41	
		−50	−262	0	306	3.36	0.24	2.71 – 4.01	
		+50	300	0	342	3.85	0.32	2.95 – 4.74	
		+100	626	0	634	4.01	0.07	3.82 – 4.21	
	6	−100	−582	0	588	3.73	0.12	3.39 – 4.07	
		−50	−305	0	333	3.91	0.24	3.23 – 4.58	
		+50	288	0	332	3.69	0.30	2.85 – 4.54	
		+100	624	0	626	4.00	0.10	3.71 – 4.29	
Unrestrained	1	−100	−596	0	596	3.82	0.13	3.47 – 4.17	3.78 ± 0.06
		−50	−276	0	308	3.54	0.14	3.16 – 3.91	
		+50	303	−1	334	3.91	0.26	3.18 – 4.63	
		+100	582	0	582	3.73	0.18	3.24 – 4.22	
	2	−100	−601	1	612	3.86	0.18	3.37 – 4.34	
		−50	−304	−1	337	3.88	0.26	3.18 – 4.59	
		+50	296	0	340	3.79	0.24	3.12 – 4.46	

COM restraint	Replica	Potential (mV)	Net permeation events		Gross ion transfer	Mean block conductance (nS)	Block SEM (nS)	95% CI (nS)	I-V conductance (nS)
			Cl^-	K^+					
		+100	577	−1	580	3.70	0.28	2.93 – 4.48	
	3	−100	−549	0	557	3.52	0.22	2.90 – 4.13	
		−50	−298	0	338	3.82	0.10	3.53 – 4.10	
		+50	250	0	300	3.20	0.16	2.76 – 3.65	
		+100	556	0	558	3.56	0.19	3.04 – 4.08	
	4	−100	−648	0	658	4.15	0.13	3.79 – 4.51	
		−50	−326	0	364	4.18	0.29	3.38 – 4.98	
		+50	305	0	319	3.91	0.09	3.67 – 4.15	
		+100	634	0	646	4.06	0.20	3.52 – 4.61	
	5	−100	−611	0	619	3.92	0.05	3.79 – 4.05	
		−50	−338	0	372	4.33	0.22	3.71 – 4.96	
		+50	302	0	346	3.87	0.28	3.10 – 4.64	
		+100	646	0	654	4.14	0.08	3.91 – 4.37	
	6	−100	−504	0	510	3.23	0.11	2.93 – 3.53	
		−50	−256	0	304	3.28	0.30	2.45 – 4.11	
		+50	276	−1	309	3.55	0.19	3.03 – 4.07	
		+100	582	0	584	3.73	0.09	3.47 – 3.99	
**I. Empty DCPC membrane patch, NBFIX, 85 × 85 Å, 310 K**									

COM restraint	Replica	Potential (mV)	Net permeation events		Gross ion transfer	Mean block conductance (nS)	Block SEM (nS)	95% CI (nS)	I-V conductance (nS)
			Cl^-	K^+					
–	1	−100	0	0	0	0.00	0.00	0.00 – 0.00	0.00 ± 0.00
		−50	0	0	0	0.00	0.00	0.00 – 0.00	
		+50	0	0	0	0.00	0.00	0.00 – 0.00	
		+100	0	0	0	0.00	0.00	0.00 – 0.00	
	2	−100	0	0	0	0.00	0.00	0.00 – 0.00	
		−50	0	0	0	0.00	0.00	0.00 – 0.00	
		+50	0	0	0	0.00	0.00	0.00 – 0.00	
		+100	0	0	0	0.00	0.00	0.00 – 0.00	
	3	−100	0	0	0	0.00	0.00	0.00 – 0.00	
		−50	0	0	0	0.00	0.00	0.00 – 0.00	
		+50	0	0	0	0.00	0.00	0.00 – 0.00	
		+100	0	0	0	0.00	0.00	0.00 – 0.00	
	4	−100	0	0	0	0.00	0.00	0.00 – 0.00	
		−50	0	0	0	0.00	0.00	0.00 – 0.00	
		+50	0	0	0	0.00	0.00	0.00 – 0.00	
		+100	0	0	0	0.00	0.00	0.00 – 0.00	
	5	−100	0	0	0	0.00	0.00	0.00 – 0.00	
		−50	0	0	0	0.00	0.00	0.00 – 0.00	

COM restraint	Replica	Potential (mV)	Net permeation events		Gross ion transfer	Mean block conductance (nS)	Block SEM (nS)	95% CI (nS)	I-V conductance (nS)
			Cl^-	K^+					
		+50	0	0	0	0.00	0.00	0.00 – 0.00	
		+100	0	0	0	0.00	0.00	0.00 – 0.00	
	6	−100	0	0	0	0.00	0.00	0.00 – 0.00	
		−50	0	0	0	0.00	0.00	0.00 – 0.00	
		+50	0	0	0	0.00	0.00	0.00 – 0.00	
		+100	0	0	0	0.00	0.00	0.00 – 0.00	
**J. Monomeric, horizontal, DLPC, NBFIX, 85 × 85 Å, 310 K**									
Restrained	1	−100	−477	0	485	3.06	0.21	2.49 – 3.63	3.46 ± 0.08
		−50	−268	0	290	3.44	0.21	2.84 – 4.03	
		+50	255	0	265	3.27	0.20	2.71 – 3.82	
		+100	498	0	500	3.19	0.11	2.90 – 3.49	
	2	−100	−545	−1	556	3.49	0.12	3.16 – 3.81	
		−50	−341	0	377	4.37	0.28	3.59 – 5.15	
		+50	278	0	314	3.56	0.23	2.93 – 4.20	
		+100	527	0	527	3.38	0.09	3.13 – 3.62	
	3	−100	−504	0	504	3.23	0.12	2.91 – 3.55	
		−50	−241	0	275	3.09	0.17	2.61 – 3.56	
		+50	255	0	291	3.27	0.36	2.27 – 4.27	

COM restraint	Replica	Potential (mV)	Net permeation events		Gross ion transfer	Mean block conductance (nS)	Block SEM (nS)	95% CI (nS)	I-V conductance (nS)
			Cl^-	K^+					
		+100	518	0	518	3.32	0.06	3.16 – 3.48	
	4	−100	−617	0	621	3.95	0.16	3.51 – 4.40	
		−50	−263	0	319	3.37	0.18	2.87 – 3.88	
		+50	317	0	355	4.06	0.07	3.86 – 4.27	
		+100	685	0	685	4.39	0.14	4.01 – 4.77	
	5	−100	−511	0	515	3.27	0.14	2.89 – 3.66	
		−50	−272	1	291	3.50	0.25	2.80 – 4.20	
		+50	234	0	274	3.00	0.20	2.44 – 3.56	
		+100	500	0	502	3.20	0.08	2.97 – 3.43	
	6	−100	−543	0	545	3.48	0.06	3.31 – 3.65	
		−50	−260	0	294	3.33	0.12	2.99 – 3.68	
		+50	276	0	310	3.54	0.04	3.42 – 3.66	
		+100	535	0	539	3.43	0.12	3.10 – 3.76	
Unrestrained	1	−100	−485	0	491	3.11	0.13	2.76 – 3.46	3.42 ± 0.04
		−50	−298	0	336	3.82	0.15	3.41 – 4.23	
		+50	306	−1	339	3.94	0.19	3.40 – 4.47	
		+100	533	0	535	3.42	0.10	3.13 – 3.70	
	2	−100	−539	0	541	3.45	0.19	2.94 – 3.97	

COM restraint	Replica	Potential (mV)	Net permeation events		Gross ion transfer	Mean block conductance (nS)	Block SEM (nS)	95% CI (nS)	I-V conductance (nS)
			Cl^-	K^+					
		−50	−246	0	272	3.15	0.12	2.83 – 3.48	
		+50	240	0	266	3.08	0.30	2.24 – 3.92	
		+100	534	−1	547	3.43	0.22	2.81 – 4.04	
	3	−100	−496	0	510	3.18	0.13	2.83 – 3.53	
		−50	−280	0	308	3.59	0.12	3.24 – 3.94	
		+50	275	0	301	3.52	0.15	3.12 – 3.93	
		+100	598	0	606	3.83	0.13	3.47 – 4.20	
	4	−100	−541	0	547	3.47	0.13	3.10 – 3.84	
		−50	−243	0	263	3.11	0.13	2.77 – 3.46	
		+50	264	0	296	3.38	0.35	2.40 – 4.36	
		+100	503	1	510	3.22	0.17	2.75 – 3.68	
	5	−100	−528	0	530	3.38	0.12	3.06 – 3.70	
		−50	−272	1	291	3.50	0.18	3.01 – 3.99	
		+50	257	−1	294	3.31	0.28	2.53 – 4.08	
		+100	549	0	551	3.52	0.20	2.96 – 4.08	
	6	−100	−550	0	558	3.52	0.09	3.28 – 3.77	
		−50	−296	0	328	3.79	0.27	3.06 – 4.53	
		+50	267	0	305	3.42	0.07	3.22 – 3.62	

COM restraint	Replica	Potential (mV)	Net permeation events		Gross ion transfer	Mean block conductance (nS)	Block SEM (nS)	95% CI (nS)	I-V conductance (nS)
			Cl^-	K^+					
		+100	526	0	528	3.37	0.20	2.81 – 3.93	
**K. Empty DLPC membrane patch, NBFIX, 85 × 85 Å, 310 K**									
-	1	−100	0	0	0	0.00	0.00	0.00 – 0.00	0.00 ± 0.00
		−50	0	0	0	0.00	0.00	0.00 – 0.00	
		+50	0	0	0	0.00	0.00	0.00 – 0.00	
		+100	0	0	0	0.00	0.00	0.00 – 0.00	
	2	−100	0	0	0	0.00	0.00	0.00 – 0.00	
		−50	0	0	0	0.00	0.00	0.00 – 0.00	
		+50	0	0	0	0.00	0.00	0.00 – 0.00	
		+100	0	0	0	0.00	0.00	0.00 – 0.00	
	3	−100	0	0	0	0.00	0.00	0.00 – 0.00	
		−50	0	0	0	0.00	0.00	0.00 – 0.00	
		+50	0	0	0	0.00	0.00	0.00 – 0.00	
		+100	0	0	0	0.00	0.00	0.00 – 0.00	
	4	−100	0	0	0	0.00	0.00	0.00 – 0.00	
		−50	0	0	0	0.00	0.00	0.00 – 0.00	
		+50	0	0	0	0.00	0.00	0.00 – 0.00	
		+100	0	0	0	0.00	0.00	0.00 – 0.00	

COM restraint	Replica	Potential (mV)	Net permeation events		Gross ion transfer	Mean block conductance (nS)	Block SEM (nS)	95% CI (nS)	I-V conductance (nS)
			Cl⁻	K⁺					
	5	−100	0	0	0	0.00	0.00	0.00 – 0.00	
		−50	0	0	0	0.00	0.00	0.00 – 0.00	
		+50	0	0	0	0.00	0.00	0.00 – 0.00	
		+100	0	0	0	0.00	0.00	0.00 – 0.00	
	6	−100	0	0	0	0.00	0.00	0.00 – 0.00	
		−50	0	0	0	0.00	0.00	0.00 – 0.00	
		+50	0	0	0	0.00	0.00	0.00 – 0.00	
		+100	0	0	0	0.00	0.00	0.00 – 0.00	
**L. Monomeric, horizontal, DMPC, NBFIX, 85 × 85 Å, 310 K**									
Restrained	1	−100	−468	0	474	3.00	0.16	2.56 – 3.44	2.95 ± 0.03
		−50	−234	0	266	3.00	0.25	2.31 – 3.69	
		+50	223	0	261	2.86	0.10	2.58 – 3.14	
		+100	470	0	472	3.01	0.12	2.68 – 3.35	
	2	−100	−444	0	452	2.85	0.18	2.35 – 3.34	
		−50	−224	0	252	2.87	0.13	2.52 – 3.22	
		+50	215	0	251	2.76	0.15	2.34 – 3.17	
		+100	445	0	449	2.85	0.12	2.52 – 3.18	
	3	−100	−445	0	451	2.85	0.11	2.54 – 3.17	

COM restraint	Replica	Potential (mV)	Net permeation events		Gross ion transfer	Mean block conductance (nS)	Block SEM (nS)	95% CI (nS)	I-V conductance (nS)
			Cl^-	K^+					
		−50	−256	0	292	3.28	0.24	2.62 – 3.94	
		+50	231	1	250	2.95	0.15	2.53 – 3.37	
		+100	498	0	498	3.19	0.06	3.02 – 3.36	
	4	−100	−417	0	417	2.67	0.12	2.35 – 3.00	
		−50	−208	0	234	2.67	0.28	1.90 – 3.43	
		+50	234	0	258	3.00	0.23	2.37 – 3.62	
		+100	469	0	471	3.01	0.11	2.69 – 3.32	
	5	−100	−472	0	474	3.02	0.15	2.61 – 3.44	
		−50	−228	0	248	2.92	0.29	2.12 – 3.73	
		+50	253	0	269	3.24	0.26	2.51 – 3.97	
		+100	436	0	440	2.79	0.04	2.69 – 2.90	
	6	−100	−481	0	483	3.08	0.20	2.52 – 3.64	
		−50	−215	0	257	2.76	0.22	2.15 – 3.36	
		+50	267	−1	310	3.44	0.28	2.67 – 4.20	
		+100	476	1	477	3.04	0.12	2.70 – 3.39	
Unrestrained	1	−100	−493	0	499	3.16	0.24	2.50 – 3.82	3.06 ± 0.06
		−50	−245	0	279	3.14	0.18	2.65 – 3.63	
		+50	257	0	291	3.29	0.06	3.14 – 3.45	

COM restraint	Replica	Potential (mV)	Net permeation events		Gross ion transfer	Mean block conductance (nS)	Block SEM (nS)	95% CI (nS)	I-V conductance (nS)
			Cl⁻	K⁺					
		+100	494	0	504	3.17	0.07	2.98 – 3.35	
	2	−100	−441	0	447	2.83	0.04	2.71 – 2.94	
		−50	−242	0	266	3.10	0.17	2.63 – 3.57	
		+50	264	0	292	3.38	0.21	2.79 – 3.98	
		+100	493	0	499	3.16	0.10	2.87 – 3.45	
	3	−100	−557	0	561	3.57	0.17	3.09 – 4.05	
		−50	−257	0	279	3.29	0.17	2.82 – 3.77	
		+50	255	0	289	3.27	0.22	2.65 – 3.89	
		+100	549	0	553	3.52	0.15	3.09 – 3.94	
	4	−100	−428	0	434	2.74	0.12	2.41 – 3.07	
		−50	−236	0	254	3.02	0.15	2.62 – 3.43	
		+50	229	−1	252	2.95	0.08	2.73 – 3.17	
		+100	412	0	416	2.64	0.15	2.23 – 3.05	
	5	−100	−469	0	475	3.01	0.13	2.65 – 3.36	
		−50	−244	0	262	3.13	0.08	2.90 – 3.36	
		+50	250	0	268	3.20	0.24	2.53 – 3.88	
		+100	435	0	439	2.79	0.09	2.53 – 3.04	
	6	−100	−474	1	479	3.04	0.24	2.37 – 3.72	

COM restraint	Replica	Potential (mV)	Net permeation events		Gross ion transfer	Mean block conductance (nS)	Block SEM (nS)	95% CI (nS)	I-V conductance (nS)
			Cl⁻	K⁺					
		−50	−222	0	254	2.85	0.16	2.39 – 3.30	
		+50	219	0	239	2.81	0.21	2.22 – 3.40	
		+100	452	0	454	2.90	0.08	2.68 – 3.11	
**M. Empty DMPC membrane patch, NBFIX, 85 × 85 Å, 310 K**									
–	1	−100	0	0	0	0.00	0.00	0.00 – 0.00	0.00 ± 0.00
		−50	0	0	0	0.00	0.00	0.00 – 0.00	
		+50	0	0	0	0.00	0.00	0.00 – 0.00	
		+100	0	0	0	0.00	0.00	0.00 – 0.00	
	2	−100	0	0	0	0.00	0.00	0.00 – 0.00	
		−50	0	0	0	0.00	0.00	0.00 – 0.00	
		+50	0	0	0	0.00	0.00	0.00 – 0.00	
		+100	0	0	0	0.00	0.00	0.00 – 0.00	
	3	−100	0	0	0	0.00	0.00	0.00 – 0.00	
		−50	0	0	0	0.00	0.00	0.00 – 0.00	
		+50	0	0	0	0.00	0.00	0.00 – 0.00	
		+100	0	0	0	0.00	0.00	0.00 – 0.00	
	4	−100	0	0	0	0.00	0.00	0.00 – 0.00	
		−50	0	0	0	0.00	0.00	0.00 – 0.00	

COM restraint	Replica	Potential (mV)	Net permeation events		Gross ion transfer	Mean block conductance (nS)	Block SEM (nS)	95% CI (nS)	I-V conductance (nS)
			Cl⁻	K⁺					
		+50	0	0	0	0.00	0.00	0.00 – 0.00	
		+100	0	0	0	0.00	0.00	0.00 – 0.00	
	5	−100	0	0	0	0.00	0.00	0.00 – 0.00	
		−50	0	0	0	0.00	0.00	0.00 – 0.00	
		+50	0	0	0	0.00	0.00	0.00 – 0.00	
		+100	0	0	0	0.00	0.00	0.00 – 0.00	
	6	−100	0	0	0	0.00	0.00	0.00 – 0.00	
		−50	0	0	0	0.00	0.00	0.00 – 0.00	
		+50	0	0	0	0.00	0.00	0.00 – 0.00	
		+100	0	0	0	0.00	0.00	0.00 – 0.00	
**N. Dimeric, horizontal, POPC:cholesterol (2:1), NBFIX, 85 × 85 Å, 293 K**									
Unrestrained	1	−100	−286	0	286	1.83	0.07	1.63 – 2.03	1.84 ± 0.02
		−50	−131	0	135	1.68	0.21	1.11 – 2.25	
		+50	135	0	137	1.73	0.09	1.49 – 1.98	
		+100	277	0	277	1.78	0.09	1.53 – 2.02	
	2	−100	−285	0	285	1.83	0.13	1.46 – 2.19	
		−50	−134	0	140	1.72	0.15	1.30 – 2.14	
		+50	132	0	134	1.69	0.21	1.12 – 2.27	

COM restraint	Replica	Potential (mV)	Net permeation events		Gross ion transfer	Mean block conductance (nS)	Block SEM (nS)	95% CI (nS)	I-V conductance (nS)
			Cl^-	K^+					
		+100	297	0	297	1.90	0.10	1.64 – 2.17	
	3	−100	−289	0	289	1.85	0.08	1.63 – 2.07	
		−50	−137	0	141	1.76	0.13	1.40 – 2.11	
		+50	156	0	162	2.00	0.31	1.14 – 2.86	
		+100	272	0	272	1.74	0.14	1.34 – 2.14	
	4	−100	−294	0	296	1.88	0.08	1.66 – 2.10	
		−50	−136	0	142	1.74	0.12	1.40 – 2.09	
		+50	161	0	165	2.06	0.17	1.59 – 2.53	
		+100	316	0	318	2.03	0.03	1.93 – 2.12	
	5	−100	−308	0	308	1.97	0.08	1.76 – 2.19	
		−50	−140	0	148	1.79	0.24	1.14 – 2.45	
		+50	136	0	142	1.74	0.31	0.88 – 2.61	
		+100	277	0	277	1.78	0.12	1.44 – 2.11	
	6	−100	−291	0	291	1.86	0.11	1.57 – 2.16	
		−50	−144	0	146	1.85	0.17	1.38 – 2.31	
		+50	151	0	155	1.94	0.25	1.24 – 2.63	
		+100	257	0	259	1.65	0.17	1.19 – 2.11	
**O. Monomeric, horizontal, POPC:cholesterol (2:1), NBFIX, 150 × 150 Å, 293 K**									

COM restraint	Replica	Potential (mV)	Net permeation events		Gross ion transfer	Mean block conductance (nS)	Block SEM (nS)	95% CI (nS)	I-V conductance (nS)
			Cl^-	K^+					
Restrained	1	−100	−325	0	327	2.08	0.10	1.81 - 2.36	2.06 ± 0.05
		−50	−170	0	182	2.18	0.18	1.67 - 2.69	
		+50	152	0	168	1.95	0.09	1.69 - 2.20	
		+100	400	0	402	2.56	0.10	2.30 - 2.83	
	2	−100	−335	0	337	2.15	0.05	2.00 - 2.29	
		−50	−164	0	186	2.10	0.17	1.63 - 2.57	
		+50	142	0	166	1.82	0.32	0.94 - 2.70	
		+100	301	−1	304	1.94	0.17	1.45 - 2.42	
	3	−100	−292	0	296	1.87	0.08	1.64 - 2.11	
		−50	−142	0	164	1.82	0.19	1.30 - 2.34	
		+50	146	0	172	1.87	0.13	1.50 - 2.24	
		+100	377	0	381	2.42	0.16	1.98 - 2.85	
	4	−100	−288	0	292	1.85	0.14	1.45 - 2.24	
		−50	−162	0	172	2.08	0.18	1.57 - 2.58	
		+50	158	0	168	2.03	0.24	1.36 - 2.69	
		+100	322	−1	327	2.07	0.12	1.74 - 2.40	
	5	−100	−324	1	327	2.08	0.07	1.88 - 2.29	
		−50	−146	0	152	1.87	0.16	1.43 - 2.31	

COM restraint	Replica	Potential (mV)	Net permeation events		Gross ion transfer	Mean block conductance (nS)	Block SEM (nS)	95% CI (nS)	I-V conductance (nS)
			Cl^-	K^+					
		+50	115	0	135	1.47	0.19	0.93 – 2.01	
		+100	282	−1	283	1.81	0.11	1.50 – 2.13	
	6	−100	−333	0	333	2.13	0.16	1.70 – 2.57	
		−50	−150	0	158	1.92	0.03	1.84 – 2.00	
		+50	162	0	170	2.08	0.16	1.65 – 2.51	
		+100	344	0	346	2.20	0.20	1.66 – 2.75	
Unrestrained	1	−100	−261	0	261	1.67	0.13	1.31 – 2.03	1.65 ± 0.06
		−50	−109	0	119	1.40	0.26	0.68 – 2.11	
		+50	181	0	191	2.32	0.20	1.76 – 2.88	
		+100	301	0	301	1.93	0.15	1.50 – 2.36	
	2	−100	−311	0	315	1.99	0.06	1.83 – 2.16	
		−50	−99	0	109	1.27	0.23	0.62 – 1.92	
		+50	122	0	136	1.56	0.19	1.03 – 2.10	
		+100	315	0	319	2.02	0.12	1.68 – 2.36	
	3	−100	−242	0	242	1.55	0.12	1.22 – 1.89	
		−50	−118	0	122	1.51	0.14	1.12 – 1.91	
		+50	134	0	146	1.72	0.12	1.40 – 2.04	
		+100	209	0	211	1.34	0.13	0.98 – 1.70	

COM restraint	Replica	Potential (mV)	Net permeation events		Gross ion transfer	Mean block conductance (nS)	Block SEM (nS)	95% CI (nS)	I-V conductance (nS)
			Cl^-	K^+					
	4	−100	−307	0	309	1.97	0.14	1.57 – 2.36	
		−50	−120	0	146	1.54	0.24	0.87 – 2.20	
		+50	147	0	155	1.88	0.19	1.35 – 2.42	
		+100	230	0	230	1.47	0.23	0.84 – 2.11	
	5	−100	−264	0	264	1.69	0.17	1.23 – 2.15	
		−50	−115	−1	128	1.46	0.21	0.88 – 2.04	
		+50	115	0	123	1.47	0.20	0.92 – 2.03	
		+100	244	0	244	1.56	0.20	1.02 – 2.11	
	6	−100	−208	0	208	1.33	0.18	0.85 – 1.82	
		−50	−131	1	144	1.69	0.19	1.16 – 2.22	
		+50	84	−2	94	1.10	0.30	0.27 – 1.94	
		+100	224	0	224	1.44	0.14	1.05 – 1.82	
**P. Empty POPC:cholesterol (2:1) membrane patch, NBFIX, 150 × 150 Å, 293 K**									
-	1	−100	0	0	0	0.00	0.00	0.00 – 0.00	0.00 ± 0.00
		−50	0	0	0	0.00	0.00	0.00 – 0.00	
		+50	0	0	0	0.00	0.00	0.00 – 0.00	
		+100	0	0	0	0.00	0.00	0.00 – 0.00	
	2	−100	0	0	0	0.00	0.00	0.00 – 0.00	

COM restraint	Replica	Potential (mV)	Net permeation events		Gross ion transfer	Mean block conductance (nS)	Block SEM (nS)	95% CI (nS)	I-V conductance (nS)
			Cl⁻	K⁺					
		−50	0	0	0	0.00	0.00	0.00 – 0.00	
		+50	0	0	0	0.00	0.00	0.00 – 0.00	
		+100	0	0	0	0.00	0.00	0.00 – 0.00	
	3	−100	0	0	0	0.00	0.00	0.00 – 0.00	
		−50	0	0	0	0.00	0.00	0.00 – 0.00	
		+50	0	0	0	0.00	0.00	0.00 – 0.00	
		+100	0	0	0	0.00	0.00	0.00 – 0.00	
	4	−100	0	0	0	0.00	0.00	0.00 – 0.00	
		−50	0	0	0	0.00	0.00	0.00 – 0.00	
		+50	0	0	0	0.00	0.00	0.00 – 0.00	
		+100	0	0	0	0.00	0.00	0.00 – 0.00	
	5	−100	0	0	0	0.00	0.00	0.00 – 0.00	
		−50	0	0	0	0.00	0.00	0.00 – 0.00	
		+50	0	0	0	0.00	0.00	0.00 – 0.00	
		+100	0	0	0	0.00	0.00	0.00 – 0.00	
	6	−100	0	0	0	0.00	0.00	0.00 – 0.00	
		−50	0	0	0	0.00	0.00	0.00 – 0.00	
		+50	0	0	0	0.00	0.00	0.00 – 0.00	

COM restraint	Replica	Potential (mV)	Net permeation events		Gross ion transfer	Mean block conductance (nS)	Block SEM (nS)	95% CI (nS)	I-V conductance (nS)
			Cl^-	K^+					
		+100	0	0	0	0.00	0.00	0.00 – 0.00	

Net permeation events are signed net crossings of each ion species along the membrane normal; gross ion transfer is the total number of crossing events in either direction. Apparent conductance (G = I/V) was calculated for each 250 ns trajectory by dividing it into five equal blocks; the mean, standard error of the mean (SEM) and two-sided 95% confidence interval (Student's t, 4 degrees of freedom) across blocks are reported. A negative permeation events indicates transport opposing the applied field. I-V conductance is the slope ± standard error of a linear least-squares fit of current against transmembrane potential across all replicas and potentials for each system and restraint condition. †Systems simulated at a single potential (+100 mV); value is the mean ± standard deviation of block conductance across the six replicas.

S3.2 Occluding Lipid Atoms

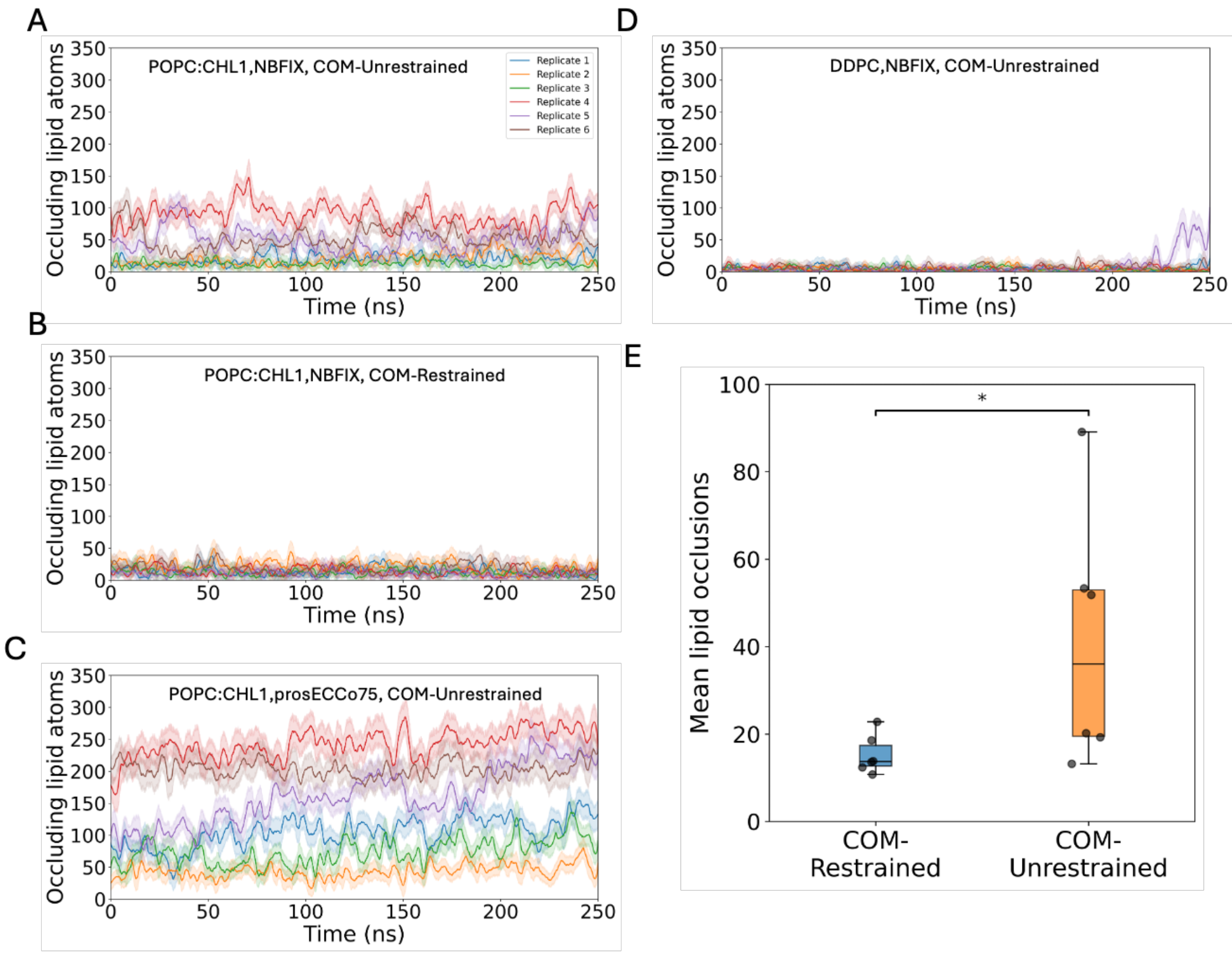

Figure S5. SoD channel occluding lipid atoms.

Calculated across varying SoD conditions for NBFIX simulations of SoD within POPC:CHL1 bilayers with a +100 mV transmembrane potential without **(A)** and with **(B)** SoD COM-restraints, and for COM-unrestrained SoD with prosECCo75 (all charges) **(C)** and with NBFIX in DDPC lipid bilayers included to illustrate minimal occlusions in the thinnest tested membrane **(D)**. **(E)** Box plot of mean lipid occlusions plotted for COM-restrained and COM-unrestrained conditions highlighting significant difference between means (permutation, $p = 0.0455$).

S3.3 SoD Tilt

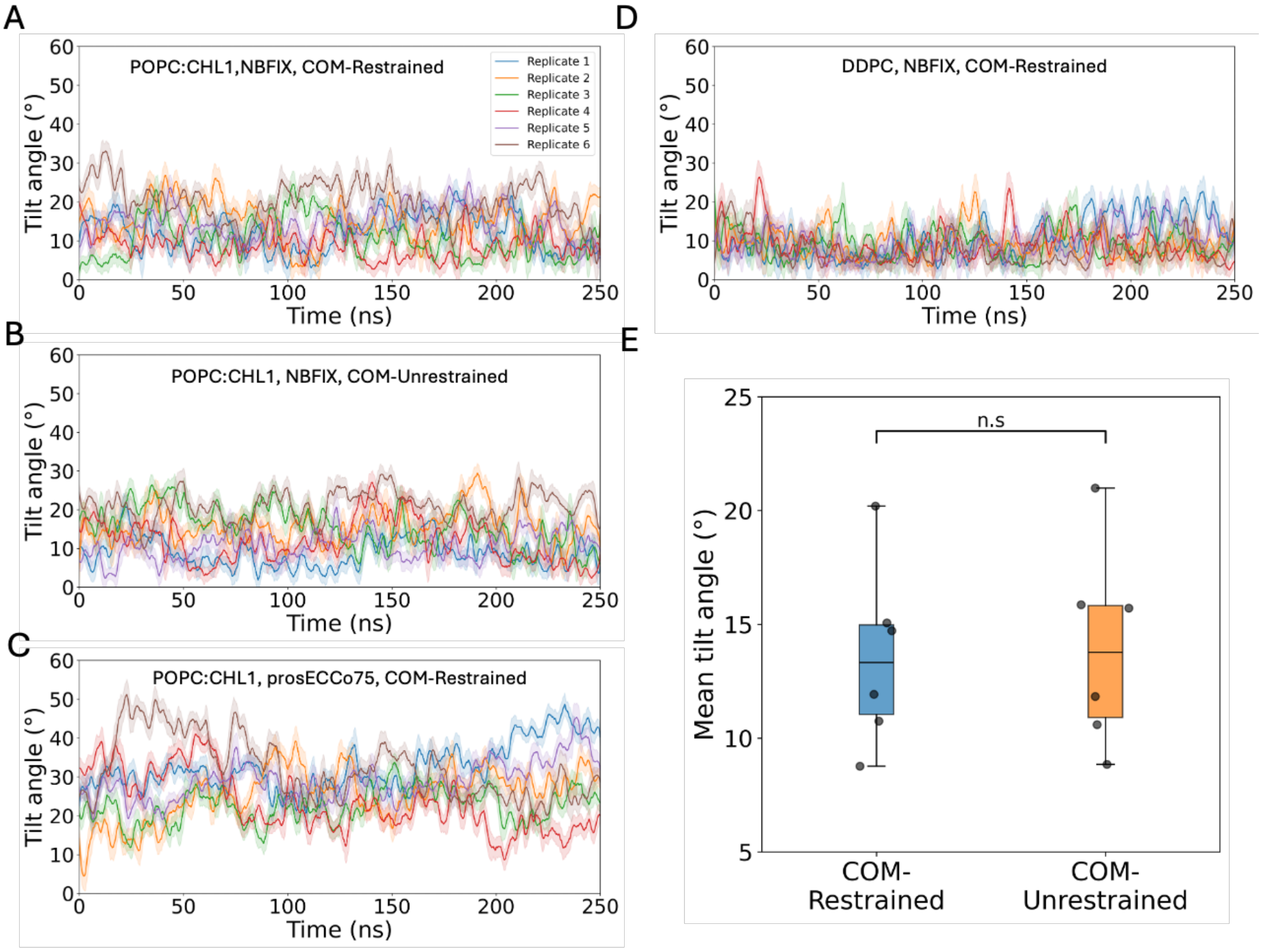

Figure S6. SoD tilt angle traces.

Calculated across varying SoD conditions for NBFIX simulations of SoD within POPC:CHL1 bilayers with a +100 mV transmembrane potential with **(A)** and without **(B)** SoD COM-restraints, and for COM-restrained SoD with prosECCo75 (all charges) **(C)** and with NBFIX in DDPC lipid bilayers to demonstrate observed tilt in the thinnest tested membrane **(D)**. **(E)** Box plot of mean SoD tilt angles plotted for COM-restrained and COM-unrestrained conditions highlights no significant difference between means (permutation, $p = 0.865$).

S3.4 SoD Embedding Depth

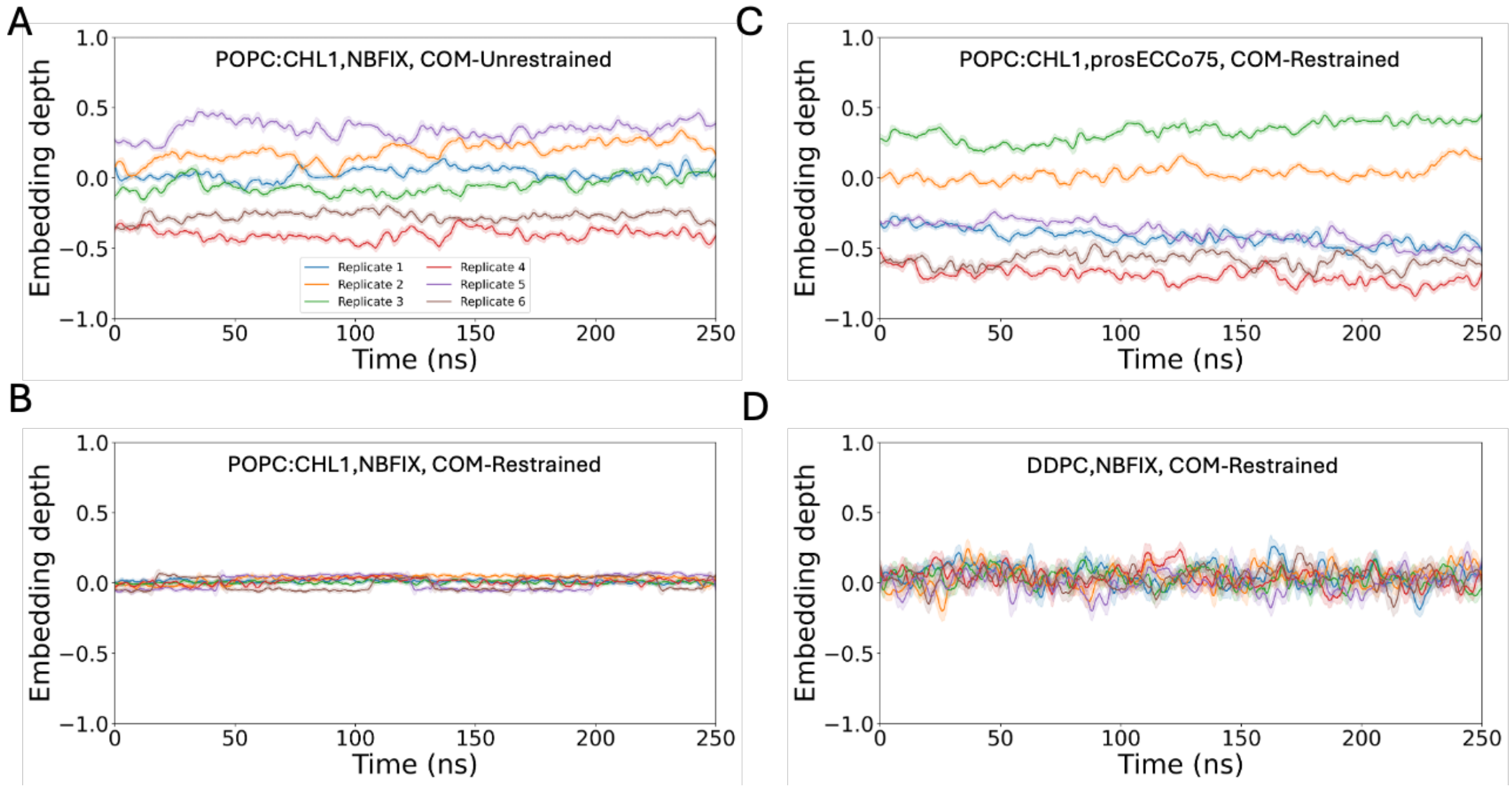

Figure S7. SoD embedding depths.

Calculated across varying SoD conditions for NBFIX simulations of SoD within POPC:CHL1 bilayers with a +100 mV transmembrane potential without **(A)** and with **(B)** SoD COM-restraints, and for COM-restrained SoD with prosECCo75 **(C)** and with NBFIX in DDPC lipid bilayers to demonstrate observed embedding depth in the thinnest tested membrane **(D)**.

S4 Explanation and sensitivity tests of analysis tools

S4.1 Radial exclusion radius for membrane boundaries

Lipids adjacent to SoD bend into the toroidal pore, so phosphate positions near the channel do not represent the bulk membrane. For each replica, a lateral exclusion radius r_{excl} beyond which the bilayer has bulk thickness was determined from the radial membrane-thickness profile.

In each analysed frame, phospholipid phosphorus (P) atoms were assigned to the upper or lower leaflet according to whether they lay above or below the centre of mass of all P atoms. Their lateral distances r from the SoD centre of mass (macrocycles) were calculated in the xy plane using the minimum-image convention. P atoms were binned by r (1 Å bins from 10 Å to $L_x/2$), and the local thickness in each bin was the difference between the mean z of upper- and lower-leaflet P atoms. Profiles were averaged over frames, with the standard error of the mean in each bin. The mean profile was fitted with an exponential approach to a plateau,

$$T(r) = T_{bulk} - \Delta T \cdot \exp[-(r - r_0)/\xi],$$

by weighted non-linear least squares (scipy[19] curve_fit, weights from the per-bin standard errors, floored at 0.2× their median to avoid over-weighting sparsely populated bins), where T_{bulk} is the bulk thickness, ΔT the thinning near SoD, r_0 the inner fit limit and ξ the decay length. r_{excl} was defined as the radius at which the fitted thickness reaches within 2% of T_{bulk}:

$$r_{excl} = r_0 - \xi \cdot \ln(0.02 \cdot T_{bulk} / \Delta T).$$

In subsequent analyses (Sections S4.1 and S4.3), P atoms within r_{excl} of the SoD axis were excluded when defining the membrane boundaries. No exclusion was applied in SoD-free control membranes.

S4.2 Ion Permeation and Single-Channel Conductance Calculations

Ion permeation was analysed in the 250 ns NVT trajectories run under applied fields ($\Delta V = E \cdot L_z$; Methods), using a custom MDAnalysis[6] script. Trajectories were first made whole with gmx trjconv (-pbc mol) and centered on the bilayer. SoD-containing systems were centred on SoD before the phospholipids.

Membrane boundaries

In each frame (50 ps intervals), phospholipid phosphorus (P) atoms were assigned to the upper or lower leaflet according to whether they lay above or below the centre of geometry of all phospholipid P atoms. P atoms within a cylinder of radius r_{excl} around the SoD axis (centre of geometry of the macrocycles; spanning the full box height) were excluded. Here, r_{excl} is the replica-specific radius at which the bilayer reaches bulk thickness (**Section S4.5**). No exclusion was applied in SoD-free controls. The upper and lower membrane boundaries (z_U and z_L, respectively), were the mean z positions of the remaining upper- and lower-leaflet P atoms in each leaflet.

Ion states and events

In each frame, every K^+ and Cl^- ion was assigned to the upper compartment (+1; $z > z_U$), the lower compartment (−1; $z < z_L$) or the membrane (0). A permeation event was a consecutive +1 → 0 → −1 (inward) or −1 → 0 → +1 (outward) sequence of states. Excursions returning to the same compartment (+1 → 0 → +1, −1 → 0 → −1), and crossings incomplete at either end of the trajectory, were not counted. No dwell-time criterion was applied. An ion was flagged as wrapping across the periodic boundary whenever its z coordinate changed by more than $L_z/2$ between frames, and any candidate event with a wrap was discarded.

Conductance

For each species, the net crossing number was $N_i = N_{inward} - N_{outward}$. The transported charge was $Q = |\Sigma\ N_i\ q_i|$, with $q_{K^+} = +e$ and $q_{Cl^-} = -e$. A positive field drives cations towards +z and anions towards −z, so both species carry charge in the same direction. For each replica and

potential, the current was $I = Q/t$ (t = 250 ns) and the apparent conductance $G = I/|\Delta V|$. Within-trajectory uncertainty was estimated in two ways:

Block analysis

> Events were binned by exit frame into five 50 ns blocks, and the signed block conductances were summarised as mean ± SEM, with 95% CIs from Student's t (4 d.o.f.).

Shot noise

> $\sigma_G \approx G/\sqrt{N}$, where N is the total number of events in either direction.

The conductance of each system was taken as the slope ± SE of a linear least-squares fit of current against ΔV, constrained through the origin, across all replicas and potentials (**Table S14**).

S4.3 SoD Tilt Angle Analysis

SoD orientation was described by the normal to the best-fit plane through its six Fe(II) ions, which are distributed symmetrically around the catenane and approximately coplanar, and so serve as a proxy for the mean molecular plane. In each frame (50 ps intervals), with molecules whole, the Fe(II) positions r_i ($i = 1…6$) were centred on their centroid:

$$c = (1/6)\ \Sigma_i\ r_i,\quad X = [r_1 - c, …, r_6 - c]^T$$

The singular value decomposition $X = U\ \Sigma\ V^T$ was computed, and the plane normal $\hat{n}$ was taken as the right singular vector with the smallest singular value (the last row of V^T), i.e. the direction of least spread of the Fe(II) positions. Because $\hat{n}$ and $-\hat{n}$ describe the same plane, $\hat{n}$ was inverted when $n_z < 0$, so that it always points towards +z. The tilt angle is

$$\theta = \arccos(\hat{n} \cdot \hat{z}) = \arccos(n_z),\ \theta \in [0°, 90°],$$

where $\hat{z}$ is the unit vector along the membrane normal (the box z-axis). The argument was clipped to [−1, 1] to guard against floating-point rounding. $\theta = 0°$ corresponds to the catenane plane lying parallel to the bilayer (the horizontal orientation) and $\theta = 90°$ to the catenane plane parallel to the membrane normal (vertical). Tilt was calculated for each replica, and the means and standard deviations reported are over frames of each replica ($n = 6$).

S4.4 SoD Embedding Depth Analysis

In each frame (50 ps intervals), the upper and lower membrane boundaries z_U and z_L were the mean z positions of upper- and lower-leaflet phospholipid P atoms, assigned relative to the centre of geometry of all P atoms and excluding P atoms within the lateral radius r_{excl} of the SoD axis (as in Section S4.1). The bilayer midplane and half-thickness were

$$z_{mid} = (z_U + z_L)/2, \; h = (z_U - z_L)/2,$$

and the normalised embedding depth was

$$d = (z_{SoD} - z_{mid})/h,$$

where z_{SoD} is the z coordinate of the centre of geometry of the SoD macrocycles. $d = 0$ when the SoD centre lies at the bilayer midplane, and $d = +1$ or -1 when it lies at the mean phosphate plane of the upper or lower leaflet; $|d| > 1$ indicates a centre beyond the phosphate plane. Because occlusion and conductance depended on displacement from the midplane in either direction, analyses used the absolute depth $|d|$ (0, midplane; 1, phosphate plane).

S4.5 Channel Lipid Occlusion Analysis

S.4.5.1 Analysis overview

Lipid penetration into the SoD channel was quantified as the number of lipid atoms within a cylindrical volume co-axial with SoD in each trajectory frame. Lipid atoms inside the channel, whether acyl-chain or head-group atoms, would be expected to impede ion permeation.

In each frame, lipid atoms were selected using the MDAnalysis cylindrical zone selection (cyzone) within a cylinder of radius 5 Å, centred on the centre of geometry of the SoD macrocycles, and aligned with the membrane normal (z). The radius was chosen to exceed the inner radius of the SoD channel (~3 Å, from HOLE profiles; Results). The cylinder limits were set relative to the SoD centre so that the cylinder spanned the full box height (z = 0 to L_z), ensuring all penetrating atoms were counted regardless of the vertical position of SoD within the membrane. All atoms of all lipid residues (POPC and cholesterol, or DDPC, DCPC, DLPC, DMPC), including hydrogens, were counted. Trajectories were made whole and centred on the membrane before analysis.

Occlusion was calculated for every saved frame (50 ps intervals) of the conductance simulations (250 ns per replica). For each replica, the mean and standard deviation over frames were calculated; values reported in the main text and figures are the mean ± sample standard deviation of replica means (n = 6).

S4.5.1 Sensitivity to cylinder radius

The sensitivity of the occlusion analysis to cylinder radius was assessed over radii of 2-10 Å (0.5 Å steps) for the six COM-unrestrained POPC:cholesterol (NBFIX) replicas under a +100 mV transmembrane potential, in which embedding depth and occlusion vary (**Fig. 1, G. H; S5, S7**). Embedding depth was used as its absolute value (|d|; 0 at the bilayer midplane, 1 at the mean phosphate plane), because occlusion increased with displacement from the midplane in either direction.

Between replicas

Relationships between replica means of |d|, occlusion and conductance (n = 6) were assessed by Pearson correlation with exact two-sided permutation tests (all 720 orderings), which make no assumption of normality. The relationship between |d| and occlusion was significant at every radius tested ($r = 0.86$-0.92, $p \leq 0.039$), being strongest at 3.5-5 Å ($r = 0.92$) and weakening slightly at larger radii (**Fig. S8D**). The inverse relationship between occlusion and

conductance was also significant at every radius ($r = -0.91$ to -0.98, $p \leq 0.014$) and strengthened with radius. |d| and conductance were not significantly related ($r = -0.81$, $p = 0.068$). At 5 Å, |d| correlated with occlusion ($r = 0.92$, $p = 0.008$) and occlusion with conductance ($r = -0.95$, $p = 0.008$).

Within replicas

Correlations between |d| and occlusion were calculated in two ways. First, per-frame Pearson correlations were computed for each replica, with significance based on an effective sample size corrected for the autocorrelation of both series, and combined across replicas by random-effects meta-analysis on Fisher z. Second, 50 ns block means were centred on each replica's own mean and pooled across replicas (Spearman ρ, used here to limit the influence of individual blocks from highly variable replicas; 95% CI from 10,000 cluster-bootstrap resamples of whole replicas, random seed 1).

The association between |d| and occlusion was positive at all radii. It was significant for radii of 2-6.5 Å by both methods (block $\rho = 0.53$–0.61; per-frame $r = 0.11$–0.20; **Fig. S8B, C**) and weakened at larger radii, where the cylinder increasingly samples lipid surrounding the channel. Integrated autocorrelation times were estimated as $\tau_{int} = 1 + 2\Sigma_k\, \rho(k)$, with the sum truncated where the autocorrelation function first crosses zero; block lengths were also checked by the plateau of block-averaged SEM with block length[20]. Per-frame correlations were weaker than block correlations because occlusion fluctuates on faster timescales (τ = 1-25 ns) than embedding depth (τ = 11–45 ns), and averaging over 50 ns removes this fast noise. For two replicas, the 50 ns blocks were shorter than twice the integrated autocorrelation time of embedding depth, so adjacent block means are not fully independent. The cluster bootstrap, which resamples whole replicas, does not assume they are. The block correlation was robust to leaving out any single replica ($\rho = 0.42$–0.69 at 5 Å).

Block-to-block changes in occlusion predicted block conductance for radii of 5-10 Å ($\rho = -0.37$, 95% CI −0.63 to −0.05 at 5 Å; $\rho = -0.37$ to -0.55 across 5-10 Å), but not at 2–4.5 Å ($\rho = -0.17$ to -0.33; CIs spanning zero). Block-to-block changes in |d| did not predict conductance ($\rho = 0.02$, 95% CI −0.34 to 0.29). Both between and within replicas, conductance therefore tracks lipid occlusion rather than embedding depth directly, consistent with depth influencing conduction through its effect on occlusion. These relationships are correlational, and 50 ns block conductances are subject to Poisson noise.

The 5 Å radius used in the main text therefore lies within the range over which occlusion is significantly related both to embedding depth (all radii between replicas; 2-6.5 Å within replicas) and to conductance (all radii between replicas; 5-10 Å within replicas).

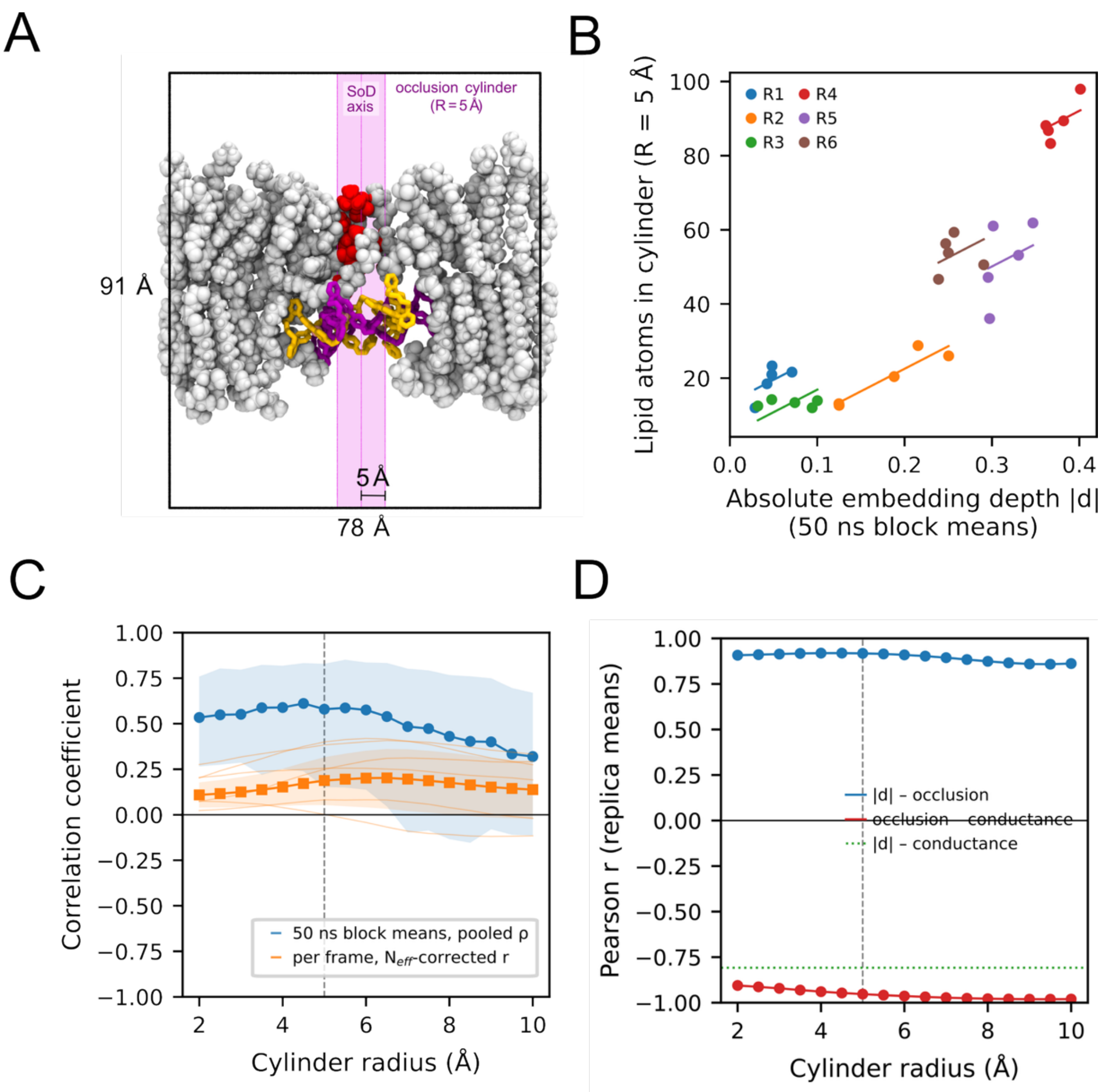

Figure S8. Lipid occlusion metric and sensitivity to cylinder radius.

(A) Frame from a COM-unrestrained replica (R4, 150 ns, +100 mV). Lipid atoms within the R = 5 Å cylinder around the SoD axis, spanning the full box height, are shown in red; other lipids in grey; SoD in yellow and purple. **(B)** 50 ns block means of absolute embedding depth |d| and occlusion at R = 5 Å, coloured by replica; lines show the common within-replica slope through each replica's mean; ρ is the pooled within-replica Spearman correlation (95% CI). **(C)** Within-replica |d|-occlusion correlation against cylinder radius: 50 ns block means (blue) and autocorrelation-corrected per-frame values combined across replicas (orange), with 95% CIs; thin lines show individual replicas (per frame). **(D)** Between-replica Pearson correlations (replica means, n = 6) of |d| with occlusion and of occlusion with conductance against cylinder radius; filled markers, exact permutation $p < 0.05$; dotted line, |d|-conductance. Dashed vertical lines mark R = 5 Å. Six COM-unrestrained POPC:cholesterol (NBFIX) replicas at +100 mV; see Section S4.4 for details.

S4.6 Bulk Electrolyte Conductivity

Bulk conductivity was calculated from 250 NVT trajectories of the six KCl boxes (**Section S5.2**), run under fields giving nominal potentials of ±50 and ±100 mV. A custom MDAnalysis script was used, and all frames were analysed (50 ps intervals).

Twenty planes normal to z were placed at $z_k = (k - ½)L_z/20$, so that none coincided with a box edge. Between consecutive frames, each ion was counted as crossing every plane between its previous and current z position (wrapped into $[0, L_z)$). Each crossing contributed +q for motion along +z and −q for motion along −z, with q = +e for K^+ and −e for Cl^-, as in the transmembrane analysis (**Section S4.2**). Any frame-to-frame displacement larger than $L_z/2$ was treated as a periodic wrap and not counted. The transported charge Q was averaged over the 20 planes, and the current was I = Q/T, where T is the analysed trajectory length.

For each replica, the current density $j = I/(L_xL_y)$ was fitted against the applied field E at the four potentials by least squares through the origin, giving $\kappa = \Sigma E_m j_m / \Sigma E_m^2$. E was read directly from each simulation’s .mdp file, so the fit does not depend on the box length used to set the field. Fit quality is reported as R^2 and the standard error of the slope (**Table S17**), and κ as the mean ± SD across replicas.

S4.7 HOLE

Pore radius profiles were calculated with HOLE[21] via the MDAnalysis HoleAnalysis interface. Because HOLE assigns van der Waals radii by atom name, atom names in the analysed structures were first reduced to their element symbols (C, H, N, O, P, Fe), and radii were taken from the HOLE simple.rad set, with an added Fe radius of 1.10 Å (from SURFNET[22]). SoD (macrocycles and Fe(II) ions) and all lipid atoms were included as pore-lining atoms; water and ions were excluded. The channel vector was the membrane normal (cvect = 0 0 1), with HOLE's default initial point, sampling distance (0.2 Å) and end radius (22 Å). No structural alignment was applied: trajectories were made whole and centred on the membrane. For equilibrium simulations, profiles were computed over the final 200 ns of each replica (2 ns intervals); for simulations under applied potentials, 100 frames were evenly spaced over the full 250 ns (2.5 ns intervals). Radii were binned along the channel coordinate (100 bins) and averaged over frames. The minimum radius of each frame was averaged per replica and reported as the mean ± SD of replica means (n = 6).

S4.8 Leaflet curvature

For each leaflet, phosphorus z positions (relative to the membrane centre of geometry) were binned on a 2 Å x-y grid centred on SoD and averaged over the analysis window to give a height surface, $h(x, y)$. Bins with fewer than 20% of the median bin occupancy were excluded, and h was smoothed with a NaN-aware Gaussian filter ($\sigma = 3$ Å). Mean (H) and Gaussian (K) curvatures were calculated from the full Monge-gauge expressions, with the sign of H defined so that $H > 0$ indicates a leaflet bulging towards its own solvent phase in both leaflets. Bulk curvature was summarised as the mean and RMS of H, and the mean of K, over bins outside the exclusion radius (all bins for empty membranes), excluding the outer $2\sigma + 1$ bin of the grid where derivatives are one-sided. Near-pore features were taken as the minimum (trough) and maximum (ridge) of the radially averaged H profile (2 Å annuli, $r \geq 4$ Å). Values are reported per leaflet and as the mean of both leaflets, as mean ± SD across replicates.

S4.9 Membrane Thickness

Membrane thickness was calculated with MDAnalysis[6] as the distance along z between the mean phosphorus positions of the upper and lower leaflets. In each frame, phospholipid phosphorus atoms were assigned to a leaflet according to whether they lay above or below the membrane's centre of geometry along z. To exclude lipids perturbed by SoD, phosphorus atoms within a cylinder centred on the SoD centre of geometry and spanning the full box height were removed. The cylinder radius was the plateau radius fitted for each replicate (see Section S4.1). No exclusion was applied to empty-membrane controls. For each replicate, thickness was averaged over frames from 250 ns onwards, and values are reported as mean ± SD across the six replicates.

S4.10 Lipid Headgroup Orientation

Lipid headgroup orientation was calculated with MDAnalysis[6] as the angle (θ), between each lipid's P→N vector and the outward normal of its leaflet (+z for the upper leaflet, −z for the lower), so that 0° corresponds to the choline pointing into the solvent and 90° to a headgroup lying in the membrane plane. Leaflets were assigned as in the thickness analysis. For two-dimensional maps, each lipid was binned by the x-y position of its phosphorus atom relative to the SoD centre of geometry (or the membrane centre of geometry for empty-membrane controls) on a 2 Å grid, and θ was averaged per bin and per leaflet over frames from 50 ns onwards and across the six replicates.

S5 Calibration of Simulated Conductances Against Bulk Electrolyte Conductivity

To allow comparison with experiment, simulated conductances were calibrated against the bulk conductivity of the simulated electrolyte. We calculated the conductivity of 1 M KCl boxes under each force-field model and temperature and compared it with experimental reference values for KCl at the same molality and temperature.

S5.1 Simulated electrolytic conductivity

Six bulk electrolyte boxes were prepared with CHARMM-GUI Solution Builder (target 1 M KCl; **Table S9C**). Each contained 369 K^+ and 369 Cl^- ions in 17,444-17,504 TIP3P water molecules. They were minimised, equilibrated and run under applied fields using the protocol in **Table S11**, without position restraints and with isotropic pressure coupling during equilibration. Conductivity (κ_{sim}) was calculated at ±50 and ±100 mV as described in **Section S4.6**, for NBFIX at 293 K and 310 K and for prosECCo75 (all charges) at 293 K. Current was linear in the applied field for every replica ($R^2 > 0.9999$).

S5.2 Experimental reference

Reference values were obtained from the smoothing polynomials of Wu et al.[23] for KCl at 1.0 mol kg^{-1} and at 1 D. The 1 D composition is 71.1352 g KCl per 1000 g of solution, i.e. 1.02725 mol kg^{-1}. Both polynomials were evaluated at the simulation temperature and extrapolated linearly in molality to the simulated value:

$$\kappa_{exp} = \kappa_{1m} + (b - 1)(\kappa_{1D} - \kappa_{1m})/(b_{1D} - 1)$$

Because KCl conductivity increases sub-linearly with molality, this extrapolation may slightly overestimate κ_{exp}, and therefore slightly overestimate the calibrated conductances.

S5.3 Calibration

Each simulated conductance (**Table S15**) was divided by $f = \kappa_{sim} / \kappa_{exp}$ (**Table S16**), with uncertainties combined in quadrature. Under NBFIX at 293 K, bulk conductivity is overestimated by 33%, consistent with the systematic overestimation of KCl bulk conductivity reported for CHARMM36 with TIP3P in previous applied-field simulations[24]. The Fe(II)-only prosECCo75 model retains the NBFIX K^+-Cl^- parameters, so it uses the NBFIX correction factor. Calibrated conductances are given in **Table S17**.

Table S15. Bulk electrolyte conductivities for NBFIX and prosECCo75 models.

System						
Charge-scaling	Temperature (K)	Replica	κ (S m⁻¹)	SE of fit (S m⁻¹)	R^2	Mean κ (S m⁻¹)
NBFIX	293	1	15.1238	0.0308878	0.999987	15.1726 ± 0.0742
		2	15.1902	0.0509806	0.999966	
		3	15.1882	0.0123582	0.999998	
		4	15.1996	0.0599192	0.999953	
		5	15.2759	0.0586705	0.999956	
		6	15.0578	0.0339757	0.999985	
	310	1	17.2165	0.0284127	0.999992	17.2568 ± 0.0508
		2	17.3270	0.0574623	0.999967	
		3	17.1873	0.0442110	0.999980	
		4	17.2443	0.0266972	0.999993	
		5	17.2843	0.0432512	0.999981	
		6	17.2814	0.0396015	0.999984	
prosECCo75	293	1	18.9073	0.0427112	0.999985	18.8855 ± 0.0407
		2	18.9010	0.0829893	0.999942	
		3	18.8706	0.0242194	0.999995	
		4	18.9467	0.0500810	0.999979	
		5	18.8509	0.0691540	0.999960	
		6	18.8365	0.0764036	0.999951	

Table S16. Charge-scaling simulated-experimental conductivity correction factors.

Charge-scaling method	Temperature (K)	κ_{sim} (S m^{-1})	κ_{exp} (S m^{-1})	Correction factor
NBFIX	293	15.17	11.43	1.326867146
	310	17.26	15.15	1.138989129
prosECCo75	293	18.89	11.43	1.651565947

Table S17. Raw and calibrated simulated conductances.

COM-Restraint	Calibration coefficient	Raw conductance (nS)	Calibrated conductance (nS)
A. Monomeric, Horizontal, POPC:cholesterol (2:1), NBFIX, 85 x 85 Å, 293 K			
Restrained	1.3269	2.05 ± 0.03	1.54 ± 0.02
Unrestrained		1.34 ± 0.14	1.03 ± 0.10
B. Monomeric, Horizontal, POPC:cholesterol (2:1), prosECCo75 (all charges scaled), 85 x 85 Å, 293 K†			
Restrained	1.6516	0.69 ± 0.18	0.42 ± 0.11
Unrestrained		0.26 ± 0.34	0.16 ± 0.21
C. Monomeric, Horizontal, POPC:cholesterol (2:1), prosECCo75 (FE2P only), 85 x 85 Å, 293 K†			
Restrained	1.3269	0.51 ± 0.11	0.38 ± 0.0908
Unrestrained		0.21 ± 0.18	0.19 ± 0.1302
D. Monomeric, horizontal, POPC:cholesterol (2:1), NBFIX, dihedral restraints, 85 × 85 Å, 293 K			
Restrained	1.3269	0.68 ± 0.05	0.52 ± 0.04
Unrestrained		0.45 ± 0.05	0.34 ± 0.04
E. Monomeric, Horizontal, DDPC, NBFIX, 85 x 85 Å, 310 K			
Restrained	1.1390	3.86 ± 0.04	3.39 ± 0.04
Unrestrained		3.96 ± 0.05	3.47 ± 0.04
F. Monomeric, Horizontal, DCPC, NBFIX, 85 x 85 Å, 310 K			
Restrained	1.1390	3.79 ± 0.05	3.32 ± 0.04
Unrestrained		3.78 ± 0.06	3.32 ± 0.05
G. Monomeric, Horizontal, DLPC, NBFIX, 85 x 85 Å, 310 K			
Restrained	1.1390	3.46 ± 0.08	3.03 ± 0.07
Unrestrained		3.42 ± 0.04	3.00 ± 0.04
H. Monomeric, Horizontal, DMPC, NBFIX, 85 x 85 Å, 310 K			
Restrained	1.1390	2.95 ± 0.03	2.59 ± 0.03
Unrestrained		3.06 ± 0.06	2.69 ± 0.05
I. Dimeric, Horizontal, POPC:cholesterol (2:1), NBFIX, 85 x 85 Å, 293 K			
Unrestrained	1.3269	1.84 ± 0.02	1.38 ± 0.02
J. Monomeric, Horizontal, POPC:cholesterol (2:1), NBFIX, 150 x 150 Å, 293 K			
Restrained	1.3269	2.06 ± 0.05	1.56 ± 0.03
Unrestrained		1.65 ± 0.06	1.24± 0.04

Raw conductances are I-V slopes ± SE (Table S14), except †, which was simulated at +100 mV only and is reported as the apparent conductance mean ± SD across replicas.

S6 ProsECCo75

To elucidate the role of ionic interactions within the stabilization of lipid-toroidal pores for simulations of pre-embedded SoD monomers, charge-scaling by a factor of 0.75 (prosECCo75[25]) was applied to charged groups within the system, with the following two protocols: (1) Charge-scaling applied to all charged species within the system - POPC, Fe(II), Cl^-, K^+; (2) charge-scaling applied only to the six Fe(II) ions of SoD to directly probe the role of Fe(II)-interactions.

S6.1 Simulation details

In the prosECCo75 model, the net charges of the phospholipid choline and phosphate groups were scaled from +1 and −1 e to +0.75 and −0.75 e by adjusting the partial charges of the choline hydrogens (+0.25 → +0.215 or +0.23 e) and the non-ester phosphate oxygens O13 and O14 (−0.78 → −0.655 e); all other POPC parameters, including atom types and Lennard-Jones parameters, were identical to CHARMM36. K^+ and Cl^- charges were scaled to ±0.75 e and Fe(II) to +1.5 e; the partial charges of the net-neutral SoD macrocycles were not scaled. Electroneutrality was maintained by default in prosECCo75 protocol (1), whilst electroneutrality was maintained in (2) through deletion of 3 Cl^- ions per system (6 x Fe(II) x 0.5 e). All NBFIX terms (**Table S4**) were disabled in (1) whilst NBFIX terms were active in (2).

Table S18. prosECCo75 charge-scaled atoms.

Species	Atoms	Original charge	New charge
K+	POT	+1.000	+0.750
Cl-	CLA	-1.000	-0.750
Fe(II)	FE2P	+2.000	+1.500
POPC	H12A	+0.250	+0.215
	H12B	+0.250	+0.215
	H13*	+0.250	+0.230
	H14*	+0.250	+0.230
	H15*	+0.250	+0.230
	O13	-0.780	-0.655
	O14	-0.780	-0.655

S6.2 Bulk 1 M KCl conductivity

To test whether charge scaling alters intrinsic ion mobility, independently of the pore, we computed the conductivity of 1 M KCl at 293 K under each model using the same settings and applied field as the pore simulations (**Table S19**). Both models overestimate the experimental conductivity (11.43 S m^{-1}), by 33% for NBFIX (15.17 S m^{-1}) and 65% for prosECCo75 (18.89 S m^{-1}). Because ions are more mobile in bulk under prosECCo75, reduced ion mobility cannot explain the lower pore conductances which instead point to changes in the pore itself.

Table S19. Calculated bulk electrolyte conductivity values.

Model	Conductivity (S m^{-1})
CHARMM36/NBFIX	15.17
prosECCo75 ("all charges")	18.89
Experimental[a]	11.43

Calculated for simulated boxes under CHARMM36/NBFIX charges or prosECCo75 "all charges" models. a. Please see **Section S5.4** for details of experimental reference calculations.

S6.3 Fe(II)-phosphate interactions

To assess the effect of charge scaling on Fe(II)-lipid interactions, we analysed simulations in which the SoD COM was restrained, so that differences in SoD insertion depth between models did not bias the comparison. In our primary NBFIX simulations, POPC atoms within 6 Å of Fe(II) occurred mainly with the phosphate oxygens (O13, O14, O11) and the phosphorus atom (P), with smaller contributions from the glycerol/ester region (e.g. O32) and the acyl chains (**Fig. S9, A, B**). Scaling only the Fe(II) charge reduced the mean total number of Fe(II)-lipid contacts from 2.43 ± 0.60 contacts $Fe(II)^{-1}$ $frame^{-1}$ to 1.05 ± 0.19 contacts $Fe(II)^{-1}$ $frame^{-1}$ (**Fig. S9C**). Phosphate and glycerol/ester contacts were strongly reduced, while acyl chain contacts increased 3.2-fold, so that the acyl chains accounted for most Fe(II)-lipid contacts (**Fig. S9B**). The time-weighted mean contact lifetime also decreased (16.16 ± 8.23 ns to 2.36 ± 2.98 ns; permutation test, $p = 0.002$) (**Fig. S9E**). In this model, the first peak of the Fe(II)-phosphate oxygen (O13, O14) RDF g(r) was below 1, indicating that phosphate oxygens are more depleted near Fe(II) compared to NBFIX and prosECCo75 ("all charges") (**Fig. S9D**). When all charges were scaled, the total number of contacts was similar to NBFIX (2.45 ± 0.79 contacts $Fe(II)^{-1}$ $frame^{-1}$ vs 2.43 ± 0.60 contacts $Fe(II)^{-1}$ $frame^{-1}$), and lifetimes were not significantly different (9.83 ± 10.38 ns vs 16.16 ± 8.23 ns; permutation test, $p = 0.273$). Phosphate contacts were largely retained, but glycerol/ester contacts were reduced, and choline and acyl chain contacts increased (**Fig. S9B**). The g(r) first peak shifted outward from 4.35 Å to 4.75 Å. Scaling only the Fe(II) charge therefore largely abolishes Fe(II)-phosphate association, whereas scaling all charges preserves it.

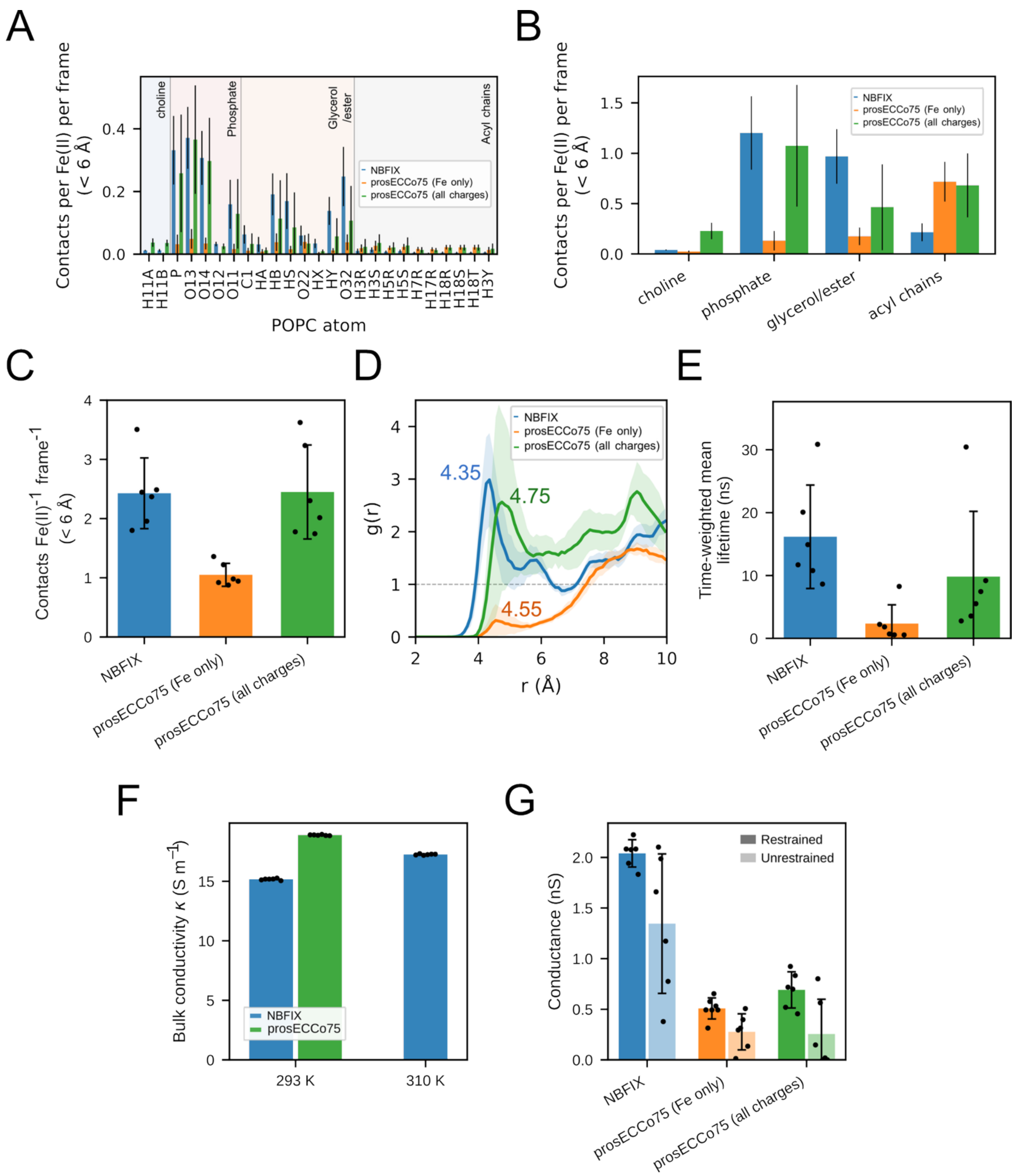

Figure S9. Fe(II)-lipid interactions, bulk conductivity and pore conductance under each charge-scaling model.

(A) Top 25 contacts per Fe(II) per frame (< 6 Å) with each POPC atom, grouped by region (choline, phosphate, glycerol and acyl chains) for all three models. **(B)** Fe(II) - POPC contacts classified by region. **(C)** Total Fe(II)-POPC contacts per Fe(II) per frame. **(D)** RDF g(r) between Fe(II) and POPC phosphate oxygens (O13 O14). First peak positions are labelled. **(E)** Time-weighted mean Fe(II)-lipid contact lifetimes. **(F)** Conductivity of bulk 1 M KCl, compared with the experimental reference (see section S5.4). The "Fe(II) only" model shares the same KCl

parameters as the NBFIX model, so it is not shown separately. **(G)** SoD-lipid channel conductances under each method.

S6.4 Influence of charge scaling model on toroidal pore stability

Both prosECCo75 protocols reduced the pore conductance (NBFIX: 2.04 ± 0.14 nS; "Fe(II) only": 0.51 ± 0.11 nS; "all charges": 0.69 ± 0.18 nS; **Fig. S9G**). To determine how the pore changed, we first computed density maps of choline and phosphate atoms in the membrane core ($|z - z_P| < 10$ Å; **Fig. S10A, B**). Under both prosECCo75 protocols the headgroup ring lay closer to the SoD than under NBFIX (**Fig. S10A, B, right**), and choline density extended further into the lumen, indicating that the pore remained toroidal and headgroup-lined but was narrowed by encroaching headgroups. HOLE profiles confirmed a partial constriction: the minimum pore radius decreased from 3.12 ± 0.09 Å (NBFIX) to 2.32 ± 0.09 Å ("Fe(II) only") and 2.25 ± 0.15 Å ("all charges") (**Fig. S10C**). Consistently, the number of lipid atoms within 5 Å of the pore axis more than doubled (NBFIX: 15.3 ± 4.5; "Fe(II) only": 34.3 ± 3.8; "all charges": 39.4 ± 8.0; $p = 0.002$ vs NBFIX for both, permutation test, $n = 6$; Fig**. S10D**). The pore therefore narrowed but did not close. The SoD also tilted more under charge scaling (NBFIX: 13.6 ± 4.0°; "Fe(II) only": 19.9 ± 2.5°; "all charges": 27.5 ± 4.3°; **Fig. S10E**). However, "all charges" produced significantly greater tilt than "Fe(II) only" ($p = 0.006$) without significantly greater occlusion, narrowing or conductance loss, indicating that tilt accompanies the constriction but is not its primary cause.

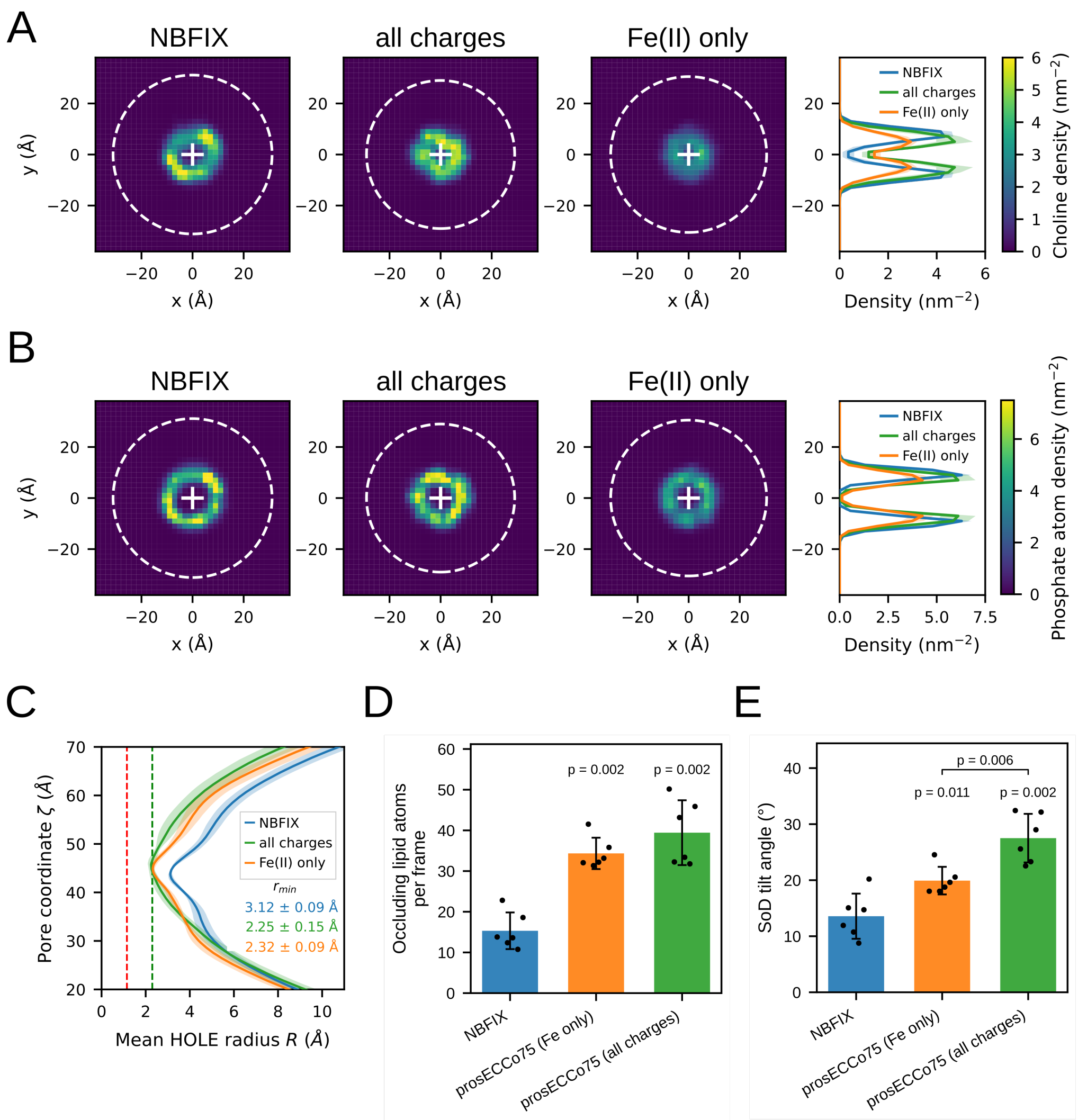

Figure S10. Pore constriction under charge scaling.

(A, B) Replicate-mean (n = 6) density maps of **(A)** choline heavy atoms (N, C11-C15) and **(B)** phosphate atoms (P, O11-O14) within the membrane core ($|z - z\_P| < 10$ Å from the phosphorus midplane), centred on the SoD (+), averaged over both leaflets. The dashed circle marks plateau radius of membrane deformation, rexcl. Right: radial profiles (mean ± SD shaded). **(C)** Mean HOLE pore-radius profiles (mean ± SD shaded, n = 6). **(D)** Lipid atoms within a 5 Å-radius cylinder centred on the SoD. (E) SoD tilt: angle between the Fe(II)-plane normal and the membrane normal (0° = parallel to the membrane). **(D, E)** Mean ± SD with replicate points; p-values from permutation t test.

Weakening the Fe(II) charge, either alone or together with lipid and ion charges, constricted the pore by headgroup encroachment and reduced its conductance, indicating that Fe(II) electrostatics help keep the toroidal pore open. Because constriction occurred to a similar extent whether Fe(II)-phosphate contacts were lost ("Fe(II) only") or retained ("all charges"; Section S6.3), it is not explained by loss of Fe(II)-phosphate association alone; the inward shift of choline density towards the SoD under both protocols suggests that reduced electrostatic exclusion of choline groups may contribute.

S7 Stability of Dimeric SoD Assemblies

To assess whether pre-assembled SoD dimers remain stable within the membrane, pre-embedded dimers were simulated in POPC:cholesterol (2:1) under applied transmembrane potentials of −100, −50, +50 and +100 mV, using the same protocols as for monomeric systems (see Methods), but without SoD COM restraints.

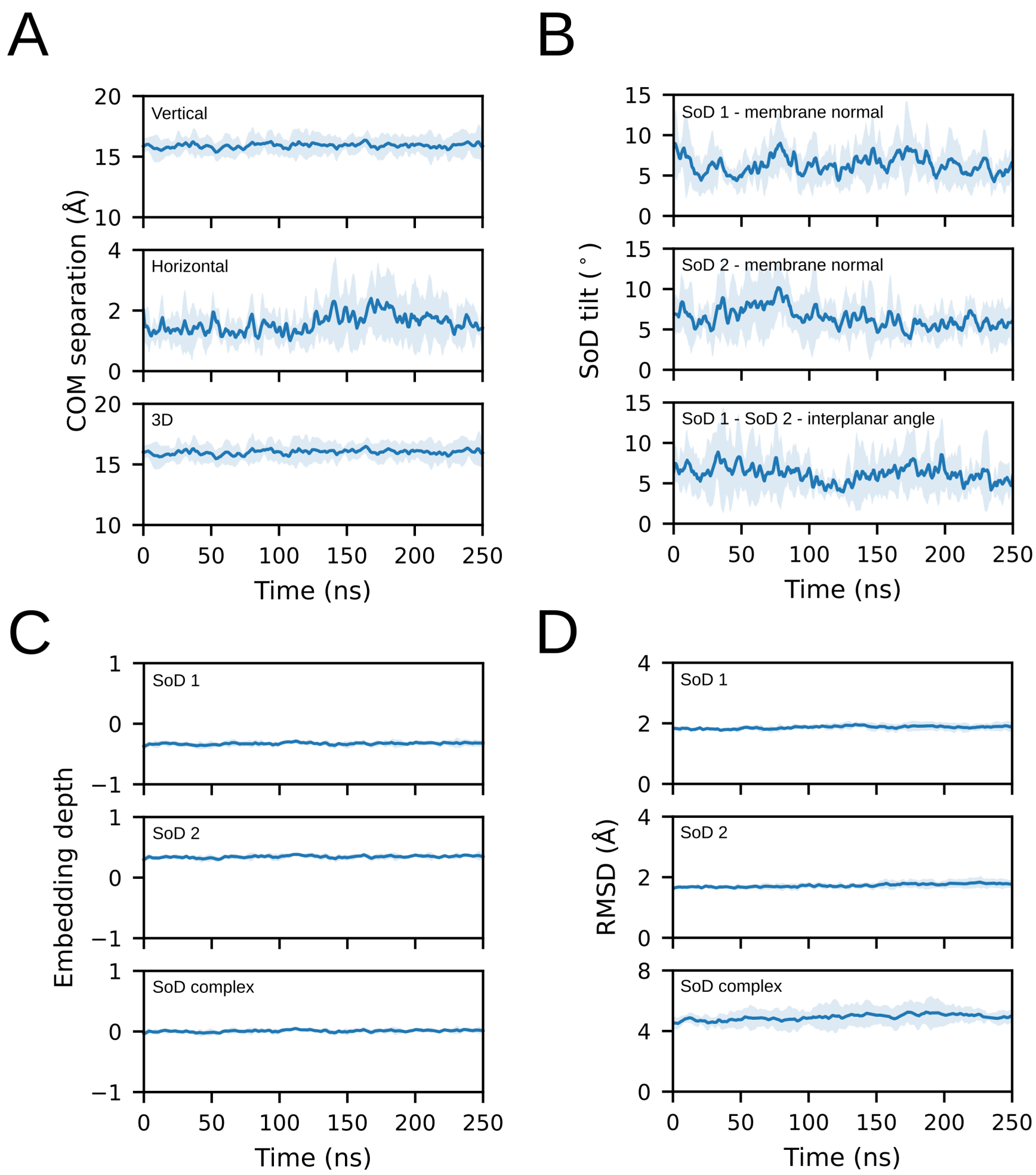

Figure S11. Structural stability of dimeric SoD assemblies.

(A) Centre-of-mass (COM) separation between the two SoDs along the membrane normal (top), in the membrane plane (middle), and in 3D (bottom). **(B)** Tilt of each SoD, defined as the angle between the SoD plane normal and the membrane normal (0° = parallel to the membrane plane; top, middle; see S4.3), and the angle between the two SoD planes (bottom). **(C)** Embedding depth of each SoD (top, middle) and of the complex (bottom), where 0 is the bilayer midplane and ±1 are the bulk phosphate layers. **(D)** RMSD of each SoD (top, middle) and of the complex (bottom) relative to the first frame, computed over organic SoD C and N

atoms. Lines show the mean across replicates (n = 6, +100 mV); shading shows SD. Simulations were performed without SoD COM restraints.

Across all replicates, the dimer remained intact and stacked over 250 ns (**Fig. S12**). The two SoDs stayed separated by ~16 Å along the membrane normal and were laterally offset by only ~1.5 Å, indicating a stable, co-axial stack spanning the membrane (**Fig. S12A**). Each SoD remained nearly parallel to the membrane plane (tilt ~5–7°), and the two SoDs remained nearly parallel to each other (interplanar angle ~5–7°) (**Fig. S12B**). The SoDs sat symmetrically about the bilayer midplane (embedding depths of approximately −0.35 and +0.35), placing the complex centre at the midplane (**Fig. S12C**). Individual SoD RMSDs remained ~2 Å throughout (**Fig. S12D**). The higher RMSD of the complex (~5 Å) reflects small relative motions of the two SoDs rather than structural drift, since it remained constant over time (**Fig. S12D**). Neither dissociation nor progressive reorientation was observed.

S8 Further Characterisation of Membrane Deformations and SoD-Lipid Channels and System Size Validation

Here we characterise the extent of the membrane deformation induced by pre-embedded SoD, the properties of the resulting SoD-lipid channels, and whether either depends on the size of the simulated membrane patch.

S8.1 Membrane-Wide Deformation

Compared with empty POPC:cholesterol (2:1) bilayers, pre-embedded SoD stabilised the formation of SoD-lipid toroidal pores, inducing radially-diminishing membrane thinning (**Fig. S12A**) and membrane curvature (**Fig. S12B**) within the local region, with POPC headgroups (P – N vector) tilting to follow the curved surface towards the membrane centre (**Fig. S12C**) (**Fig 1A**). POPC choline and phosphate headgroups were observed to form tight rings around the lumen of the SoD-lipid channel (**Fig. S12D, E**) consistent with toroidal pore formation.

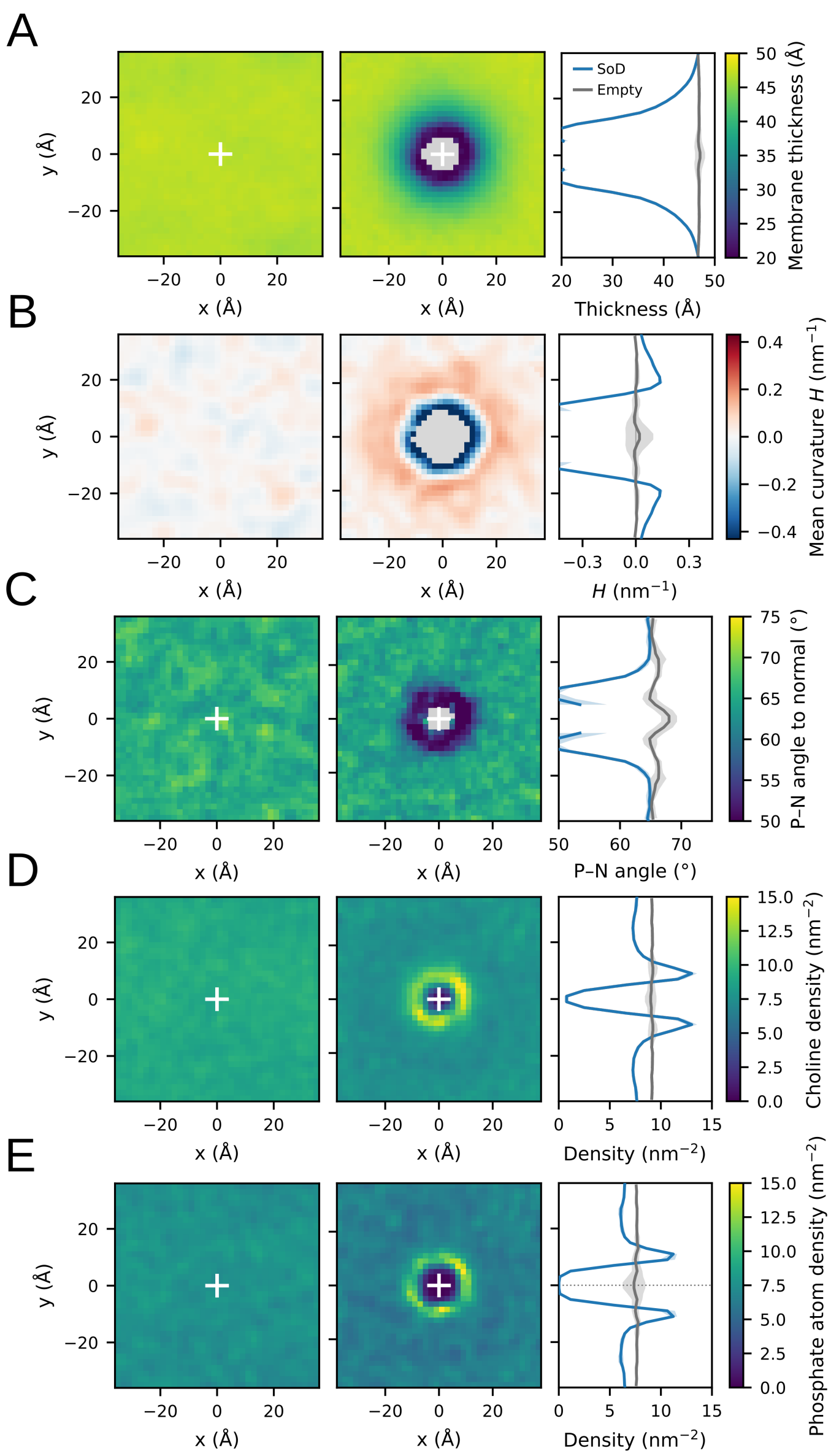

Figure S12. Membrane deformations observed within SoD-lipid pore containing systems.

(A) Membrane thickness (P-P) for 85 x 85 Å membrane patches with SoD absent (left), present (middle) with radial thickness profile (right). **(B)** Membrane curvature. **(C)** Lipid orientation (P - N). **(D)** Choline density**. (E)** Phosphate density.

S8.2 Channel Properties

Water filled the pore across the membrane (**Fig. S13A**), forming a continuous hydrogen-bonded network between both sides of the membrane with a mean occupancy of 94.4 ±1.1 % of frames, never seen in SoD-free membranes (**Fig. S13A**).

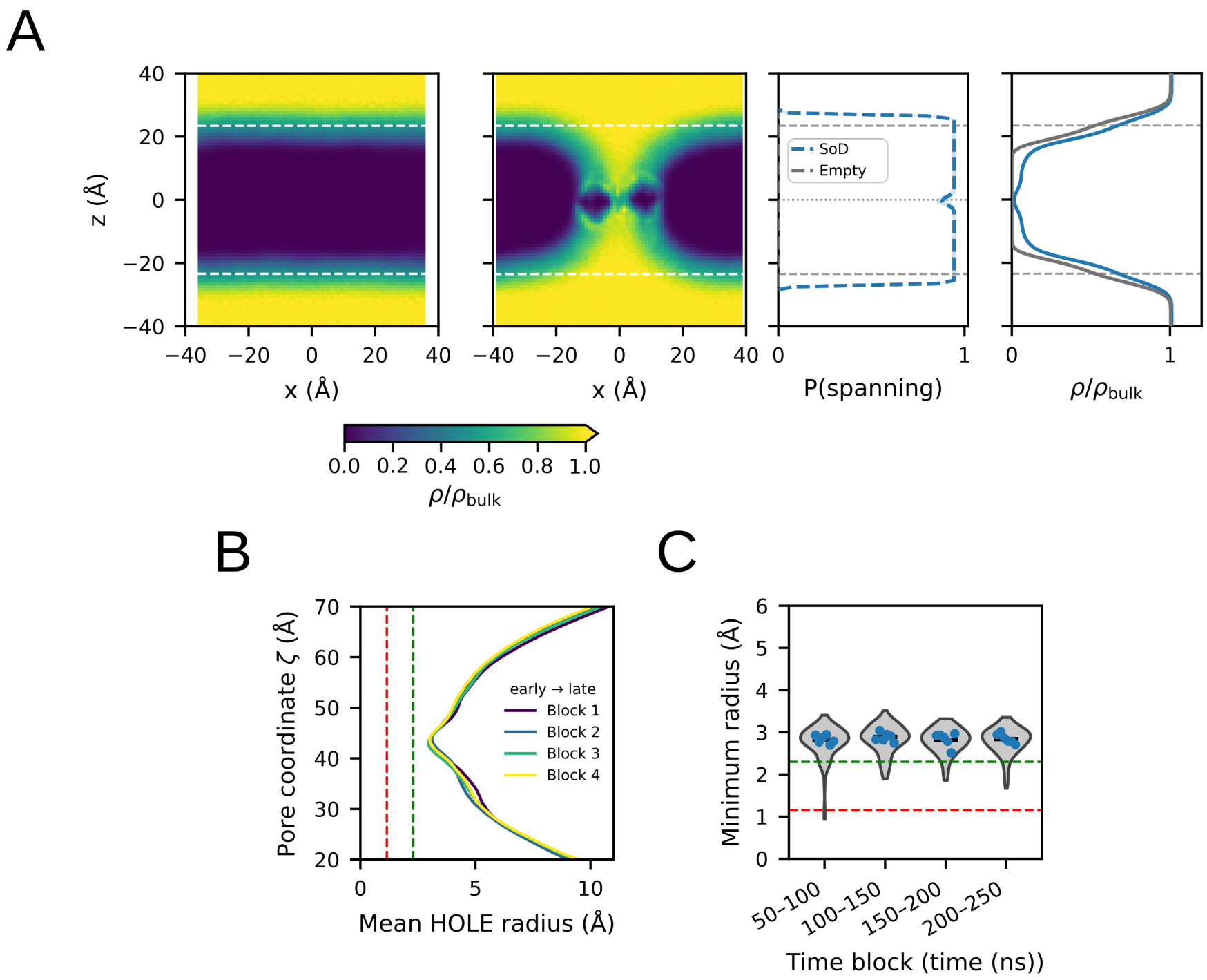

Figure S13. Configuration of solvated SoD-lipid toroidal pores.

(A) Water density plots calculated across all replicas (n = 6), with water-connectivity (P(spanning)) and water density (ρ/ρ_{bulk}). **(B)** Time-resolved HOLE pore radius profile. **(C)** Distribution of HOLE minimum radii across four consecutive 50 ns blocks.

S8.3 Finite Size Effects

To test whether finite-size effects influenced our results, we repeated the primary simulations in a 150 × 150 Å membrane patch and compared membrane deformation, conductance and pore stability with the 85 × 85 Å system.

S8.3.1 Periodic-image separation

Our primary set (COM-restrained SoD, 85 x 85 Å POPC:cholesterol (2:1), NBFIX, +100 mV, 250 ns replicates) was run in NVT following NPT equilibration at 79.3 ± 0.5 Å in x and y and 87.4 ± 1.1 Å in z (mean ± SD across replicates). The minimum distance between SoD heavy atoms and those of any of its 26 periodic images was 48.0 ± 0.5 Å (minimum over all frames: 44.7 Å).The closest approach of the membrane (any lipid or SoD heavy atom) to its periodic image along z was 24.1 ± 0.8 Å (minimum: 15.8 Å), with a 44.8 ± 0.7 Å water layer between phosphate planes. All separations exceed the 12 Å real-space cutoff for Lennard-Jones (force-switched from 10 Å) and short-range electrostatic interactions, so periodic images do not interact directly. Long-range electrostatics were treated with PME (See Methods).

S8.3.2 SoD-Lipid Pore Membrane Deformation

Fitting the radial thickness profile gives a plateau radius r*, beyond which the membrane is unperturbed, and a decay length ξ (**Table S20**). Both were larger in the 150 x 150 Å patch (r* = 35.4 vs 31.1 Å; ξ = 7.4 vs 6.2 Å). In the 85 x 85 Å patch, only 8.5 ± 1.4 Å separated r* from the box edge, and headgroup density beyond r* was 16% below the SoD-free membrane (6.4 vs 7.6 nm^{-2}), compared with 3% in the larger patch. Thickness beyond r* matched the SoD-free membrane in both. The 85 x 85 Å patch therefore under-represents the full extent of the deformation.

Table S20. Membrane perturbation in POPC:cholesterol (2:1) bilayers of two patch sizes.

Replica	Plateau radius (Å)	Decay length ξ (Å)	Half-box width (Å)	Margin (Å)	Far-field thickness (Å)	Membrane thickness (Å)		Lipid orientation		Headgroup density		Leaflet curvature	
						SoD-system	Control	SoD-system	Control	SoD-system	Control	SoD-system	Control
Monomeric, horizontal, POPC:cholesterol (2:1), NBFIX, 85 × 85 Å, 293 K													
1	32.97	6.75	39.82	6.85	47.21	46.78 ± 0.39		64.98 ± 26.72		6.40		0.08	
2	29.00	5.67	39.415	10.42	47.40	47.10 ± 0.34		64.82 ± 26.87		6.37		0.07	
3	32.12	6.46	39.75	7.62	47.12	47.01 ± 0.38		65.36 ± 26.63		6.45		0.06	
4	31.79	6.43	39.745	7.95	47.04	46.72 ± 0.37		64.85 ± 26.71		6.47		0.06	
5	31.68	6.32	39.78	8.09	47.02	46.68 ± 0.36		65.00 ± 26.86		6.21		0.06	
6	29.18	5.68	39.27	10.09	47.41	47.18 ± 0.35		65.43 ± 26.88		6.60		0.07	
**Mean**	**31.12 ± 1.64**	**6.22 ± 0.44**	**39.63 ± 0.23**	**8.50 ± 1.42**	**47.20 ± 0.17**	**46.91 ± 0.21**	**46.70 ± 0.38**	**65.07 ± 0.26**	**65.58 ± 0.11**	**6.36 ± 0.20**	**7.56 ± 0.10**	**0.07 ± 0.01**	**0.05 ± 0.01**
Monomeric, horizontal, POPC:cholesterol (2:1), NBFIX, 150 × 150 Å, 293 K													
1	37.32	7.95	66.84	29.52	47.15	47.07 ± 0.20		65.55 ± 27.27		7.34		0.07	
2	34.84	7.28	66.88	32.04	46.94	46.82 ± 0.15		65.60 ± 27.18		7.27		0.07	
3	35.90	7.51	66.65	30.74	47.22	47.21 ± 0.18		65.65 ± 27.18		7.30		0.06	
4	36.56	7.69	66.76	30.20	47.23	47.09 ± 0.15		65.69 ± 27.15		7.27		0.07	
5	32.85	6.81	66.26	33.41	47.27	47.27 ± 0.15		65.65 ± 27.30		7.39		0.07	
6	35.06	7.43	66.38	31.32	47.50	47.29 ± 0.16		65.76 ± 27.30		7.48		0.07	
**Mean**	**35.42 ± 1.56**	**7.44 ± 0.39**	**66.63 ± 0.26**	**31.21 ± 1.39**	**47.22 ± 0.18**	**47.12 ± 0.18**	**46.81 ± 0.27**	**65.65 ± 0.07**	**65.62 ± 0.15**	**7.34 ± 0.09**	**7.59 ± 0.06**	**0.07 ± 0.00**	**0.06 ± 0.00**

Empty, non-SoD systems used a radial exclusion radius, r = 0.

S8.3.3 Conductance and Stability of SoD in 150 x 150 Å Patches

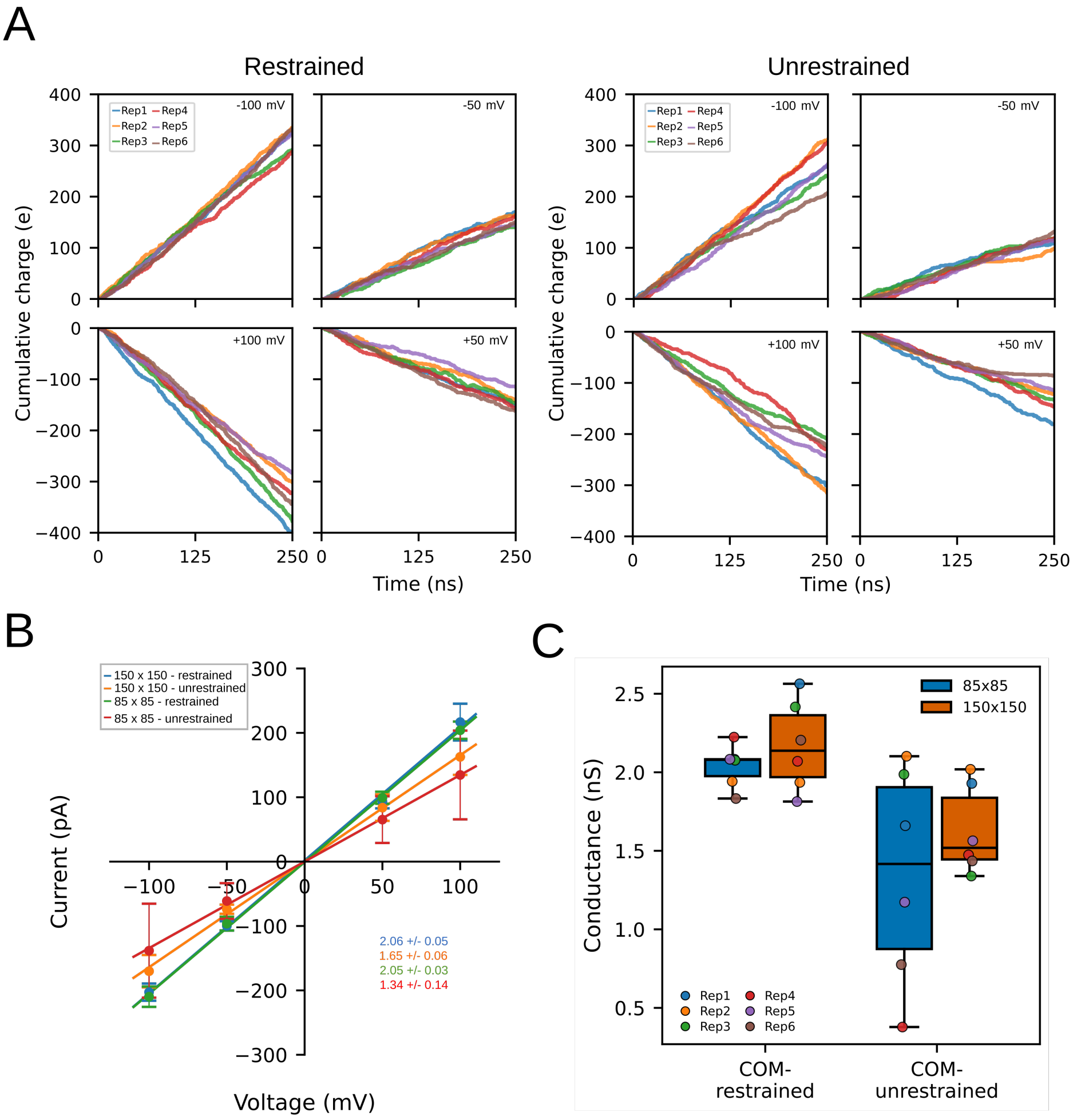

Figure S14. Conductances of SoD in 150 x 150 Å membranes.

(A) Cumulative charge traces for pre-embedded SoD in 150 x 150 Å POPC:cholesterol (2:1) membranes under transmembrane potentials of ± 50 mV and ± 100 mV in the presence and absence of COM-restraints (left and right, respectively). **(B)** current-voltage traces for COM-restrained and COM-unrestrained SoD simulations in 85 x 85 Å and 150 x 150 Å membrane patches, with conductance comparisons at +100 mV **(C)**. Replica colouring is consistent across all panels.

Conductances did not significantly differ between the two patch sizes in the presence or absence of COM restraints at +100 mV (COM-restrained: 85 × 85: 2.04 ± 0.14 nS; 150 × 150: 2.17 ± 0.29 nS; permutation test, $p = 0.366$; COM-unrestrained: 85 x 85: 1.35 ± 0.69 nS; 150 x 150: 1.63 ± 0.28 nS; permutation test, $p = 0.381$). However, there is a larger spread in the

COM-unrestrained 85 × 85 Å system, which comes primarily from three replicates (Rep4-6), whose conductances are much lower than the rest (0.4-1.2 nS, vs 1.7-2.1 nS; **Fig. S14C**). These same replicates show reduced cumulative charge transfer at every applied voltage (**Fig. S14A**), while the remaining three COM-unrestrained replicates conduct at levels comparable to the COM-restrained simulations. We compared mean lipid occlusion and embedding depth across all 6 COM-unrestrained replicates of both patch sizes at +100 mV transmembrane potentials (**Fig. S15A, B**). Replicates 4-6 of the 85 × 85 Å system showed the highest lipid occlusion and the shallowest embedding, with replicate 4, the least conductive, the most extreme on both measures.

In the COM-unrestrained systems, mean lipid occlusion (permutation test, p = 0.498) and mean embedding depth (permutation test, p = 0.71) did not differ significantly between patch sizes. In COM-restrained simulations, mean SoD tilt was lower and less variable in the larger patch (150 × 150 Å: 10.1 ± 2.7°; 85 × 85 Å: 13.6 ± 4.0°), although the difference was not statistically significant (permutation test, p = 0.097), suggesting that SoD tilt may be modestly sensitive to patch size. Because conductance did not change significantly when the lateral box area was increased approximately three-fold, the lateral box size is unlikely to contribute substantially to access resistance. Overall, a larger patch is preferable, both for agreement with the SoD-free control membrane in bulk headgroup density and for more consistent SoD tilt. Nevertheless, we conclude that the 85 × 85 Å system is sufficient for the primary pore conclusions, but it is not sufficiently large to obtain a quantitatively converged estimate of the full spatial extent of membrane deformation.

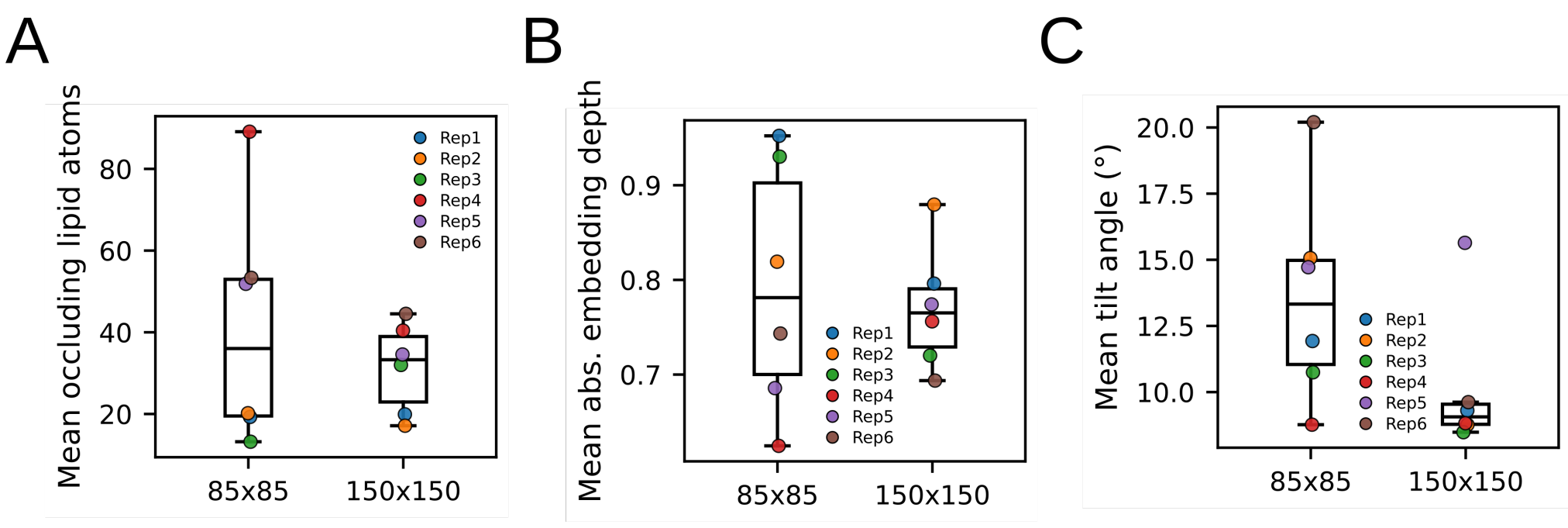

Figure S15. Comparison of channel stability metric means between SoD systems with 85 x 85 Å or 150 x 150 Å membrane patches.

Lipid occlusions (Permutation test, p = 0.498) (A), Embedding depths (Permutation test, p = 0.708) (B) and tilt angles (Permutation test, p = 0.0974) (C) for SoD simulations under +100 mV transmembrane potentials with 85 x 85 Å and 150 x 150 Å membrane patches. Permutation t-tests were two-sided.

S9 Further Characterisation of SoD-Lipid Channel Chloride Permeation Mechanisms

To examine how Cl^- crosses the membrane, we analysed every Cl^- crossing in the COM-restrained, pre-embedded SoD simulations in POPC:cholesterol (2:1) at +100 mV. For each crossing we determined the route, the degree of dehydration, the coordinating groups, and contact lifetimes.

S9.1 Definition of first-shell contacts and Cl- permeation routes

Contact distances were taken as the first minimum of each Cl^- RDF (0-12 Å, 0.05 Å bins, mean of six replicates; **Fig. S17, Table S22**). Only the pore-lining bipyridine H4/H4′ hydrogens formed a distinct contact shell with Cl^-; other SoD hydrogens gave only a shoulder. Cholesterol O3 contacts were rare (g ≈ 0.1). For residence times, SoD contact was defined as Cl^- within 4.2 Å of any SoD heavy atom.

A crossing was assigned to the SoD cavity if, as the ion crossed the mean SoD plane, SoD heavy atoms within 8 Å in z surrounded it with no angular gap > 120°. All other crossings would have been assigned to the SoD periphery (within 4.2 Å of the SoD) or to the lipid-lined pore; none occurred.

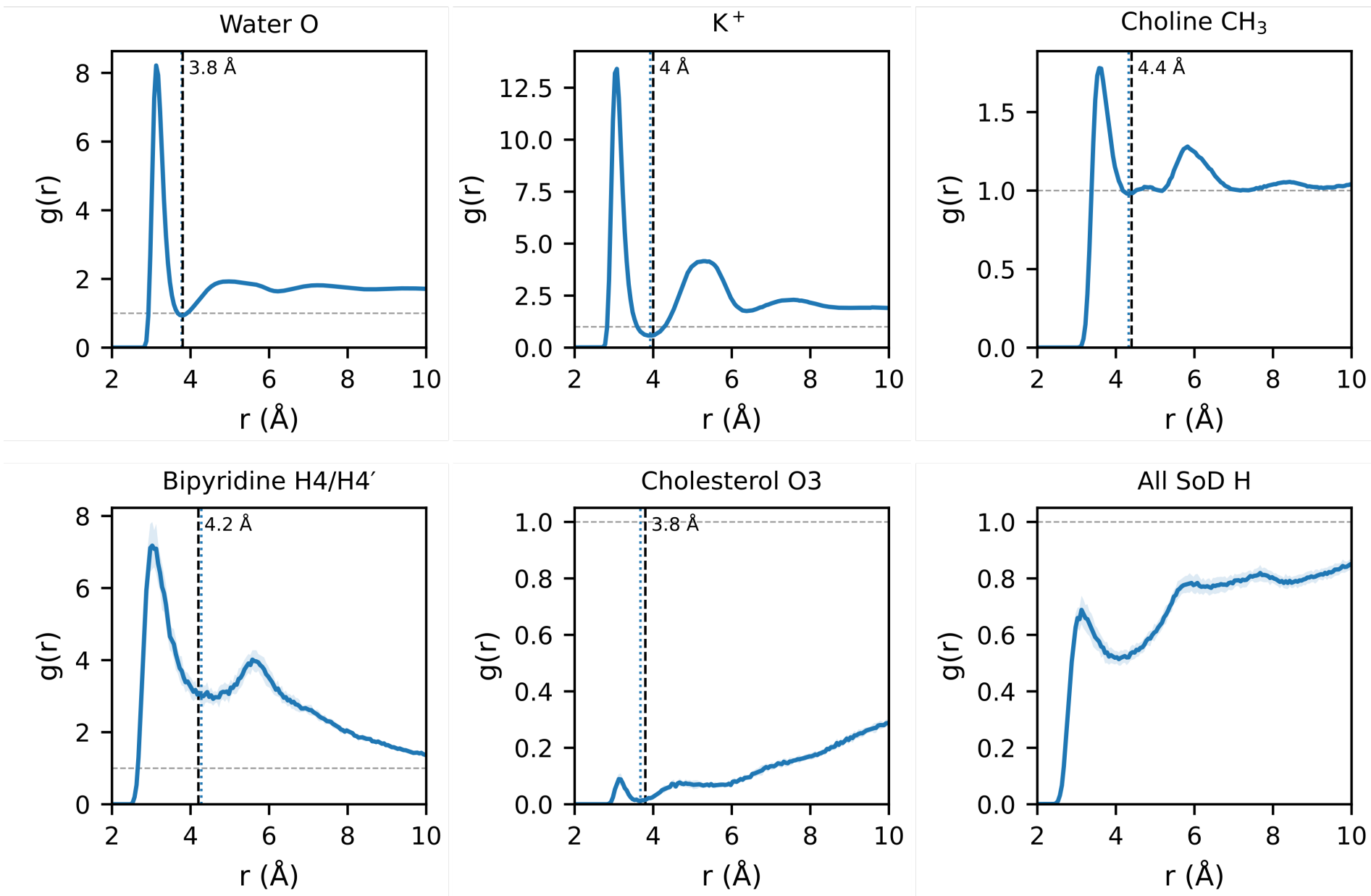

Figure S16. Cl$^-$ - RDFs with water, K+, POPC choline, cholesterol and SoD hydrogens, used to define contact distances.

Table S21. RDF first peak and minima (contact distances) for interactions used to derive contact cutoff distances.

Partner (atoms)	First peak (Å)	First minimum (Å)	Cutoff used (Å)
Water (O)	3.18	3.78	3.78
K+	3.08	3.93	3.93
POPC choline (methyl C13-C15)	3.63	4.33	4.33
SoD cavity-lining H (4/4’)	3.08	4.28	4.28
Cholesterol (O)	3.18	3.68	3.68

S9.2 Chloride permeation across SoD-lipid channels

All 1,916 Cl^- crossings, in all six replicates, passed through the central SoD cavity (**Fig. S17A**, **B**). Cl^- was only partially dehydrated with lost waters replaced by contacts with the pore (**Fig. S17C**). Choline methyl groups coordinated Cl^- at the membrane entry and exit, and the bipyridine H4/H4′ hydrogens coordinated it in the membrane core; SoD and choline contacts lasted are shown in **Figure S17D**. Cl^--K^+ contacts fell to zero near the pore midplane (**Fig. S17C**), so Cl^- permeates SoD as a free anion.

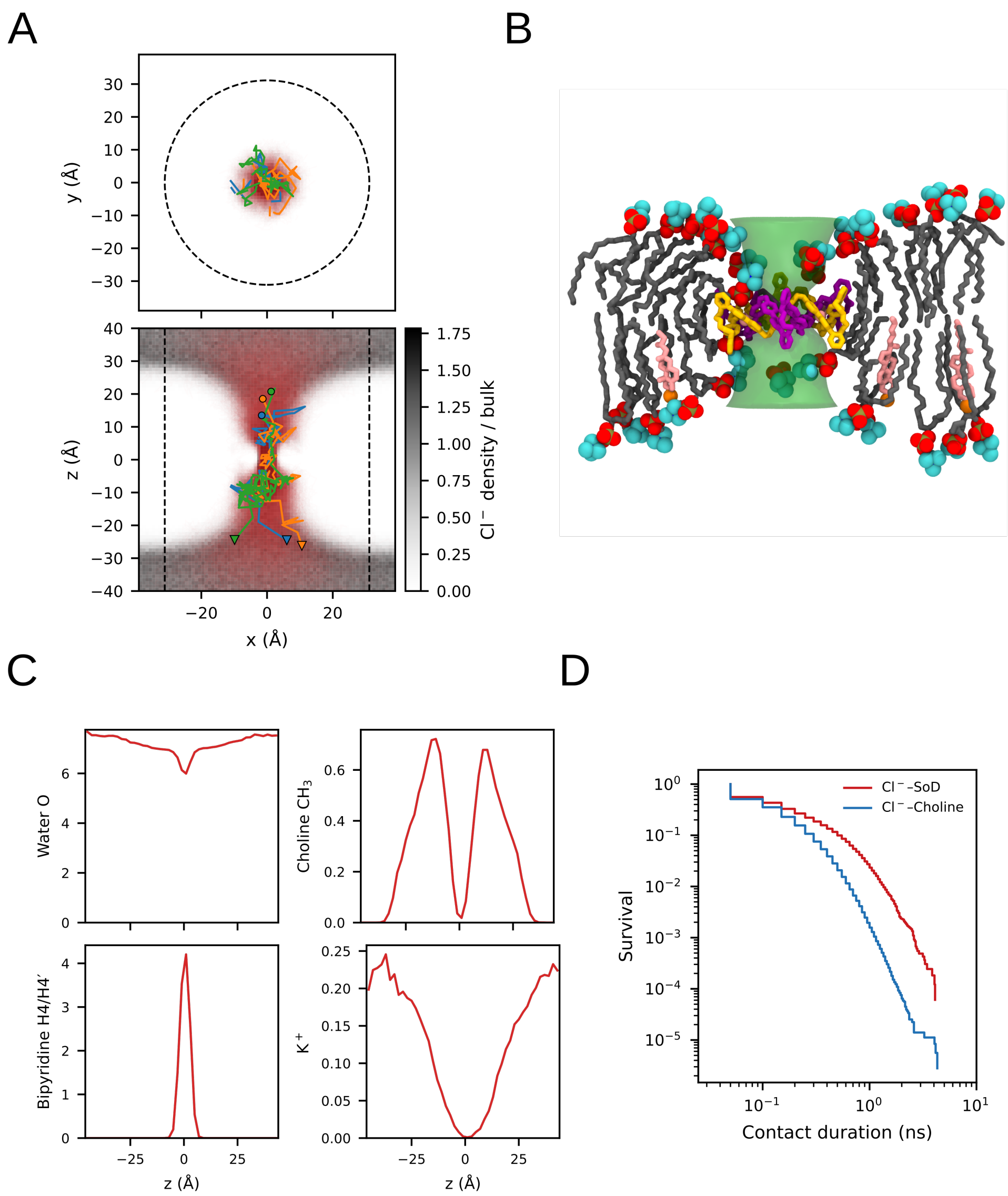

Figure S17. Insights into chloride permeation through SoD centre for COM-restrained SoD under +100 mV transmembrane voltages.

(A) Three example Cl- pathways across SoD-lipid channels. Visualisations shown for x/y plane (top) and x,z plane (bottom). All 1,916 Cl- crossings across the 6 replicas shown in red. Circle

and arrow symbols indicate the first and final frames of the example permeation paths, respectively. **(B)** Smoothed projected chloride densities shown with a reference SoD-lipid structure to highlight chloride density passes through the centre of the SoD catenane. **(C)** Cl^- interactions with water (O atoms) (top left), POPC choline groups (methyl C atoms) (top right), interactions between Cl^- and bipyridine 4/4' hydrogens lining the pore (bottom left), and **(D)** contact duration/residence time for Cl^- SoD (red) and Cl^- - POPC choline (blue) interactions.

S10 Further details of phosphatidylcholine membrane thickness assay

To probe the influence of membrane thickness on the conductance of SoD-lipid channels, simulations were run with SoD pre-embedded in a series of saturated phosphatidylcholine lipid bilayers with varying thicknesses, namely; DDPC, DCPC, DLPC, and DMPC, chosen specifically for their variation in chain length (**Fig S18A**), allowing for a range of different membrane thicknesses to be tested (**Fig S18B**).

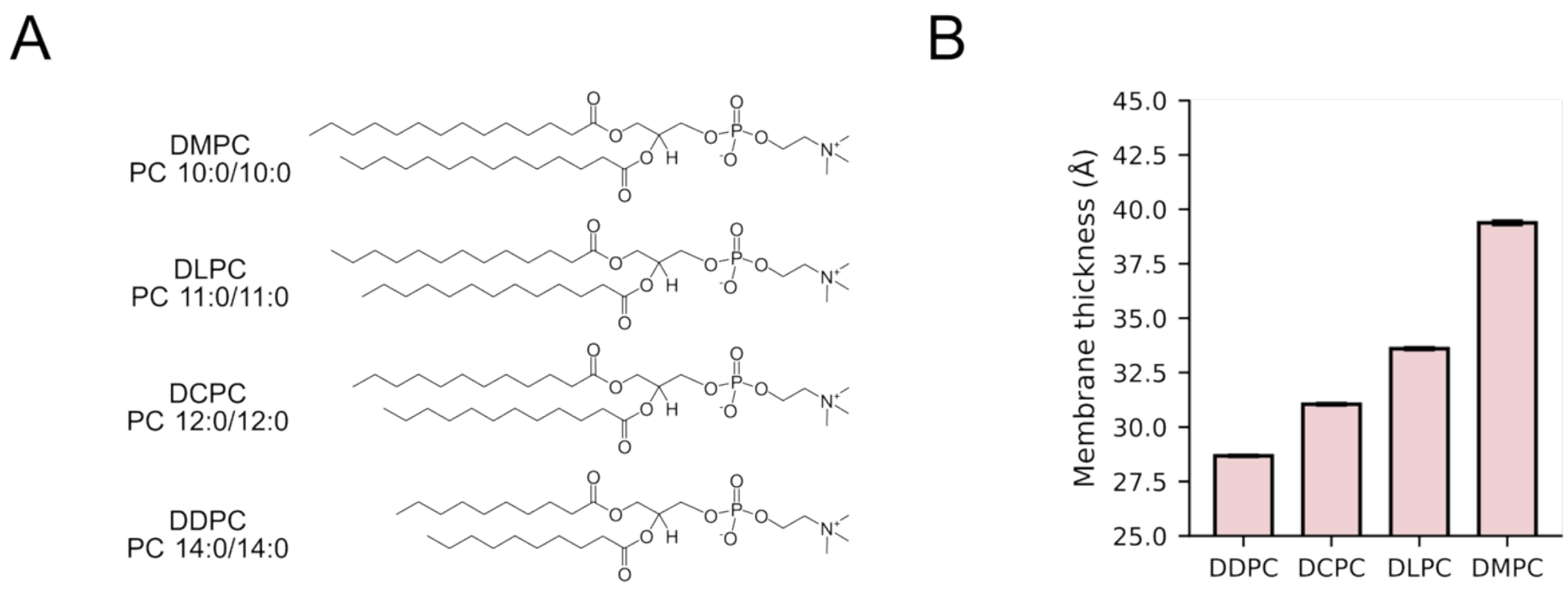

Figure S18. Phosphatidylcholine membrane thickness assay composition.

Lipid structures to highlight difference in length **(A)** and membrane thicknesses **(B).**

S11. Electropore-mediated SoD insertion

To test whether a pre-formed membrane defect allows the SoD to insert into the bilayer, we simulated the SoD alongside an electropore in a DPhPC bilayer (**Fig. S22A**; system preparation is described in Methods).

S11.1. Definitions and contact traces

For this investigation, we defined and analsyed key metrics as followed:

SoD - lipid contacts: contacting lipids were defined as those with heavy atoms within 4 Å (inclusive) of any SoD heavy atom.

COM-separation ($|\Delta z|$): The z-axis difference in COM between all SoD atoms and DPhPC P atoms.

SoD tilt: See **section S4.3**.

DPhPC bilayer bulk thickness: determined as in S4.1, with radial distances measured from the electropore axis instead of the SoD. The pore axis was taken in each frame as the mean xy position of P atoms within 5 Å of the bilayer midplane. The bulk thickness was the mean P-P distance across the region where the fitted thickness profile lies within 2% of its plateau ($r > r_{excl}$), rather than the fitted T_{bulk} itself. For the fit, T_{bulk} was bounded between 0.5× the smallest and 1.5× the largest measured thickness, and ξ by the width of the fit window. Bilayers split across the periodic boundary in z were handled by locating the midplane as the circular mean of the P atom z positions and assigning leaflets by minimum-image offset from it. Replicates with a pore in fewer than half of the frames were treated as pore-free, and the whole bilayer was used. The membrane half-thickness, z_P was defined as half the bulk thickness.

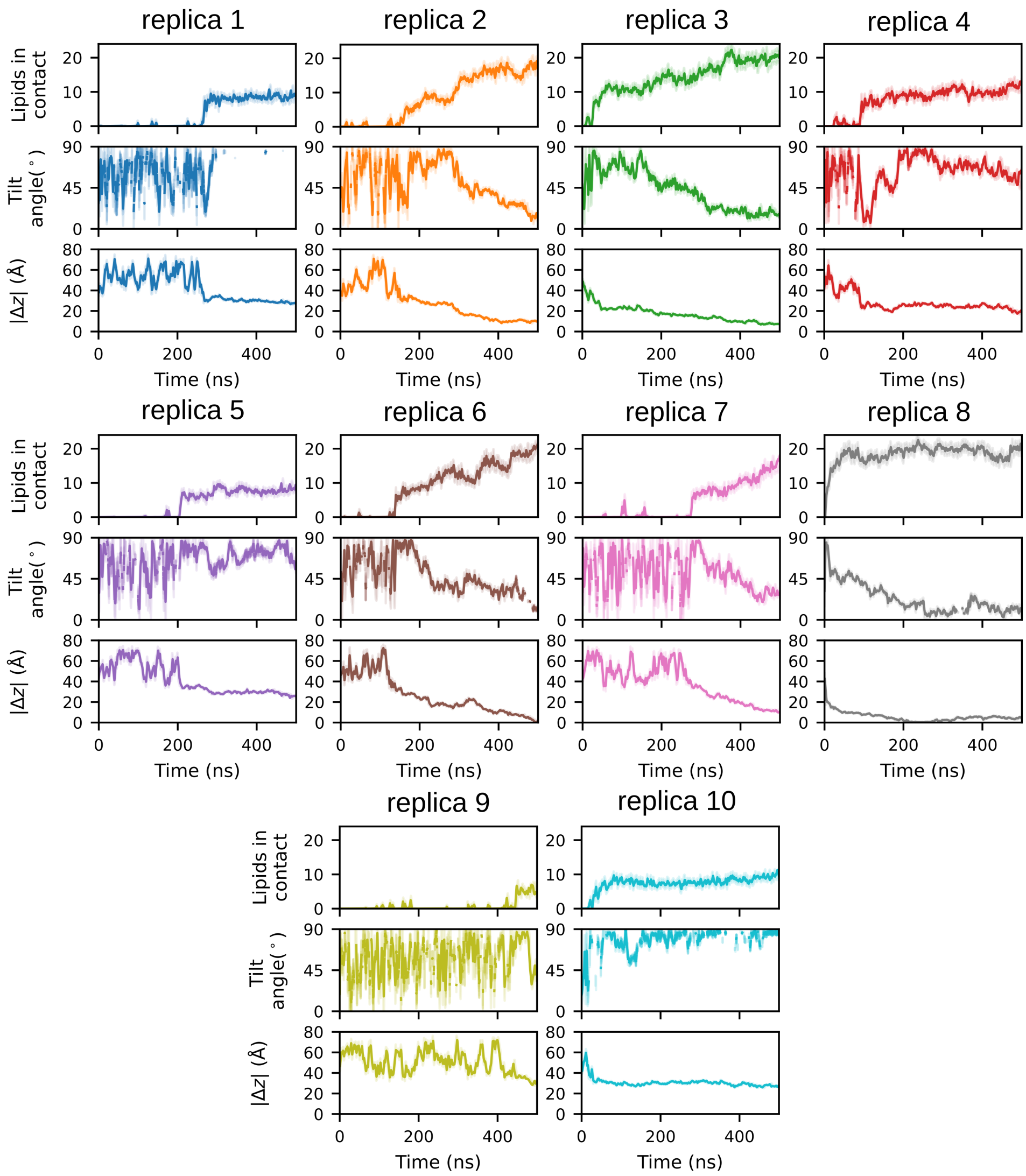

Figure S19. Insertion metric traces for SoD - electropore simulations in the presence of a +0.035 V nm^{-1} stabilising electric field.

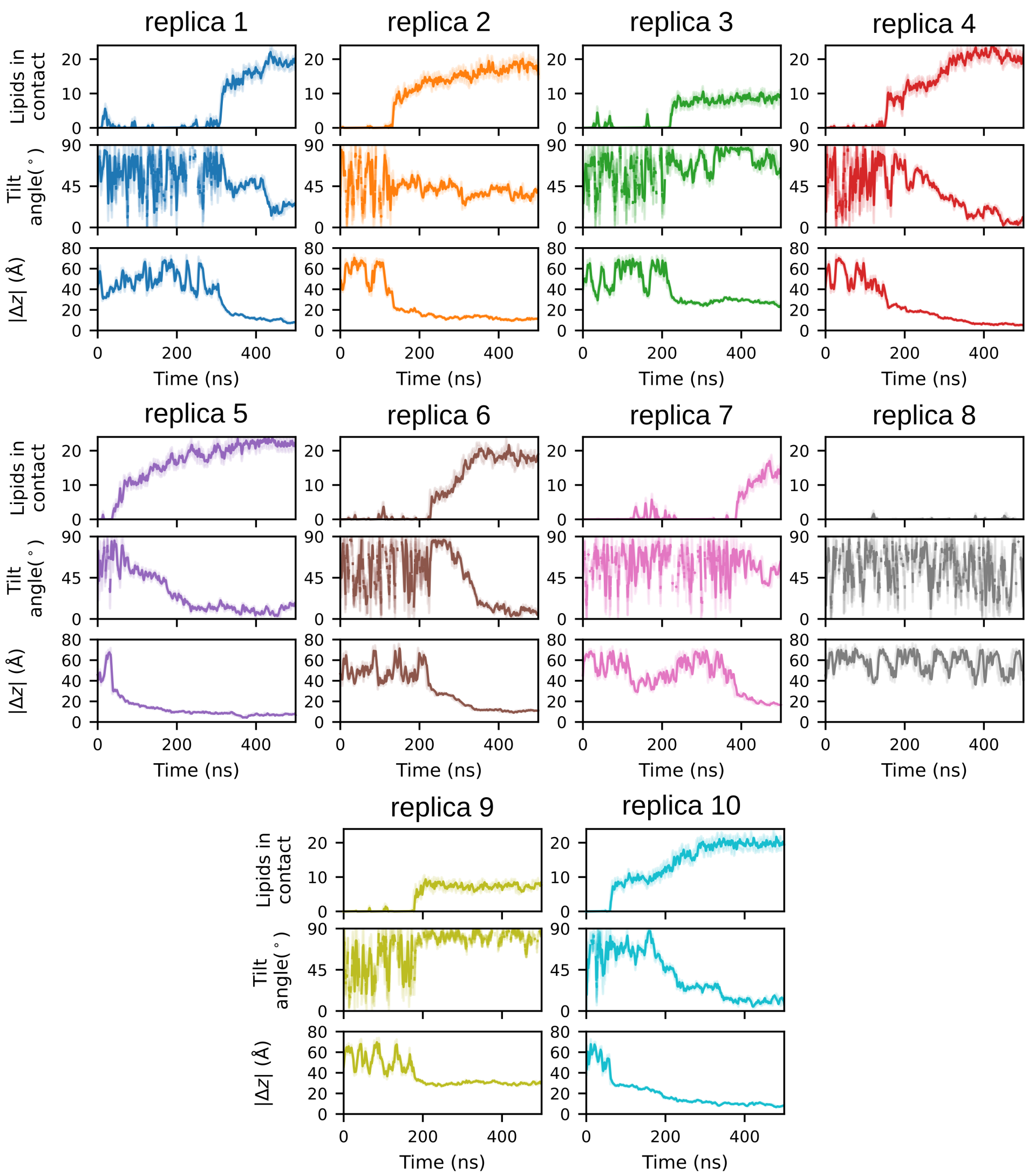

Figure S20. Insertion metric traces for SoD - electropore simulations in the presence of a -0.035 V nm^{-1} stabilising electric field.

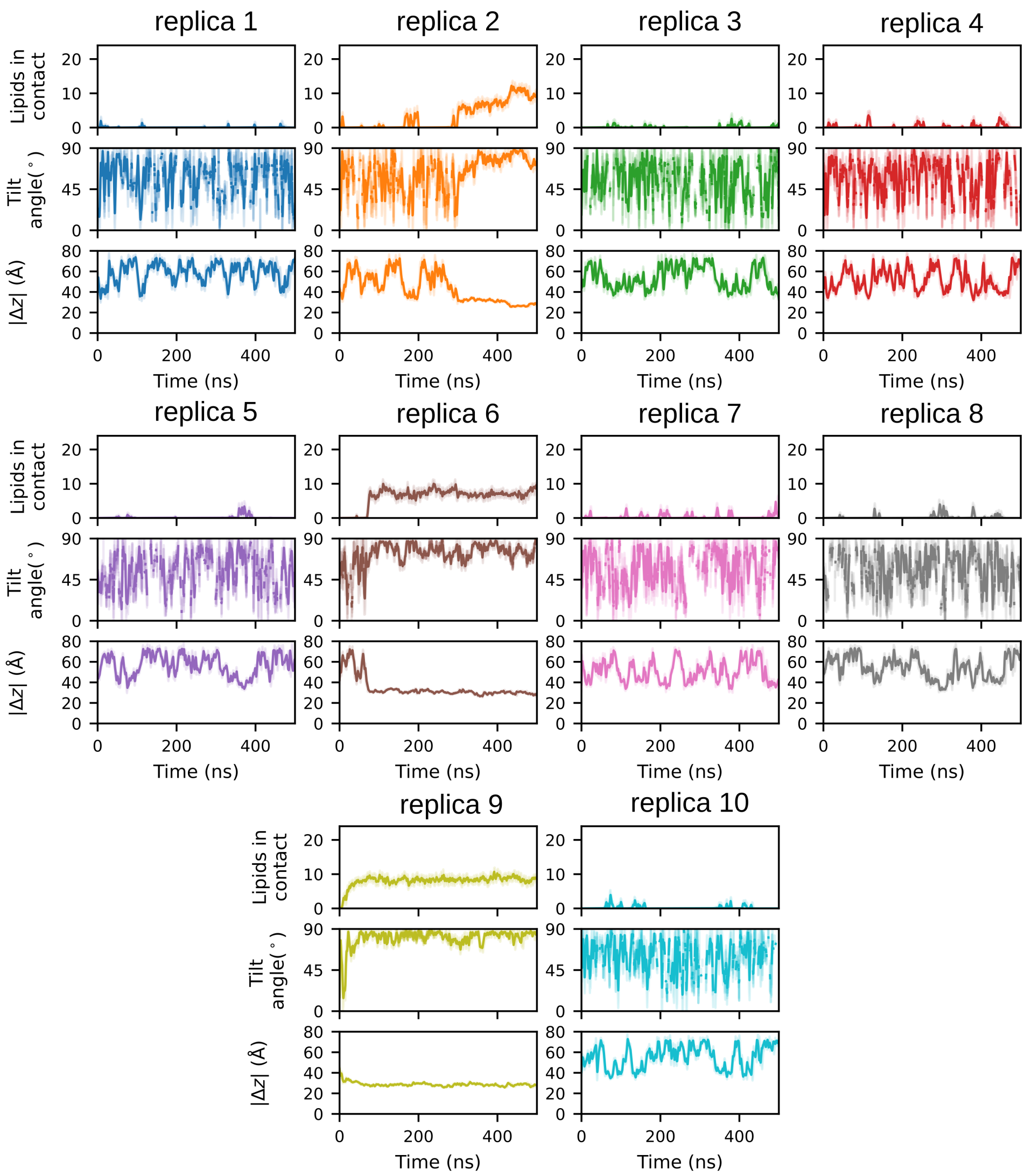

Figure S21. Insertion metric traces for SoD - electropore simulations in the absence of a stabilising electric field.

Table S22. Membrane thickness parameters for ± 0.035 V nm^{-1} simulations.

Electric potential (V nm^{-1})	r_{excl}	T_{bulk}	T_{direct}	Z_P
-0.035	26.43 ± 1.75	38.88 ± 0.17	38.54 ± 0.13	19.27 ± 0.07
+0.035	27.84 ± 2.08	37.42 ± 0.09	38.54 ± 0.21	18.71 ± 0.04
**Mean**	27.12 ± 0.71	38.15 ± 0.73	38.54 ± 0.00	18.99 ± 0.28

S11.2 Bulk bilayer thickness and definition of insertion

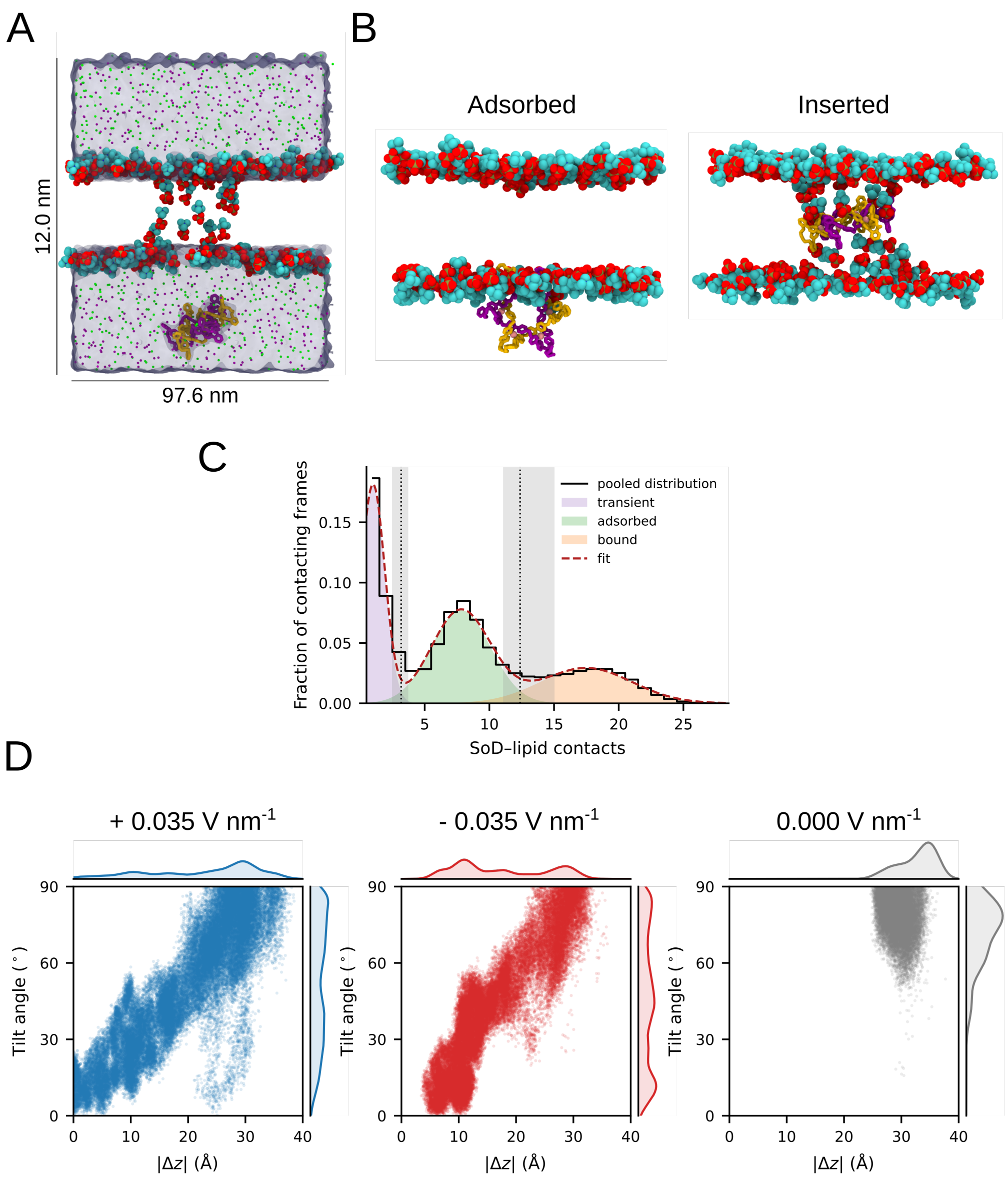

Figure S22. SoD - electropore insertion simulations.

(A) Initial system configuration. **(B)** Two predominantly observed SoD-bilayer configurations with SoD adsorbed (left) or inserted (right). **(C)** Distribution of the fraction of frames wherein varying numbers of lipids (DPhPC) contact SoD. **(D)** Tilt angle vs |Δz| plots with accompanying densities.

Visual inspection of the SoD-electropore trajectories suggested two predominant SoD-lipid interaction configurations (**Fig. S22B**). In the first ("adsorbed"), SoD associated with the bilayer headgroup region in a predominantly vertical orientation. In the second ("inserted"), SoD was

more deeply embedded within the bilayer in a predominantly horizontal orientation. The |Δz|, tilt-angle and lipid-contact traces changed together as the SoD became embedded (**Figs. S19-S21**). |Δz| decreased steadily as the number of contacting lipids rose, and the transition to the highest-contact state coincided with a sharp drop in tilt angle. This is consistent with deeper insertion and reorientation of the SoD within the bilayer.

The distribution of contacting-lipid numbers (frames with ≥ 1 contact, pooled over replicates and conditions) showed three populations, fitted with Gaussian components centred at 1.0, 7.8 and 17.5 contacts (**Fig. S20C**). Boundaries at 3.2 (95% CI 2.5-3.7) and 12.4 (95% CI 11.1-15.0) contacts define transient (1-3), adsorbed (4-12) and deeply bound (≥ 13) states. Medians are over pooled frames. Adsorbed frames had a median tilt of 73.0° (IQR 60.4-82.0°) and |Δz| of 28.4 Å (IQR 25.4-30.4 Å). Deeply bound frames had a median tilt of 24.6° (IQR 13.3-38.8°) and |Δz| of 10.2 Å (IQR 7.3-12.7 Å). The deeply bound state was observed almost exclusively in the presence of an applied field: 25.0% and 32.2% of frames at +0.035 and −0.035 V nm^{-1}, versus 0.1% without a field.

A frame was classed as inserted when the SoD contacted ≥ 13 lipids and its centre of mass had crossed the bulk phosphate plane into the bilayer interior ($|\Delta z| < z_P = 19.0$ Å). The two criteria largely coincided. Of frames with ≥ 13 contacts, 97-99% lay below z_P (97.5% pooled), and 89-90% of frames below z_P had ≥ 13 contacts. With either field, frames spanned a continuum from the adsorbed configuration (large |Δz|, high tilt) to the inserted configuration (small |Δz| , low tilt) (**Fig. S22D**). Insertion occurred in 6/10 and 7/10 replicates at +0.035 and −0.035 V nm^{-1} (24% and 32% of frames), and in none of the 10 replicates without a field (Fisher's exact test, both fields vs no field, $p = 0.001$). Without a field, the SoD remained outside the phosphate plane in an upright orientation throughout (**Fig. S20D, right**); the few high-contact frames (0.1% of frames, two replicates) had a median |Δz| of 26.1 Å and tilt of 86.6°.

S12. Additional Statistical Analysis

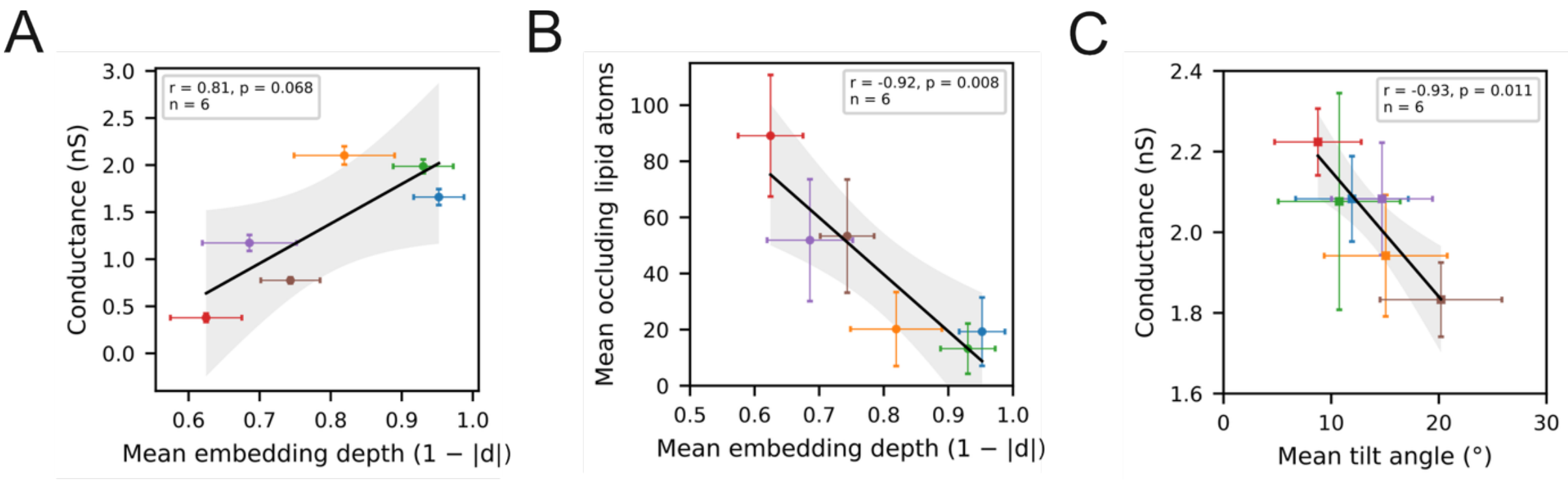

Figure S23. Relationships between pore stability metrics and conductance across replicates.

Each point is one replicate (n = 6) of pre-embedded SoD in POPC:cholesterol (2:1) under +100 mV (NBFIX). Colours identify replicates consistently across panels. Circles: COM-unrestrained **(A, B)**; squares: COM-restrained (C). **(A)** Conductance vs mean embedding depth (1 − |d|, where 1 is the bilayer midplane). **(B)** Mean number of lipid atoms within a 5 Å-radius cylinder centred on the SoD vs mean embedding depth. **(C)** Conductance vs mean SoD tilt angle (angle between the Fe(II)-plane normal and the membrane normal). Conductance error bars are the SEM across five time blocks; all other error bars are the SD of per-frame values within each trajectory. Black lines show ordinary least-squares fits with 95% confidence bands, as a visual guide. r is the Pearson correlation coefficient; p is a two-sided exact permutation test (all 720 orderings), so no normality assumption is needed.

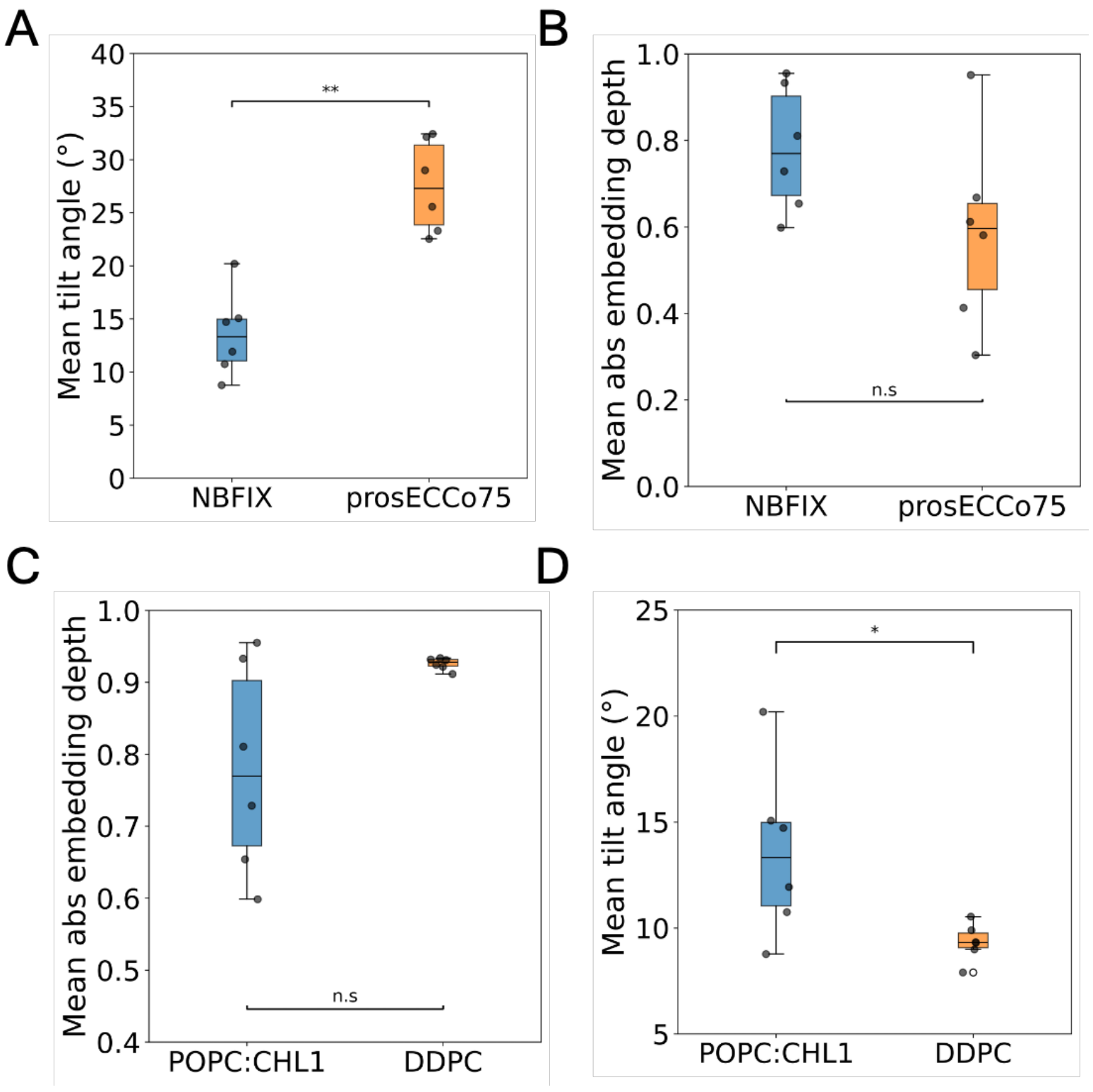

Figure S24. Summary box plots comparing SoD tilt and embedding depth across force-field and membrane-composition conditions.

(A-B) Comparisons between systems treated with either NBFIX or prosECCo75 charge handling methods. **(A)** COM-restrained prosECCo75 simulations exhibited significantly increased mean SoD tilt angles relative to NBFIX simulations (*permutation test, p* = 0.0022). **(B)** COM-unrestrained prosECCo75 simulations showed no statistically significant difference in mean absolute normalised embedding depth relative to NBFIX simulations (*permutation p* = 0.1147) **(B). (C-D)** Comparisons between NBFIX-treated systems with SoD embedded in either POPC:CHL1 or DDPC membranes. **(C)** No statistically significant difference in mean absolute normalised embedding depth was observed between POPC:CHL1 and DDPC systems in COM-unrestrained simulations (*permutation p* = 0.0584). **(D)** COM-restrained DDPC simulations exhibited significantly reduced mean SoD tilt angles relative to POPC:CHL1 simulations (*permutation p* = 0.0216).

References

1. Leigh, D.A., R.G. Pritchard, and A.J. Stephens, *A Star of David catenane.* Nat Chem, 2014. **6**(11): p. 978–82.
2. Won, Y., *Force Field for Monovalent, Divalent, and Trivalent Cations Developed under the Solvent Boundary Potential.* Journal of Physical Chemistry A, 2012. **116**(47): p. 11763–11767.
3. Vanommeslaeghe, K. and A.D. MacKerell, Jr., *Automation of the CHARMM General Force Field (CGenFF) I: bond perception and atom typing.* J Chem Inf Model, 2012. **52**(12): p. 3144–54.
4. Kim, S., et al., *CHARMM-GUI ligand reader and modeler for CHARMM force field generation of small molecules.* Journal of Computational Chemistry, 2017. **38**(21): p. 1879–1886.
5. Vanommeslaeghe, K., et al., *CHARMM general force field: A force field for drug-like molecules compatible with the CHARMM all-atom additive biological force fields.* J Comput Chem, 2010. **31**(4): p. 671–90.
6. Michaud-Agrawal, N., et al., *MDAnalysis: a toolkit for the analysis of molecular dynamics simulations.* J Comput Chem, 2011. **32**(10): p. 2319–27.
7. MacKerell, A.D., et al., *All-atom empirical potential for molecular modeling and dynamics studies of proteins.* J Phys Chem B, 1998. **102**(18): p. 3586–616.
8. Tubiana, T., et al., *TTClust: A Versatile Molecular Simulation Trajectory Clustering Program with Graphical Summaries.* J Chem Inf Model, 2018. **58**(11): p. 2178–2182.
9. Neese, F., *Software update: The ORCA program system-Version 5.0.* Wiley Interdisciplinary Reviews-Computational Molecular Science, 2022. **12**(5).
10. Grimme, S., et al., *r2SCAN-3c: A "Swiss army knife" composite electronic-structure method.* Journal of Chemical Physics, 2021. **154**(6).
11. Pettersen, E.F., et al., *UCSF ChimeraX: Structure visualization for researchers, educators, and developers.* Protein Science, 2021. **30**(1): p. 70–82.
12. Daku, L.M.L. and A. Hauser, *Ab Initio Molecular Dynamics Study of an Aqueous Solution of [Fe(bpy)3](Cl)2 in the Low-Spin and in the High-Spin States.* Journal of Physical Chemistry Letters, 2010. **1**(12): p. 1830–1835.
13. Dick, S., *Crystal structure of tris(2,2'-bipyridine)iron(II) bis(hexafluorophosphate), (C10H8N2)3Fe(PF6)2.* Zeitschrift Fur Kristallographie-New Crystal Structures, 1998. **213**(2): p. 356–356.
14. Chelucci, G. and R.P. Thummel, *Chiral 2,2'-bipyridines, 1,10-phenanthrolines, and 2,2':6',2''-terpyridines: syntheses and applications in asymmetric homogeneous catalysis.* Chem Rev, 2002. **102**(9): p. 3129–70.
15. Jerzy Kalenik, Z.P., *Solvent influence on the conformational equilibrium of 2,2′-bipyridine.* Journal of Molecular Liquids, 2005. **121**(2–3): p. 63–68.
16. Eric V. Dose, L.J.W., *Synthesis and electrochemical and photoemission properties of mononuclear and binuclear ruthenium(II) complexes containing 2,2'-bipyridine, 2,9-dimethyl-1,10-phenanthroline, 2,2'-bipyrimidine, 2,2'-biimidazole, and 2-pyridinecarboxaldimine ligands.* Inorg. Chem, 1978. **17**(9): p. 2660–2666.
17. Kalyanasundaram, K., *Photophysics, photochemistry and solar energy conversion with tris(bipyridyl)ruthenium(II) and its analogues.* Coordination Chemistry Reviews, 1982. **46**: p. 159–244.

18. August, D.P., et al., *Transmembrane Ion Channels Formed by a Star of David [2]Catenane and a Molecular Pentafoil Knot*. J Am Chem Soc, 2020. **142**(44): p. 18859–18865.
19. Virtanen, P., et al., *SciPy 1.0: fundamental algorithms for scientific computing in Python*. Nat Methods, 2020. **17**(3): p. 261–272.
20. Flyvbjerg, H. and H.G. Petersen, *Error-Estimates on Averages of Correlated Data*. Journal of Chemical Physics, 1989. **91**(1): p. 461–466.
21. Smart, O.S., et al., *HOLE: a program for the analysis of the pore dimensions of ion channel structural models*. J Mol Graph, 1996. **14**(6): p. 354–60, 376.
22. Laskowski, R.A., *SURFNET: a program for visualizing molecular surfaces, cavities, and intermolecular interactions*. J Mol Graph, 1995. **13**(5): p. 323–30, 307–8.
23. Wu, Y.C., et al., *A dc Method for the Absolute Determination of Conductivities of the Primary Standard KCl Solutions from 0 degrees C to 50 degrees C*. J Res Natl Inst Stand Technol, 1994. **99**(3): p. 241–246.
24. Prajapati, J.D., et al., *Computational Modeling of Ion Transport in Bulk and through a Nanopore Using the Drude Polarizable Force Field*. J Chem Inf Model, 2020. **60**(6): p. 3188–3203.
25. Nencini, R., et al., *Effective Inclusion of Electronic Polarization Improves the Description of Electrostatic Interactions: The prosECCo75 Biomolecular Force Field*. J Chem Theory Comput, 2024. **20**(17): p. 7546–7559.
